# Kiwi genome provides insights into evolution of a nocturnal lifestyle

**DOI:** 10.1186/s13059-015-0711-4

**Published:** 2015-07-23

**Authors:** Diana Le Duc, Gabriel Renaud, Arunkumar Krishnan, Markus Sällman Almén, Leon Huynen, Sonja J. Prohaska, Matthias Ongyerth, Bárbara D. Bitarello, Helgi B. Schiöth, Michael Hofreiter, Peter F. Stadler, Kay Prüfer, David Lambert, Janet Kelso, Torsten Schöneberg

**Affiliations:** Institute of Biochemistry, Medical Faculty, University of Leipzig, Johannisallee 30, Leipzig, 04103 Germany; Department of Evolutionary Genetics, Max Planck Institute for Evolutionary Anthropology, Leipzig, 04103 Germany; Department of Neuroscience, Unit of Functional Pharmacology, Uppsala University, Box 593, Husargatan 3, Uppsala, 751 24 Sweden; Griffith School of Environment and School of Biomolecular and Physical Sciences, Griffith University, Nathan, Queensland 4111 Australia; Department of Computer Science, and Interdisciplinary Center for Bioinformatics, University of Leipzig, Leipzig, 04103 Germany; Department of Genetics and Evolutionary Biology, University of São Paulo, São Paulo, SP 05508-090 Brazil; Adaptive Evolutionary Genomics, Institute for Biochemistry and Biology, University Potsdam, Potsdam, 14469 Germany

## Abstract

**Background:**

Kiwi, comprising five species from the genus *Apteryx*, are endangered, ground-dwelling bird species endemic to New Zealand. They are the smallest and only nocturnal representatives of the ratites. The timing of kiwi adaptation to a nocturnal niche and the genomic innovations, which shaped sensory systems and morphology to allow this adaptation, are not yet fully understood.

**Results:**

We sequenced and assembled the brown kiwi genome to 150-fold coverage and annotated the genome using kiwi transcript data and non-redundant protein information from multiple bird species. We identified evolutionary sequence changes that underlie adaptation to nocturnality and estimated the onset time of these adaptations. Several opsin genes involved in color vision are inactivated in the kiwi. We date this inactivation to the Oligocene epoch, likely after the arrival of the ancestor of modern kiwi in New Zealand. Genome comparisons between kiwi and representatives of ratites, *Galloanserae*, and *Neoaves*, including nocturnal and song birds, show diversification of kiwi’s odorant receptors repertoire, which may reflect an increased reliance on olfaction rather than sight during foraging. Further, there is an enrichment of genes influencing mitochondrial function and energy expenditure among genes that are rapidly evolving specifically on the kiwi branch, which may also be linked to its nocturnal lifestyle.

**Conclusions:**

The genomic changes in kiwi vision and olfaction are consistent with changes that are hypothesized to occur during adaptation to nocturnal lifestyle in mammals. The kiwi genome provides a valuable genomic resource for future genome-wide comparative analyses to other extinct and extant diurnal ratites.

**Electronic supplementary material:**

The online version of this article (doi:10.1186/s13059-015-0711-4) contains supplementary material, which is available to authorized users.

## Background

New Zealand’s geographic isolation, after the separation from Gondwana around 80 million years ago, provides an unequaled opportunity to study the results of evolutionary processes following geographic isolation. In New Zealand, the ecological niches typically occupied by mammals in most other parts of the world are dominated by birds. Kiwi (genus *Apteryx*), the national symbol of New Zealand, belong to a group of flightless birds, the ratites. This group is geographically broadly distributed including both extant members, which are the ostrich in Africa, the emu in Australia, the cassowary in New Guinea, and the rhea in South America, and, as extinct members, the moa from New Zealand and the elephant birds from Madagascar. New Zealand is thus the only landmass to have been inhabited by two ratite lineages. Strikingly, the two lineages are highly divergent in size with moa having a body size of up to 3 m [1] while kiwi, the smallest of the ratites, reaches only the size of a chicken. Moreover, while moa occupied the diurnal niche, kiwi are the only ratites, and one of only a few bird lineages (less than 3 % of the bird species [2]), that are nocturnal. Although the kiwi eye is unusually small for a nocturnal bird, it has a nocturnal-type retina [3]. This may indicate that the nocturnal adaptation of kiwi is recent, or alternatively, that changes in eye size are not a prerequisite for nocturnality.

We have sequenced and assembled the genome of *Apteryx mantelli*, the North Island brown kiwi, to improve our understanding of how genomic features evolve during adaptation to nocturnality and the ground-dwelling niche. We have also sequenced the transcriptome from embryonic tissue to provide support for the genome annotation. We identified genomic changes in kiwi that affect physiological functions, including vision and olfaction, which have been predicted to characterize nocturnal adaptation in the early history of mammals [4].

## Results

### Genome sequencing, assembly, and annotation

We prepared 11 libraries with several insert sizes from *Apteryx mantelli* genomic DNA and sequenced 83 billion base pairs (Gb) from small insert-size libraries and 120 Gb from large-insert mate-pair Illumina libraries (Additional file 1: Table S1). After read correction [5] we assembled contigs and scaffolds using SOAPdenovo [6] (Additional file 1: Note: Filtering and read correction; Genome assembly) to generate a draft assembly, which spanned 1.595 Gb (Additional file 1: Tables S2 and S3). The N50s of contigs and scaffolds were 16.48 kb and 3.95 Mb, respectively (Additional file 1: Table S3). Since the size of the kiwi genome is unknown, we estimated average coverage using a *19*-*mer* frequency distribution (Additional file 1: Figure S1) which yielded a genome size estimate of 1.65 Gb, placing the kiwi among the largest bird genomes sequenced to date [7] (Table 1; Additional file 1: Table S4). The assembled contigs and scaffolds cover approximately 96 % of the complete genome with an average sequence coverage of 35.85-fold after correction (Additional file 1: Note: Filtering and read correction). Assembly quality was assessed by chaining the kiwi scaffolds to two Sanger-sequenced bird genomes: chicken [8] and zebra finch [9]. A total of 50.09 % (0.8 Gb) of the kiwi genome is alignable in syntenic chains to 79.67 % of the much smaller chicken genome (1.07 Gb). A similar fraction, 57.61 % (0.9 Gb), of the kiwi sequence was alignable to 76.92 % of the zebra finch genome (1.2 Gb) (Additional file 1: Table S5). For comparison, 69.86 % (0.84 Gb) of the zebra finch genome is syntenically alignable to 83.51 % of the chicken genome. However, 91.96 % of the zebra finch sequences that are syntenic-chain-alignable to chicken showed conserved synteny in kiwi, suggesting that the kiwi genome assembly includes the majority of conserved regions between birds.

**Table 1:** Kiwi genome assembly characteristics and genomic features compared with other avian genomes (see Additional file 1: Table S4)

Species	Size of assembly (Gb)	N50 scaffolds (Mb)	Heterozygous SNP rate per kb
*Apteryx mantelli*	1.59	4	1.5
*Falco cherrug* [17]	1.18	4.2	0.8
*Falco peregrinus* [17]	1.17	3.9	0.7
*Taeniopygia guttata* [9]	1.2	10.4	1.4
*Ficedula albicolis* [90]	1.13	7.3	3.03
*Anas platyrhynchos* [18]	1.1	1.2	2.61
*Gallus gallus* [8]	1.07	15.5	4.5
*Meleagris gallopavo* [91]	0.93	1.5	~1.36

We identified a set of 27,876 genes following *de novo* gene prediction on the assembled genome (Additional file 1: Note: *De novo* gene prediction and gene annotation). To refine these gene annotations we used 47.5 Gb of transcript sequence data from kiwi embryonic tissue together with the *de novo* gene predictions and protein evidence from three well-annotated bird species (*G. gallus*, *T. guttata*, *M. gallopavo*) as input to the MAKER genome annotation pipeline [10]. A validated set of 18,033 genes was selected based on their alignment to orthologous genes in other birds and on supporting evidence provided by kiwi transcript sequences. In total, the gene models spanned 306.62 Mb of the assembly, with exons accounting for 23.96 Mb (approximately 1.6 %) of the total kiwi genome.

### Evolution of gene families

**Fig. 1:**
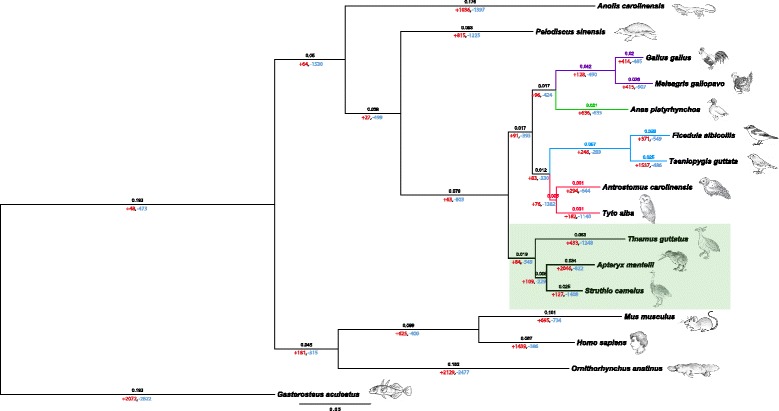
Phylogenetic tree of 16 species built on 623 TreeFam [12] single-gene families. Branch lengths are scaled to estimate divergence times. All branches are supported by 100 bootstraps. The song bird clade is depicted in blue, *Galliformes* jn purple, *Anseriformes* in green, and nocturnal birds in red. Ratites (*Struthio camelus* and *Apteryx mantelli*) and *Tinamus guttatus* are highlighted in light green. The number of genes gained (+ red) and lost (− blue) is given underneath each branch. The rate of gene gain and loss for the clades derived from the most common recent ancestor was estimated [77] to 0.0007 per gene per million years

Gene family expansion and/or contraction have been proposed as important mechanisms underlying adaptation [11]. We explored patterns of protein family expansions and contractions in kiwi and used TreeFam [12] to define gene families in the kiwi and all bird and reptile genomes in Ensembl 73, as well as two nocturnal birds (barn owl, chuck-will’s-widow), two other ratites (ostrich, tinamou) [7] (GigaDB [13]), two mammals (human, mouse), and one fish (stickleback) (Ensembl 73 [14]). In total we identified 10,096 gene families shared between the inferred ancestral state and the 16 species considered, of which 623 represent single-gene families. For these single-gene families we constructed a maximum-likelihood phylogeny [15] (Fig. 1) and tested for changes in ortholog cluster sizes. In accordance with previous estimates, our results indicate a net gene loss on the avian branch [16].

Changes of gene-family sizes have been inferred for multiple *de novo* assembled genomes [17, 18]. However, many of these genomes have rather fragmented assemblies (Table 1); thus, results should be interpreted cautiously, only after manual inspection and ideally independent experimental confirmation.

We therefore manually examined the 130 gene families that had either significant expansion or contraction specifically to the kiwi branch. After excluding expansions that were caused by fragmentation of the assembly [19] only 85 gene families remained significant (Additional file 1: Table S6). Of these, 63 gene families are expanded in the kiwi. An analysis of gene family functions [20] showing expansion in kiwi identified enrichment in categories including signal transduction, calcium homeostasis, and motor activity (FDR <0.0001, Additional file 1: Figure S2A). Among the gene families that show contraction on the kiwi branch we found an enrichment of development-related Gene Ontology (GO) categories (FDR <0.0001, Additional file 1: Figure S2B).

Diversification of tetrapods and the colonization of terrestrial habitats are often accompanied by changes of physiological systems specifically in cellular signal transduction [21]. Membrane proteins are involved in cellular signaling, hence we aimed to determine more specifically which classes of membrane-expressed proteins have undergone changes in the number of coding genes. To this end we annotated the membrane proteome in kiwi, human, all birds, and reptiles present in Ensembl 74, two additional ratites (ostrich and tinamou) and two nocturnal birds (chuck-will’s-widow and barn owl) (Additional file 1: Note: Detection and classification of the membrane proteome; Additional file 1: Table S7). We manually inspected the classes which showed expansion in kiwi, to ensure that the higher number of predicted genes is not a result of assembly fragmentation. We found a significant expansion in kiwi of genes coding for adhesion and immune-related proteins (Additional file 1: Table S7). Additionally, we found a significant expansion of the Ephrin kinases class, which are functionally involved in the development of the sensory-motor innervation of the limb [22] and later on in tendons condensation and developing feather buds [23].

### Patterns of natural selection

To determine whether any branch-specific selection is present in kiwi we estimated branch ω-values (Ka/Ks substitution ratios) for 4,152 orthologous genes in eight bird species: kiwi, ostrich, tinamou, chuck-will’s-widow, barn owl, chicken, zebra finch, and turkey using CODEML [24]. Ortholog assignment was based on the orthology relation among chicken, zebra finch, and turkey defined in Ensembl 73 (Additional file 1: Note: Orthologs and Ka/Ks calculation). The kiwi average ω across all the orthologs is comparable to that in ostrich, and higher than in tinamou and night birds (0.291, 0.313, 0.145, 0.202, and 0.200 for kiwi, ostrich, tinamou, chuck-will’s-widow, and barn owl, respectively). This implies a relatively faster overall rate of functional evolution in kiwi and ostrich.

In addition to gene-family expansions/contractions, we used evidence of branch-specific selection to identify genes and functional pathways that may underlie kiwi-specific adaptations. For the 4,152 orthologous genes in the eight bird species we used the branch models from CODEML to perform likelihood ratio tests [24], comparing a simple model of one ω for all sites and branches versus a model where kiwi is defined as the foreground branch and the other birds as background. We first considered genes with a significantly higher ω on the kiwi branch than that in all other birds (LRT >3.84, significance at 5 %, 1 degree of freedom). Functional enrichment using GO [20] categories was tested using a hypergeometric test (Additional file 1: Note: Gene ontology and rapidly evolving genes). The same test was performed on genes evolving significantly slower in kiwi. To assign functional categories as either kiwi-specific, or shared with other ratites or nocturnal birds, a similar procedure was performed for each species of *Palaeognathae* (ostrich, tinamou) and night birds (chuck-will’s-widow, barn owl) by assigning each in turn as the foreground branch in CODEML.

After multiple testing correction using family-wise error rate none of the categories remained significant. For further analysis we considered only GO categories that had (1) a *P* value <0.05; (2) at least three significantly changed genes; and (3) the number of significant genes was at least 5 % of the total genes annotated in the GO category. GO categories that were over-represented (*P* value <0.05) on the kiwi branch, but not present in any of the other considered species, were identified as potentially kiwi-specific changes (Additional file 1: Note: Gene ontology and rapidly evolving genes). Notably, faster-evolving categories present in kiwi, but absent in any of the other species, are related to mitochondrion, feeding behavior and energy reserve metabolic process, visual perception, and eye photoreceptor cell differentiation (Additional file 1: Table S8A). Sensory perception of light stimulus is a faster evolving category shared, surprisingly, with the ostrich (Additional file 1: Table S8B). Among slower evolving categories, the mitochondrial outer membrane was one of the kiwi-specific categories (Additional file 1: Table S9A), while anion channel activity was a shared category with chuck-will’s-widow (Additional file 1: Table S9B). For the potentially biological meaningful categories which could explain kiwi-specific physiology we extracted the genes clustering in the node. GO categories have a high potential to deliver false-positive enrichment, which could be considered biologically meaningful a posteriori [25]. Therefore, future studies need to verify the adaptive functionality of genes belonging to the respective category (Additional file 1: Tables S8C and S9C).

It has been proposed that, in a nocturnal environment, genes involved in circadian rhythm have been under selective pressure [4]. Our species-specific selection screens did not identify circadian rhythm-related categories to be enriched for changed genes in either kiwi or the other nocturnal birds. However, since mutations in even a single gene may be relevant, we analyzed more closely biorhythm regulators from the neuropsin gene family. Encephalopsin (*OPN3*), melanopsin (*OPN4-1*), and neuropsin (*OPN5*) showed a similar ω in kiwi and the other branches and no obvious alterations could be detected in the sequence (Table 2). Similar to chicken [26], kiwi and the other tested birds have a duplication of the melanopsin gene (*OPN4-2*), which displayed significant signals of positive selection in kiwi but not in the other nocturnal birds. However, a branch-site selection analysis of this gene did not show any significant positively selected sites (Additional file 1: Note: Vision analysis).

**Table 2:** Annotated opsins in the *Apteryx mantelli* genome

*AptMant0* annotation ID	External gene ID	Description	ω background	ω *Apt. mantelli*	LRT
augustus_masked-scaffold541-abinit-gene-7.0-mRNA-1	*RHO*	No obvious alteration	0.044	0.14913	6.128*
augustus_masked-scaffold1311-abinit-gene-0.1-mRNA-1	*OPN1LW*	Partial sequence TM7	0.15601	0.59702	1.503
maker-scaffold728-augustus-gene-1.2-mRNA-1	*OPN1MW*	Deleterious mutation Glu^3.49^Lys	0.02093	0.26785	44.951*
augustus_masked-scaffold1068-abinit-gene-0.2-mRNA-1	*OPN1SW*†	Partial sequence, deleterious mutation Glu^6.30^Gly	0.03815	0.19244	5.162*
augustus_masked-scaffold9587-abinit-gene-0.0-mRNA-1	*SWS2*††	Partial sequence	0.02045	0.0001	0.514
maker-scaffold19-augustus-gene-28.1-mRNA-1	*OPN3*	No obvious alteration	0.10965	0.54221	3.211
augustus_masked-scaffold39-abinit-gene-55.0-mRNA-1	*OPN4-1*	No obvious alteration	0.14205	0.23127	2.733
augustus_masked-scaffold122-abinit-gene-6.0-mRNA-1	*OPN4-2*	No obvious alteration	0.18597	2.57434	8.194*
maker-scaffold597-augustus-gene-1.2-mRNA-1	*OPN5*	No obvious alteration	0.07114	0.0001	1.733
augustus_masked-scaffold1987-abinit-gene-3.0-mRNA-1	*opsin-VA-like*	No obvious alteration	0.31735	0.26196	0.035

LRT = likelihood ratio testing with one degree of freedom, between the null model (model = 0) and a model where the kiwi branch differs from other birds: chicken, turkey, zebra finch, chuck-will’s-widow, barn owl, tinamou, and ostrich (model = 2), implemented in CODEML from the PAML package [24]. Extended selection analysis in which nocturnal birds, ostrich, and tinamou are sequentially appointed as foreground branch are presented in Additional file 1: Table S10.

*P value <0.05

†Tested on orthologs in *Tinamus guttatus*, *Antrostomus carolinensis*, *Taeniopygia guttata*, *Gallus gallus*, and *Apteryx mantelli* (not present in *Struthio camelus* and *Tyto alba* assemblies)

††Tested on orthologs in *Chlamydera nuchalis*, *Chlamydera maculata*, *Sericulus chrysocephalus*, *Ptilonorhynchus violaceus*, *Scenopoeetes dentirostris*, *Ailuroedus crassirostris*, *Falco cherrug*, *Columba livia*, and *Apteryx mantelli*

### Kiwi sensory adaptations – vision

Nocturnality is accompanied by a number of specific changes, including adaptations in visual processing [4]. In contrast to most nocturnal animals, that have large eyes relative to their body size, kiwi have small eyes and reduced optic lobes in the brain [27]. However, the kiwi retina has a higher proportion of rods than cones which is consistent with adaptation to nocturnality [3]. Besides black/white vision mediated via rhodopsin (*RHO*), most birds have trichromatic or tetrachromatic vision, for which various additional opsins are responsible: *OPN1LW* (red), *OPN1MW* (green, *RH2*), *OPN1SW* (blue, subtypes *SWS1*, *SWS2*) [28]. We identified these genes in the kiwi assembly. The *RHO* gene in kiwi shows no interruption and no obvious function-impairing amino acid changes compared to other vertebrates. We were able to assemble only a partial sequence of the red opsin *OPN1LW* (transmembrane (TM) helix 7) and found no previously described deleterious amino acid changes within this region [29].

**Fig. 2:**
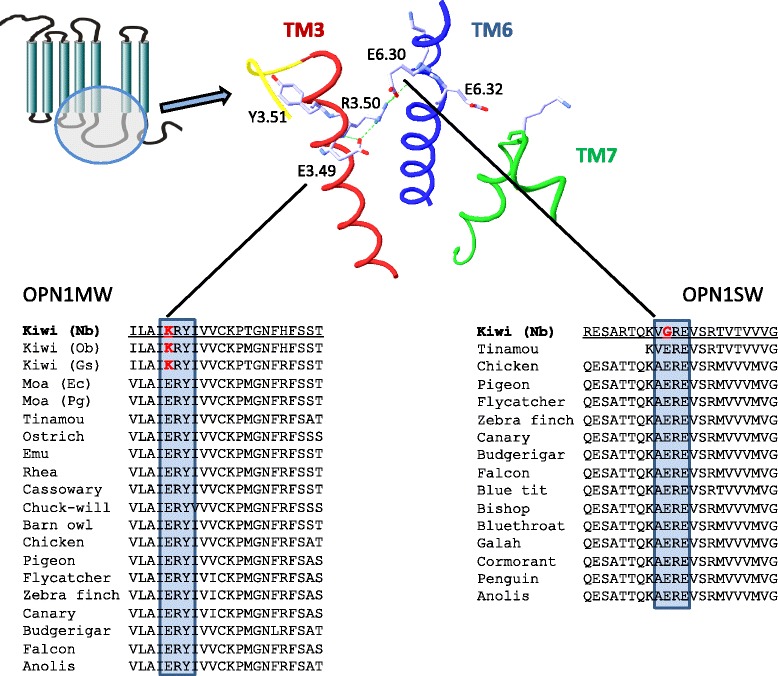
Protein sequence comparison revealed substitutions of Glu^3.49^ to Lys (E/DRY motif) and Glu^6.30^ to Gly in kiwi *OPN1MW* (*RH2*) and kiwi *OPN1SW*, respectively. Both residues are 100 % conserved in all birds sequenced so far and over 100 publicly available sequences of other vertebrate *OPN1MW* and *OPN1SW* orthologs. To assure the *OPN1MW*-change is kiwi-specific additional ratites were sequenced, including different kiwi species and the extinct moa. Glu^3.49^ of the E/DRY motif and Glu^6.30^ at the N-terminal end of helix 6 are parts of an ‘ionic lock’ interhelical hydrogen-bond network which is highly conserved in many rhodopsin-like GPCRs. Nb – North Island brown kiwi, Ob – Okarito brown kiwi, Gs – Great spotted kiwi, Ec – *Emeus crassus* (Eastern moa), Pg – *Pachyornis geranoides* (Mappin’s moa), Chuck-will – Chuck-will’s-widow

In the green opsin, *OPN1MW*, we identified a Glu^134^ to Lys substitution (relative position 3.49 in the Ballesteros and Weinstein nomenclature) in the highly conserved D/ERY motif of this rhodopsin-like GPCR. We confirmed this mutation in a second *Apteryx mantelli* individual, as well as in other kiwi species (Fig. 2). To determine whether the change is kiwi-specific we sequenced this domain of *OPN1MW* in other ratites, including the extinct moa. We found that Glu^3.49^ is 100 % conserved in all birds for which sequence was available and also in over 250 other vertebrate orthologs. Previous experimental analysis showed that mutation of Glu^3.49^ to Arg – another basic amino acid – results in a non-functional receptor protein [30]. Furthermore, the Asp or Glu in the D/ERY motif is also highly conserved in most other rhodopsin-like GPCRs and the identical mutation of Glu^3.49^ to Lys in the thromboxane A2 receptor, for example, prevents the receptor from being functionally expressed on the plasma membrane [31].

Similarly, at the N-terminal end of TM6 in *OPN1SW* we identified a highly conserved Glu^6.30^ which is present in all bird orthologs sequenced so far, except for kiwi *OPN1SW* where Glu^6.30^ is substituted by Gly. Previous functional characterization has shown that mutation of Glu^6.30^ destabilizes the H-bond network resulting in constitutively active opsins and other rhodopsin-like GPCRs [32, 33]. A constitutively active opsin is functionally incapable of light signal transmission [34] and is therefore non-functional.

Besides these two functionally well-characterized positions, we identified several other amino acids substitutions in kiwi *OPN1MW* and *OPN1SW*. Further, tests for branch and branch-site specific ω values for *OPN1MW* and *OPN1SW* on the kiwi branch showed no evidence for positively selected sites in kiwi (Additional file 1: Note: Vision analysis), suggesting that the greater ω values for kiwi are likely due to loss of constraint on these genes. Hence these genes are likely to be drifting and, considering the fact that only 8 % of all inactivating mutations in GPCRs are stop codons while almost 65 % are missense mutations [35–37], the described loss-of-function mutations in *OPN1MW* and *OPN1SW* render color vision of kiwi, unlike for other sequenced ratites (Fig. 2), absent – at least for the green and blue spectral ranges.

We tentatively dated the opsin-loss-of-function event as an indicator of the timing of adaptation to the nocturnal niche. Assuming that the loss of constraint happened on the kiwi branch in a short period of time and changed the rate of selection, measured by the ω value, from the average over bird lineages (0.021 for *OPN1MW* and 0.014 for *OPN1SW*, Table 2) to the neutral ω value of 1, the loss of function was dated to 30–38 million years ago (Additional file 1: Note: Vision analysis), which places the event shortly after the arrival of kiwi in New Zealand [38].

### Kiwi sensory adaptations – olfaction

Kiwi are unique among birds in having nostrils present at the end of their prominent beaks and have been reported to depend largely on tactile and olfactory senses for foraging [39]. To investigate whether the genome shows signs of olfactory adaptation in kiwi we assessed the numbers of olfactory receptor (OR) genes [40] and the diversity in the OR sequence [41].

The only previous approach to molecular characterization of the olfactory system in kiwi was based on PCR amplification of ORs with degenerate primers [42]. This allowed only a rough estimation of the number of ORs of 478 genes (95 % confidence interval 156–1,708 genes). PCR with degenerate primers only produces incomplete fragments of the genes and hence the accurate quantification of gene families with highly similar sequences, as in the case of ORs, is prone to over-estimation [43]. In contrast, *de novo* genome assembly facilitates a global assessment of the gene repertoire [44] and can therefore be used to provide a more accurate estimate of the OR repertoire. We thus annotated the OR genes in kiwi, as part of the entire membrane proteome, on the basis of putative functionality and seven transmembrane helices (7TM) (Additional file 1: Note: Olfactory receptor genes identification and annotation). The number of non-OR receptor families was comparable to other avian species, suggesting that the membrane proteome is well annotated in kiwi (Additional file 1: Table S7). This analysis revealed an initial set of 82 OR genes in the kiwi genome. However, ORs are highly duplicated across the genome and such regions could be prone to being overcollapsed during the assembly process. We therefore estimated the copy number of each annotated OR using a correction based on coverage. To obtain the correction factor for each OR, read-coverage in the OR region was divided by the genome-wide average coverage corresponding to its GC bin. Following this correction we estimated that up to 141 OR genes are present in the kiwi genome, of which 86 encode for full-length receptors while the rest are most likely pseudogenes due to frameshifts, premature stop codons, or truncations (Additional file 1: Note: Olfactory receptor genes identification and annotation). The estimated proportion of intact ORs among all OR genes in kiwi (61 %) is lower than previously reported for *Apteryx australis* [42] (78.6 %), but much higher than in zebra finch (38 %) [45].

Comparative analysis of the OR repertoire shows that the kiwi genome has both the α and the γ subgroups of type 1 OR genes, as reported for other bird genomes sequenced so far [45]. Unlike the majority of other birds analyzed so far, kiwi has a higher number of γ subgroup ORs. Gene family size estimates are highly dependent on genome quality [46] and continuous curation is ongoing even for well-annotated genomes: for example, in the chicken olfactory repertoire the number of annotated ORs changed by a factor of eight in two consecutive Ensembl releases (release 73 – 251 ORs and release 74 – 30 ORs). Further improvement of genome qualities, including kiwi, are therefore required for the identification of a complete set of ORs. Thus, a correlation between olfactory acuity and the number of ORs in different birds could be subject to error.

**Fig. 3:**
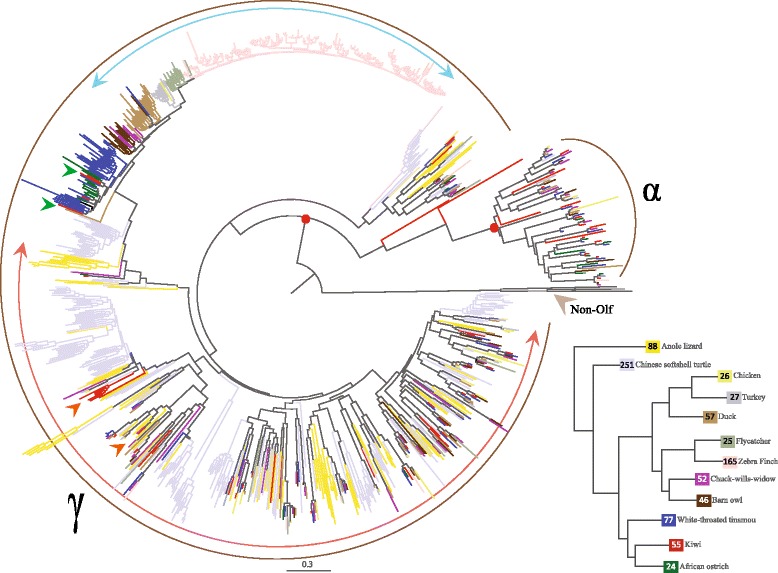
Maximum likelihood (ML) tree constructed using full-length intact α and γ group olfactory receptors from 10 birds (chicken, zebra finch, flycatcher, duck, turkey, chuck-will’s-widow, barn owl, ostrich, tinamou, and kiwi) and two reptile genomes (anole lizard and Chinese soft-shell turtle). The ML topology shown above was cross-verified using the neighbor joining (NJ) method. Three Class A (Rhodopsin) family GPCRs from chicken genome, dopamine receptor D1 (DRD1), dopamine receptor D2 (DRD2), and histamine receptor H1 (HRH1) were used as the out-group (shown as non-olfactory receptors). The red dot indicates confidence estimates (% bootstrap from 500 resamplings, >90 % bootstrap support from both ML and NJ methods) for the nodes that distinguish α and γ ORs. The scale bar represents the number of amino-acid substitutions per site. The topology supports lineage specific expansions of γ group olfactory genes in the bird and the reptile species. Note, a few of the γ group ORs in kiwi cluster with reptilian ORs (highlighted by orange arrowhead), while some cluster basal to the clade containing bird ORs (highlighted by green arrowhead). The topology supports contrasting evolutionary rates within the analyzed γ ORs, as indicated by short (blue arc with arrowheads) and long branch lengths (pale orange arc with arrowheads). The inset shows the number of intact olfactory receptors in each species that are analyzed using the ML tree topology

Phylogenetic comparison of OR repertoires suggest that γ ORs within bird and reptile genomes exhibit contrasting evolutionary rates. Tree topology suggests that γ ORs in a few birds and reptiles show species-specific clustering pattern (Fig. 3). This pattern was previously described in birds and it was suggested that these receptors have undergone adaptive evolution with respect to the occupied environmental niche [45]. However, a few γ ORs belonging to kiwi cluster with their reptilian counterparts, while some cluster basal to the clade containing most bird γ ORs (Fig. 3).

Phenotypic diversity in olfaction is, in part, attributable to genetic variation with a wider range of odors thought to be detectable given more genetic variation [41]. Since the absolute number of ORs might be a poor predictor of olfactory abilities, we investigated the variation in the γ ORs sequence as a measure of the range of possible detectable odors. The average protein sequence entropy was calculated to check for variation within the γ-c clade in each species (Additional file 1: Note: γ-c clade OR within-species protein sequence entropy).

Previous studies have shown that Shannon entropy (H) analysis is a sensitive tool for estimating the diversity of a system [47, 48]. For protein sequence, H ranges from 0 (only one residue is present at that position in the multiple sequence alignment) to 4.322 (all 20 residues are equally represented in that position). Typically H ≤2 is attributed to high conservation [49]. H values in birds were in the range of 0.34±0.05 (zebra finch) to 1.11±0.12 (chicken). The average entropy in kiwi sequences was 1.23±0.15, significantly higher than all other bird species investigated (*P* value = 0.003 Wilcoxon Signed-Rank test, Additional file 1: Note: γ-c clade OR within-species protein sequence entropy). We conclude that overall the γ-c clade of ORs are highly similar in sequence, in accordance with previously published data [45]. However, since detection of a wider range of odors is correlated to genetic variation of ORs [41], the significantly higher H in kiwi ORs is suggestive for a broad odor acuity in this species in comparison to other birds.

### Kiwi morphology

The most prominent phenotype of kiwi, lack of wings, has been linked to energy conservation [50] and to the limited resources in New Zealand in late Oligocene [51]. Like most ratites, kiwi are flightless, but the phylogenetic tree of *Palaeognathae* implies that this phenotype evolved several times independently in this order [38]. Unlike ostriches and rheas, that possess prominent wings, kiwi show only vestigial invisible wings, while moa lack even vestiges [52].

To determine whether we can identify the genetic basis for the extremely regressed wings in kiwi we annotated genes in the highly conserved signaling pathways related to limb development (Additional file 1: Note: Kiwi morphology analysis; Additional file 1: Figure S3). These include genes belonging to the *FGF*s, *TBX* cluster, *HOX* cluster (Additional file 1: Figure S4; Additional file 1: Table S11), *WNT*, *SALL*, and *FIBIN* genes, known to be responsible for limb and wing development [53] (Additional file 1: Table S12). Growth and transcription factors typically influence the development of both upper and lower limbs, while *FIBIN* is currently the only gene described to be exclusively involved in the development of the upper limb [53].

For these clusters of genes, we aligned corresponding orthologs and translated multiple alignments, which were then manually inspected. No insertions, deletions, and/or stop codons that would clearly disrupt the open reading frame could be identified in the inspected genes. Additionally, we found all 39 *HOX* genes expected for the Sauropsid ancestor [54] and investigation of regulatory sequences within the *HOX* clusters by phylogenetic footprinting showed no preferential loss of conserved DNA elements in *Apteryx mantelli* compared to *Galliformes* (Additional file 1: Figure S4; Additional file 1: Table S11).

To detect signs of different evolution in kiwi wing and tail developmental genes we performed a selective constraint analysis using the CODEML branch test (Additional file 1: Note: Selection analysis on limb development genes; Additional file 1: Table S12). Of these genes *FIBIN* was the only gene that showed signals of positive selection on the avian tree including chicken, turkey, and zebra finch (Additional file 1: Figure S5). Three sites with signs of positive selection that were 100 % conserved in the other species show a different amino acid in kiwi: exchanges of Ser^136^Ala, Gln^148^Arg, and Phe^162^Cys (positions are relative to the mouse *Fibin* coding sequence). The functional relevance of these substitutions is unclear and needs to be studied when experimental tests of FIBIN function become available.

Since no obvious alterations could be found in the coding sequences of genes involved in developmental processes, which could explain the regressed-wing morphology of kiwi, we further analyzed ultra-conserved non-coding elements (UCNEs) (Additional file 1: Note: Ultra-conserved non-coding elements analysis). UCNEs are defined as DNA non-coding regions of ≥95 % sequence identity between human and chicken, longer than 200 bp [55]. The majority of UCNEs cluster in genomic regions containing genes coding for transcription factors and developmental regulators [56] and experimental studies in transgenic animals have shown that some of these sequences can act as tissue-specific enhancers during developmental processes [57]. Of the 4,351 UCNEs annotated in UCNEbase [55], 19 showed more than the expected 5 % sequence variation as defined in the database [55] (Additional file 1: Table S13). Among these, four were related to *HOXA*, *TBX2*, *Sp8*, and *TFAP2A* genes which have been previously described in limb development pathways [53, 58, 59], suggesting that changes in non-coding elements could be involved in kiwi’s loss of wings.

## Discussion

With their small body size, extremely large egg size, nocturnal life style, and prominent nostrils at the end of their beaks, among several other traits, kiwi represent probably the most unusual member of the ratites [60]. A recent mitochondrial DNA phylogeny placed kiwi as the closest relatives of the extinct Madagascan elephant birds [38]. Whether dispersal or vicariance best describe ratite distribution has been debated for over a century [61]. A phylogeny including 169 bird species, built on 32 kb from 19 independent loci, showed ostrich as basal in the *Palaeognathae* clade [62]. In contrast, our phylogeny, based on 623 1:1 orthologs in 16 species, totaling approximately 700 kb, places the tinamou as basal to *Palaeognathae* with 100 % bootstrap confidence (Fig. 1; Additional file 1: Figure S6). However, when the phylogeny was constructed for 10 bird species using just UCNEs (totaling >1 Mb) the topology of the tree matches that obtained from fewer loci from a larger number of species which agrees with a previous publication [62] (Additional file 1: Figure S7). Including more ratites and a larger number of (hand-curated) loci should provide better resolution of the tree topology, and indeed the topology we obtain here is well-supported. However, we note that the topology changes depending on the gene sets that are included (Additional file 1: Figs. S6 and S7) and that when using ultra-conserved sequences the phylogeny differs from that obtained from a larger, more representative set of genes. Hence, future availability of additional genomes and ortholog sets from multiple ratites will allow a better understanding of their origin.

Nevertheless, a previous study has estimated that kiwi diverged from the Madagascan elephant birds about 50 million years ago [38] (Additional file 1: Figure S8). This estimate post-dates the split of Madagascar and New Zealand from Gondwana, which took place around 100 and 80 million years ago, respectively, and implies that ratites must have dispersed by flight and also that kiwi arrived on New Zealand less than 50 million years ago. This conclusion is supported by the fossil record in New Zealand, which includes a flighted kiwi ancestor [63]. At the time kiwi arrived, moa already inhabited New Zealand and it has been hypothesized that moa were monopolizing the diurnal ground niche, which forced kiwi to adapt to an alternative nocturnal lifestyle [38]. This would suggest that kiwi adapted to the nocturnal niche soon after arriving on the island. The loss of function that we observe in *OPN1SW* is indicative of adaptation to nocturnality [64]. We dated the loss of function in several color vision opsins to 30–38 million years ago, which is consistent with the arrival of the kiwi in New Zealand less than 50 million years ago, and their subsequent adaptation to a nocturnal niche.

In contrast to birds, which almost certainly have a diurnal origin, the nocturnal bottleneck hypothesis suggests that mammals were nocturnal for about 160 million years in their evolution as they were restricted to nighttime activity to avoid dinosaurs which were the dominant diurnal taxon at this time [4]. According to this hypothesis, several traits typical for mammals, including a well-developed sense of smell, limited color vision, increased eye size, and an energetic metabolism optimized for sun radiation-independent body temperature regulation, have been shaped by the nocturnal environment [65, 66]. Nocturnally adapted Mesozoic mammals also tended to have a small body size, an insectivorous diet, and low energy metabolism [67]. Interestingly, kiwi has the smallest body size among flightless ratites, the lowest metabolic rate among birds [68, 69], and an insectivorous diet, suggesting a pattern of evolution that is similar to the evolution of mammals under nocturnality. Consistent with this hypothesis, our genome-wide scans for patterns of positive selection showed enrichment in GO categories like mitochondrion functions and energy reserve metabolic process (Additional file 1: Table S8A), both related to metabolic rate. Moreover, we found strong evidence for a loss of color vision in kiwi and their retinal structure also clearly supports adaptation to vision under low light levels [3]. Although the small eye size of kiwi [27] is unusual for a nocturnal species, based on the retinal anatomy Corfield *et al.* rejected a regressive evolution model for kiwi vision and suggested that kiwi have an acuity in detecting low light levels similar to other nocturnal species [3]. This suggests that molecular mutations and retinal structure changed faster than eye size. In birds, eye size was described to scale to body mass with an exponent similar to brain mass and metabolic rate [70]. Thus, the low metabolic rate of kiwi [68] could be the constraint for their relatively small eyes. Alternatively, kiwi might serve as an example that adaptations in the retinal structure could be sufficient, and changes in eye size are not absolutely necessary. This conclusion may be supported by the absence of variation in eye shape according to activity pattern observed in lizards and non-primate mammals [71].

It has long been hypothesized that unlike most bird species kiwi is more similar to mammals in their reliance on olfactory and mechanical cues for foraging, perceived by the nostrils and mechanoreceptors located at the end of its bill, for foraging [72]. We found that the kiwi, unlike other ratites, has an increased diversity in the bird-specific γ-c clade ORs. Since OR diversity is hypothesized to correlate positively with olfactory acuity in vertebrates [42, 73], the significantly higher diversity in kiwi ORs compared to other birds (Additional file 1: Figure S9) suggests that kiwi may be able to distinguish a larger range of odors than other birds.

Steiger *et al.* formulated two possible scenarios that could explain γ ORs evolution in birds: the first hypotheses that species-specific γ ORs arose from independent expansion events in each species, while the second assumes that the ancient γ OR clade was more diverse and became homogenized by concerted evolution within species [45]. Some γ ORs of kiwi, ostrich, tinamou, and nocturnal birds clustered with their reptilian counterparts, while others clustered basal to the clade containing most bird γ ORs (Fig. 3). This supports a two-fold conclusion: (1) γ ORs in kiwi are more diverse in sequence than in other birds investigated, which was verified by the significantly higher sequence entropy; and (2) since kiwi is basal to the *Neognathae* (Fig. 1), the ancestral state of γ OR clade is probably diversified compared to other modern birds.

## Conclusions

Since its arrival in New Zealand sometime after 50 million years ago, the kiwi adapted to a nocturnal, ground-dwelling niche. The onset of adaptation to nocturnality appears to have been approximately 30–38 million years ago, about one-fifth of the time proposed for the evolution of mammals in a nocturnal environment. The molecular changes present in the kiwi genome are in accordance with the adaptations that are hypothesized to have occurred during early mammalian adaptation to nocturnality. This suggests similar patterns of adaptation to the nocturnal niche both in kiwi and mammals. Further comparative analyses, including other diurnal *Palaeognathae*, as well as additional nocturnal bird groups and their diurnal sister species, should shed further light on the genomic imprints of adaptation to a nocturnal life style.

## Methods and materials

### Genome sequence assembly and annotation

We sequenced *Apteryx mantelli* female individuals, which originate from the far North (kiwi code 73) and central part – Lake Waikaremoana (kiwi code AT5 and kiwi code 16–12) of North Island (Additional file 1: Figure S10). They were sampled in 1986 (kiwi code 73) and 1997 (kiwi code AT5 and 16–12) in ‘operation nest egg’ carried out by Rainbow and Fairy Springs, Rotorua. No animals were killed or captured as a result of this study and genome assembly was performed with iwi approval from the Te Parawhau and Waikaremoana Māori Elders Trust.

We extracted genomic DNA from *Apteryx mantelli* embryos. Libraries with insert sizes of 240 bp, 420 bp, 800 bp, 2 kb, 3 kb, and 4 kb were obtained from individual kiwi code 73, and mate-paired-end libraries 7 kb, 9 kb, 11 kb, and 13 kb, from individual kiwi code 16–12. DNA from individual AT5 was used to build a 350 bp insert-size library with the purpose of confirming kiwi-specific sequence polymorphisms and was not included in the genome assembly (Additional file 1: Note: Sampling, DNA library preparation and sequencing; Additional file 1: Table S1). Paired-end sequencing was performed on HiScanSQ and HiSeq platforms with read lengths of 101 bp and 96 bp, respectively.

Sequencing errors were corrected using Quake [5] (Additional file 1: Note: Filtering and read correction; Additional file 1: Figure S1). A total of 52.53 Gb of high-quality sequence was used for *de novo* assembly with SOAPdenovo [6]. The short-insert-size libraries (240 bp, 420 bp, 800 bp) were used to build contigs. Based on paired-end information scaffolds were generated using all libraries (2 kb, 3 kb, 4 kb, 7 kb, 9 kb, 11 kb, 13 kb). Remaining gaps in the scaffolds were closed using the paired-end information (Additional file 1: Note: Genome assembly). This final assembly (*AptMant0*) was used for all subsequent analyses.

Gene annotation was performed with the MAKER pipeline [10], using several sources of evidence: *de novo* gene predictions, RNA-Seq data, and protein evidence from three species (*G. gallus*, *T. guttata*, and *M. gallopavo*) (Ensembl version 72). Briefly, after repeat masking, gene models were predicted by Augustus version 2.7 [74] using the training dataset for chicken. *Apteryx mantelli* RNA-Seq data were then aligned to *AptMant0* using NCBI BLASTN version 2.2.27+ [75] and BLASTX was used to align protein sequences to identify regions of homology. Finally, using both the *ab initio* and evidence-informed gene predictions, Maker updated features such as 5’ and 3’ UTRs based on RNA-Seq evidence and a consensus gene set was retrieved (Additional file 1: Note: *De novo* gene prediction and gene annotation).

### Comparative genome analysis

Triplet orthologs between chicken, zebra finch, and turkey were downloaded from Ensembl 73. Kiwi genes were considered orthologs to a triplet if the ortholog assignment from Maker agreed with the orthologous gene assigned in each of the three considered species. The ostrich, tinamou, chuck-will’s-widow, and barn owl orthologs were assigned by orthology to the chicken proteins. After assigning orthology in the eight avian species, coding sequences were aligned and two different sets of alignments were compiled for further analysis:

Set 1: alignments of all eight species that do not contain a single frameshift indel.

Set 2: the longest uninterrupted run of at least 200 aligned bases in each multiple sequence alignment, for which we first ensured that gaps in the alignment were not introduced by unresolved bases in our assembly.

The CODEML program from the package PAML [24] was run first on four avian lineages: *G. gallus*, *T. guttata*, *M. gallopavo*, and *A. mantelli* to compare the kiwi genome to high-quality annotated ones. Six pairwise combinations were run to obtain estimates of non-synonymous (Ka) and synonymous (Ks) changes in the four avian lineages. Ka and Ks distributions were compared pairwise between all four avian species on a set of 3,754 orthologous genes which presented no frameshifts or indels (Additional file 1: Figure S11).

We next scanned for differently evolving genes with the CODEML program under a branch model (model = 2, two ωs for foreground and background branches, respectively, vs. model = 0, one ω for all branches, compared via likelihood ratio test) [24] using the set of orthologs as defined above in the eight bird species (Additional file 1: Note: Orthologs and Ka/Ks calculation).

Branch specific ω values were used to identify GO categories that are evolving significantly different on each of the following bird species: kiwi, ostrich, tinamou, barn owl, and chuck-will’s-widow. GO categories enrichment was tested using the FUNC [76] package.

A hypergeometric test was run for each species separately on genes having a significantly higher ω. Multiple testing correction was done using family-wise error rate. Categories with *P* value <0.05 were considered for further analysis if at least three significantly changed genes were present in the GO category, and the number of significant genes was greater or equal to 5 % of the total genes annotated in the respective GO category. The same test was applied on genes with a significantly smaller ω in each of the species. Kiwi-specific categories were considered those which showed no enrichment in any of the other ratites or night birds (Additional file 1: Note: Gene Ontology and rapidly evolving genes).

We used the TreeFam methodology to define gene families [12] across 16 genomes: *Gallus gallus*, *Anas platyrhynchos*, *Ficedula albicollis*, *Meleagris gallopavo*, *Taeniopygia guttata*, *Pelodiscus sinensis*, *Anolis carolinensis*, *Homo sapiens*, *Mus musculus*, *Gasterosteus aculeatus*, *Ornithorhynchus anatinus*, downloaded from Ensembl 73 [14], *Tinamus guttatus*, *Struthio camelus*, *Antrostomus carolinensis*, *Tyto alba*, downloaded from GigaDB [13], and *Apteryx mantelli*. The longest transcript was chosen for further analysis. For the single-copy orthologous families, genes were aligned against each other. To build a consensus phylogenetic tree (Fig. 1) the resulting alignments were loaded in PAUP* [15] version 4.0d105 and trees were inferred using maximum likelihood, with default parameters. To measure the confidence for certain subtrees, a series of 100 bootstrap replicates were performed (Additional file 1: Note: Nuclear loci phylogeny).

We determined the branch-specific expansion and contraction of the orthologous protein families among the 16 species using CAFE (computational analysis of gene family evolution) version 3.0 [77] with lambda option of 0.0007 (Additional file 1: Note: Gene families evolution using CAFE). Pfam IDs corresponding to the TreeFam families were assigned to GO categories. We tested whether significant (*P* <0.05) contraction/expansion events cluster in different GO categories using ClueGO with a hypergeometric test [78] (Additional file 1: Figure S2).

### Membrane proteome annotation

Complete protein sequence sets for the following bird and reptile species were downloaded from Ensembl 74 [14]: *Taeniopygia guttata*, *Meleagris gallopavo*, *Ficedula albicollis*, *Anas platyrhynchos*, *Pelodiscus sinensis*, *Gallus gallus*, and *Anolis carolinensis. Homo sapiens* from the same Ensembl version was used as outgroup. Protein sequences of ratites (*Tinamus guttatus*, *Struthio camelus*) and nocturnal birds (*Antrostomus carolinensis*, *Tyto alba*) were downloaded from GigaDB [13]; although these genomes are more fragmented than the ones from Ensembl, annotation of the membrane proteome in birds adapted, like kiwi, to the nocturnal niche and the ones belonging to the same clade as kiwi, allows to differentiate between events that are clade-specific or shaped by nocturnality. Only the longest protein sequence for each gene was considered for analysis. Membrane proteins and signal peptides were predicted for all species with Phobius [79]. These proteins were classified based on a manually curated human membrane proteome dataset, which describes family relationship and molecular function. The predicted membrane proteins were aligned to the human membrane proteome dataset with the BLASTP program of the BLAST package using default settings (v. 2.2.27+) [75]. Each predicted membrane protein was classified according to its best human hit with an e-value <10^−6^. Predicted membrane proteins with no hit were deemed unclassified, along with those proteins that hit an unclassified human protein (Additional file 1: Note: Detection and classification of the membrane proteome; Additional file 1: Table S7).

### Vision evolutionary analysis

Opsins are G protein-coupled receptors known to play a role in light signal transduction and night-day cycle (Table 2). For these genes ω was estimated by appointing sequentially kiwi, ostrich, tinamou, chuck-will’s-widow, and barn owl as the foreground branch under the CODEML branch model (model = 2) [24] as described for comparative genome analysis. Inactivating mutations were verified by checking that they were present in reads from both sequenced individuals and in other kiwi species, by Sanger sequencing (*OPN1MW*) (Fig. 2; Additional file 1: Note: Vision analysis).

### Olfaction evolutionary analysis

Olfactory receptors (ORs) in kiwi were annotated using both the Augustus *de novo* gene prediction and the Maker information after scaffold positions were checked and redundant sequences were removed.

We then performed four steps (Additional file 1: Figure S12):

i. Functional ORs from chicken [45] were downloaded and aligned against the kiwi transcriptome using TblastN with default parameters. After collecting overall hits for each query (every chicken OR served as query), identical (same) hits from each run were removed to obtain a non-redundant dataset.
ii. A Pfam search against the kiwi proteome with a default e-value cutoff of 1.0 was used to identify sequences that contained 7tm_4 domain (olfactory domain).
iii. The 7tm_4 domain was searched against the kiwi proteome by a CDD search (conserved domain database search).
iv. Separate HMM profiles were built from conserved 7tm regions of functional ORs of chicken, turkey, and zebra finch obtained from previous studies [45]. Using the three HMM profiles, HMM searches were performed against the kiwi proteome and non-redundant hits were retrieved from combined results of all three searches.

A CD-HIT (Cluster Database at High Identity with Tolerance) was performed to remove identical sequences with a cutoff of 100 %. Preliminary phylogenetic analysis was performed using a maximum likelihood approach (Additional file 1: Note: Olfactory receptor genes identification and annotation). Non-ORs were removed if they clustered separately from ORs. We excluded pseudogene candidates if at least one premature stop codon and/or frameshifts could be identified in the kiwi sequence.

OR repertoire estimates were curated based on genomic coverage calculated using samtools mpileup version 0.1.18 [80] on the alignment of the 240 bp, 420 bp, 800 bp insert-size libraries to *AptMant0* (Additional file 1: Note: Olfactory receptor genes identification and annotation). The correction factor for each annotated OR was obtained by dividing the read coverage in that region to the GC-content corresponding average coverage over the entire genome. For example, if an OR sequence had a GC content of 50 %, we calculated the average genome-wide coverage corresponding to the GC bin of 50 % to be 35-fold (Additional file 1: Note: Genome coverage and estimation of genome size; Additional file 1: Figure S13). Given a coverage in the respective OR region of 105-fold, we obtained a correction factor of 3 after dividing the OR sequence coverage (that is, 105-fold) by the GC-bin corresponding coverage (that is, 35-fold). The final number of estimated ORs was obtained by multiplying the number of initially annotated genes with their corresponding correction factors.

Using the same annotation procedure, the OR gene repertoire was estimated in all bird and reptile genomes from Ensembl 74, two nocturnal birds (chuck-will’s-widow and barn owl) and two *Palaeognathae* (ostrich and tinamou) for comparative phylogenetic analysis with the kiwi OR dataset. All obtained OR genes were then aligned using MAFFT [81] v7, with BLOSUM62 as the scoring matrix and default settings of option E-INS-I. Phylogenetic analyses were run using both maximum likelihood (ML) and neighbor joining (NJ) methods (Additional file 1: Note: Comparative phylogenetic analysis on ORs from kiwi and other bird and reptile genomes). The reliability of the phylogenetic trees was evaluated with 500 bootstrap replicates.

We calculated Shannon entropy (H) using within species multiple sequence alignments of γ ORs for all birds and reptiles genomes separately with a built-in function from BioEdit [82] (Additional file 1: Note: γ-c clade OR within-species protein sequence entropy).

### Kiwi morphology

Previously characterized wing development genes [53] were assigned orthologs in kiwi, chicken, zebra finch, and turkey (Additional file 1: Figure S3; Additional file 1: Table S12). We aligned the sequences and multiple alignments were translated and manually inspected for sequence differences as well as insertions/deletions and rearrangements. We examined selective pressures under the branch models implemented in CODEML [24]. The one-ratio model (model = 0, NSsites = 0) was used to estimate the same ω ratio for all branches in the phylogeny. Then, the two-ratio model (model = 2, NSsites = 0), with a background ω ratio and a different ω on the kiwi branch, was used to detect selective pressure acting specifically on the kiwi branch. These two models were compared via a LRT (1 degree of freedom), as mentioned above [83].

Scaffolds and isolated contigs harboring (putative) *HOX* genes were identified by BLAST and mapped to all 673 sauropsid HOX protein sequences from GenBank. Translated *HOX* sequences of *Apteryx* were aligned to the HOX proteins extracted from Genbank and differences were identified by manual inspection. Potential regulatory sequences in the HOX cluster region were identified by phylogenetic footprinting using tracker2 [84] (Additional file 1: Figure S4).

To retrieve the entire coding region of the *FIBIN* gene in kiwi, we designed primers based on the chicken and ostrich sequence (Additional file 1: Table S14). Using the 276-bp fragment amplified by Sanger sequencing, we blasted transcriptome sequences from kiwi and iteratively assembled the entire coding sequence. Since *FIBIN* showed signs of positive selection in the preliminary analysis as described above, extended selection analysis was performed using 15 species: human, mouse, bat, whale, dolphin, turtle, lizard, python, flycatcher, chicken, zebra finch, frog, zebrafish, and pufferfish (Additional file 1: Note: Fibin identification and selection analysis; Additional file 1: Figure S5). The branch-site tests were used to detect signals of selective pressure on each branch (NSsites = 2, model = 2, compared to the same model but with omega fixed to 1, via LRT). Amino acid changes with signs of selection and specific for the kiwi were visualized in both sequenced individuals.

Chicken UCNEs annotations were downloaded from the ultra-conserved non-coding element UCNEbase [55]. Orthologous regions in *Apteryx mantelli* and *Struthio camelus*, *Tinamus guttatus*, *Tyto alba*, *Antrostomus carolinensis* genomes, downloaded from GigaDB [13], and birds from Ensembl 74 [14] *Ficedula albicollis*, *Taeniopygia guttata*, *Anas platyrhynchos*, and *Meleagris gallopavo* were established using Blast 2.2.25 [85] with ‘blastn’ and default parameters. *Gallus gallus* genome Ensembl 74 was used as control in the orthology assignment. Orthologous regions from each of the species were aligned [86] to the reference UCNE and the number of mismatches between the UCNE and the target genomes were determined (Additional file 1: Note: Ultra-conserved non-coding elements analysis).

### Data availability

Assembly, raw DNA, and RNA sequencing reads have been deposited in the European Nucleotide Archive under the BioProject with accession number: PRJEB6383.

HOX Cluster annotation files were deposited on [87] and [88].

UCNEs multiple fasta files and analysis have been deposited on [89].

The kiwi *FIBIN* sequence was deposited in GenBank under BankIt 1821198 FIBIN KR364000.

## Additional file

Additional file 1: **Supplementary Material contains Supplementary Figs. S1–S15, Supplementary Tables S1–S17, Supplementary Note, and Supplementary References.**

## Abbreviations

bp: base pair
CDD: Conserved domain database
CD-HIT: Cluster database at high identity with tolerance
Gb: Giga base pairs
GO: Gene ontology
GPCR: G protein-coupled receptor
H: Shannon entropy
HMM: Hiden markov model
kb: kilo base pairs
LRT: Likelihood ratio test
Mb: Mega base pairs
ML: Maximum likelihood
NJ: Neighbor joining
OR: Olfactory receptor
PCR: Polymerase chain reaction
TM: Transmembrane
UCNE: Ultra-conserved non-coding element
