# Supplementary material for: Kiwi genome provides insights into evolution of a nocturnal lifestyle
Source: Genome Biol. 2015 Jul 23;16(1):147. doi: 10.1186/s13059-015-0711-4 (PMC4511969; doi:10.1186/s13059-015-0711-4)
Supplement: Supplementary file 1 — Supplementary Material contains Supplementary Figs. S1–S15, Supplementary Tables S1–S17, Supplementary Note, and Supplementary References. [file 13059_2015_711_MOESM1_ESM.pdf]

Supplementary Material

Kiwi genome provides insights into evolution of a nocturnal lifestyle

Diana Le Duc[1,2], Gabriel Renaud[2], Arunkumar Krishnan[3], Markus Sällman Almén[3], Leon Huynen[4], Sonja J. Prohaska[5], Matthias Ongyerth[2], Bárbara D. Bitarello[6], Helgi B. Schiöth[3], Michael Hofreiter[7], Peter F. Stadler[5], Kay Prüfer[2], David Lambert[4], Janet Kelso[2] & Torsten Schöneberg[1]

[1]Institute of Biochemistry, Medical Faculty, University of Leipzig, 04103 Leipzig, Germany

[2]Department of Evolutionary Genetics, Max Planck Institute for Evolutionary Anthropology, 04103 Leipzig, Germany

[3]Unit of Functional Pharmacology, Dept. of Neuroscience, Uppsala University Box 593, Husargatan 3, 751 24 Uppsala, Sweden

[4]Griffith School of Environment and School of Biomolecular and Physical Sciences, Griffith University, NATHAN QLD 4111, Queensland, Australia

[5]Department of Computer Science, and Interdisciplinary Center for Bioinformatics, University of Leipzig, 04103 Leipzig, Germany

[6] Department of Genetics and Evolutionary Biology, University of São Paulo, 05508-090 São Paulo, SP, Brazil

[7]Adaptive Evolutionary Genomics, Institute for Biochemistry and Biology, University Potsdam, 14469 Potsdam, Germany

The pdf file includes:

Supplementary Figures 1-15

Supplementary Tables 1-17

Supplementary Note

Supplementary References

Contents

Supplementary Figures 4

Supplementary Tables 21

Supplementary Note 70

- Sampling, DNA library preparation and sequencing 70
- Filtering and read correction 72
- Genome assembly 73
- Whole genome alignments 74
- Genome coverage and estimation of genome size 75
- Measure for heterozygosity 78
- Mitochondrial genome assembly 80
- RNA sequencing 83
- *De novo* gene prediction and gene annotation 83
- Orthologs and Ka/Ks calculation 84
- Gene Ontology and rapidly evolving genes 86
- Gene families assignment using TreeFam 87
- Gene families evolution using CAFE 88
- Detection and classification of the membrane proteome 90
- Phylogenetic analysis 92
- Vision analysis 95
- Olfaction analysis 100
- Limb development analysis 105

Supplementary References 112

Supplementary Figures

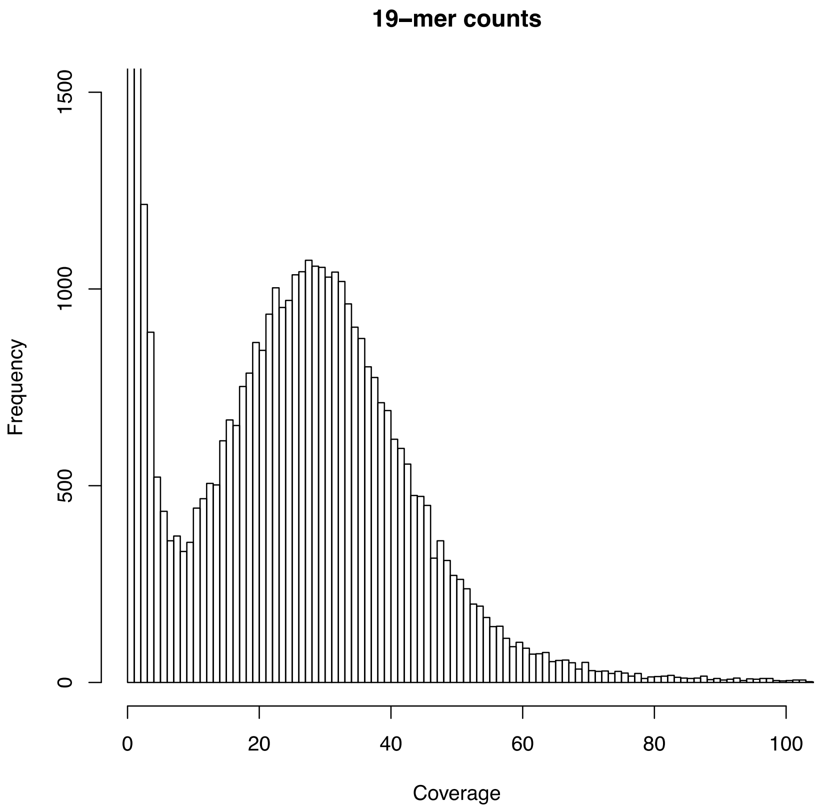

Supplementary Fig. S1. 19-mer frequency analysis of kiwi genome. The *k-mer* distribution for putative errors rises outside the main distribution of the *k-mer* representing the majority of the data and ends at coverage 5. The true k-mer distribution has a mean of 31-fold. The expected coverage (C_k) of a *k-mer* (of size K) in the genome using reads of length L is $C_k = D*(L-K+1)/L$ [1], where D is the real sequencing depth. The real sequencing depth is thus estimated to be 37-fold.

A

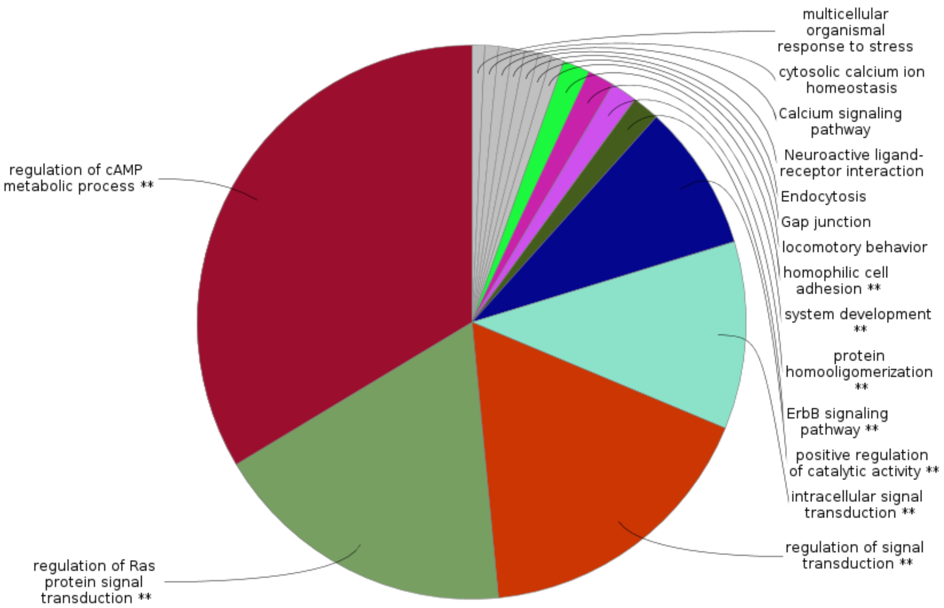

B

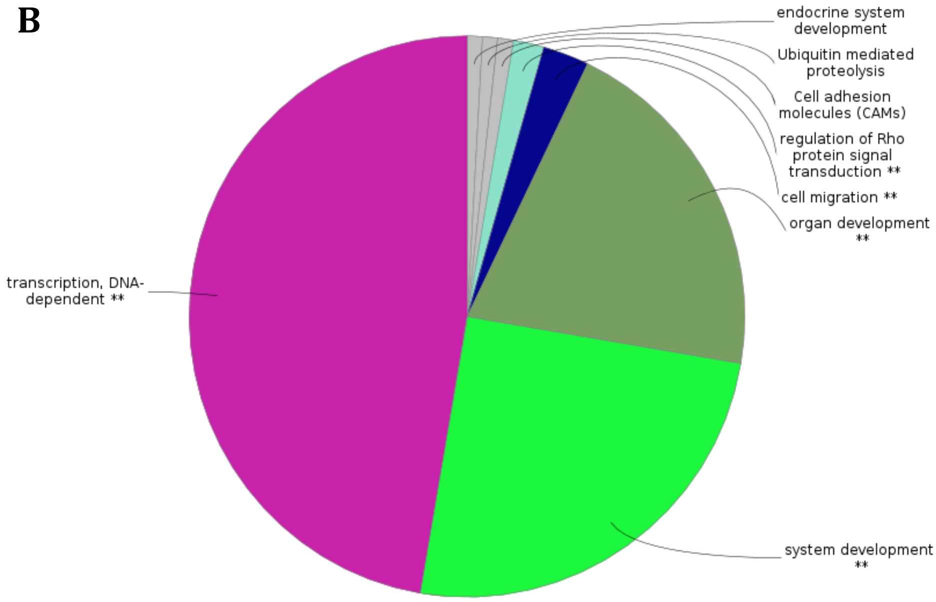

Supplementary Fig. S2. Enriched non-redundant biological terms for gene families with significantly different sizes in kiwi. GO enrichment was tested using the Pfam ID with most hits for the changed TreeFam family (as tested by CAFE [2]). The large clusters of genes corresponding to (A) expanded and (B) contracted gene families were grouped in a functional network and non-redundant biological terms with corrected FDR < 0.0001 were retrieved using ClueGO [3]; ** = more than 3 functionally related GO categories cluster in the same GO node.

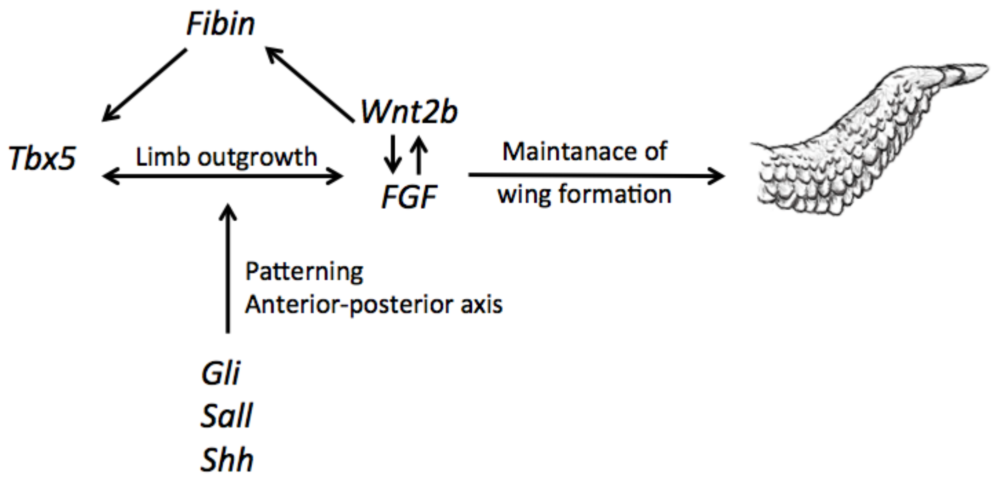

Supplementary Fig. S3. Pathways involved in wing development [4]. Genes, which belong to these pathways, were identified and manually surveyed in *AptMant0* (***Supplementary Table S12***). Coding regions were inspected and no obvious alterations were observed.

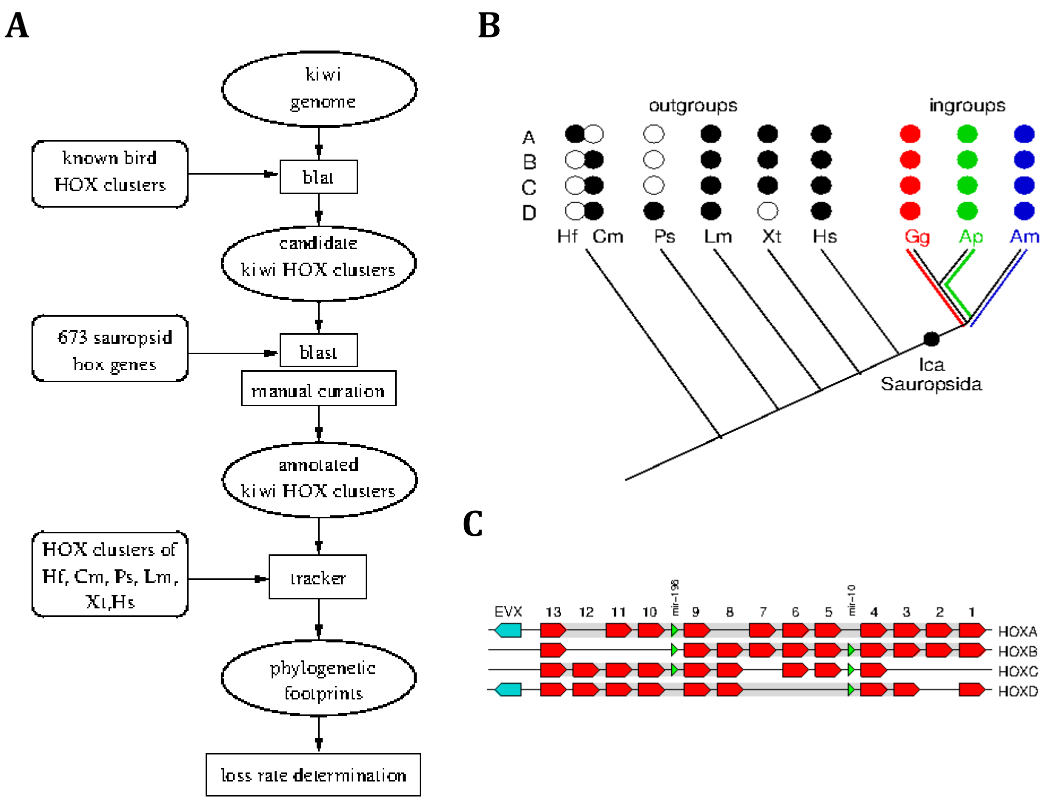

Supplementary Fig. S4. Workflow for kiwi HOX cluster annotation and phylogenetic footprinting. (A) Analysis pipeline. The *AptMant0* genome was searched for homologies with known bird HOX clusters (Gg – *Gallus gallus*, Ap – *Anas platyrhynchos*) using blat [5]. The following sequences were identified as HOX cluster fragments: scaffold151:16500000-16780000 (HOXA); scaffold5189, scaffold16558, C19529309 and scaffold171:1-40000 (HOXB); scaffold2266, scaffold2703 and C20176537 (HOXC); scaffold95:16600000-16800000 and scaffold9799 (HOXD). 673 sauropsid HOX protein sequences with cluster and paralog group assignments were retrieved from GenBank [6] and mapped to the candidate clusters with tblastn [5]. The hits were manually curated to determine the exact position of the start and stop codons. Phylogenetic footprinting was performed on HOX clusters from five outgroup species (a shark, Hf – *Heterodontus francisci* or Cm – *Callorhinchus milii*; a basal *Actinopterygian*, Ps – *Polypterus senegalus*, a coelacanth, Lm – *Latimeria menadoensis*, an amphibian, Xt – *Xenopus tropicalis*, and Hs – *Homo sapiens*) and three ingroup species (Gg – *Gallus gallus*, Ap – *Anas platyrhynchos*, Am – *Apteryx mantelli*) for A, B, C and D clusters separately using tracker2 [7] (standard settings, direct comparisons among bird sequences excluded).

(B) Evaluation of footprint losses. Clusters available for footprinting are marked by full circles in contrast to missing clusters (empty circles). A footprint is counted as ancestral if at least two out of four outgroup species share a sequence of at least 15 nt. Differential

loss of ancestral footprints was studied by comparing their presence in *Galliformes* with their presence in *Apteryx*.

(C) Overview of the HOX cluster of *Apteryx mantelli*. The kiwi has four HOX clusters (HOXA, HOXB, HOXC and HOXD) with 39 *HOX* genes (red arrows), evx1 and evx2 (turquoise arrows) and six microRNA genes (green triangles) belonging to two distinct microRNA families. The paralogous group assignments of *HOX* genes are given at the top. Gene complement, gene order, and orientation are identical to the proposed *HOX* cluster of the sauropsid ancestor [8]. Regions used for phylogenetic footprinting are shown as grey shades.

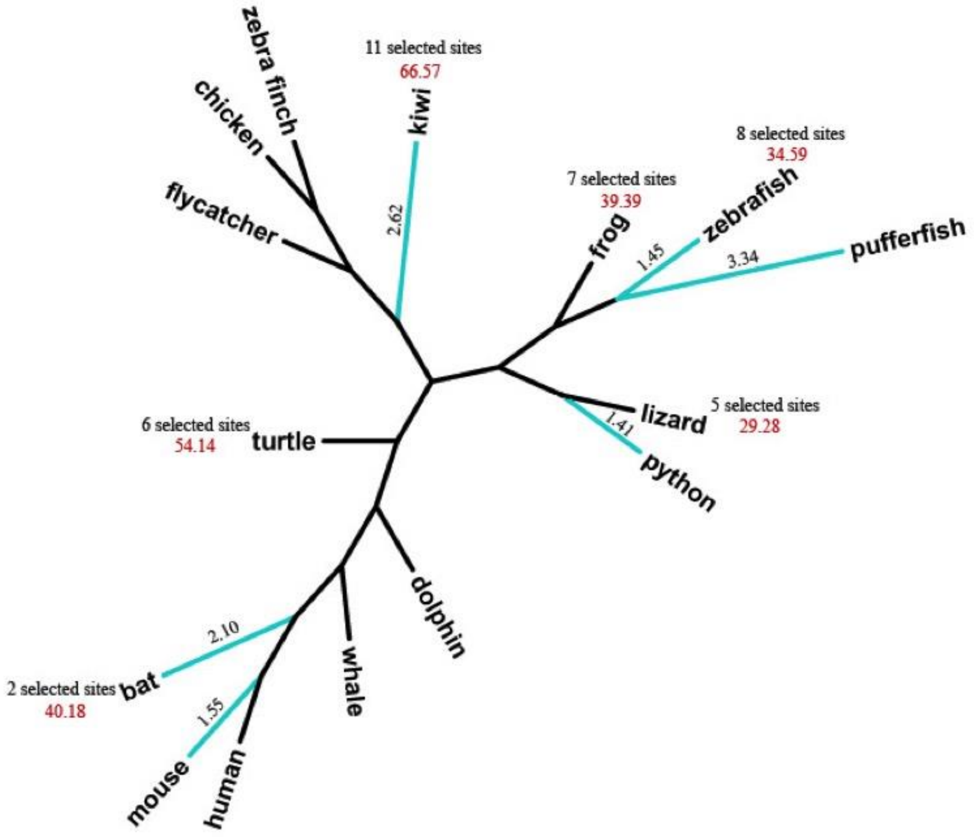

Supplementary Fig. S5. Phylogenetic tree for the species used in the *fibin* gene selection analysis. PAML [9] branch analysis showed signals of positive selective pressure on branches highlighted in blue ($\omega_{background}$ = 1.07, $\omega_{foreground}$ = 2.13, LRT = 4.186, p-value = 0.04). Values on the branches are estimated using the free ratio model implemented in CODEML (model = 1). The number of sites where evidence for positive selection was detected in the PAML branch-site model is shown next to the corresponding species. Values in red represent LRT between the branch-site model with free ω estimation and the model with ω fixed to the neutral value of 1.

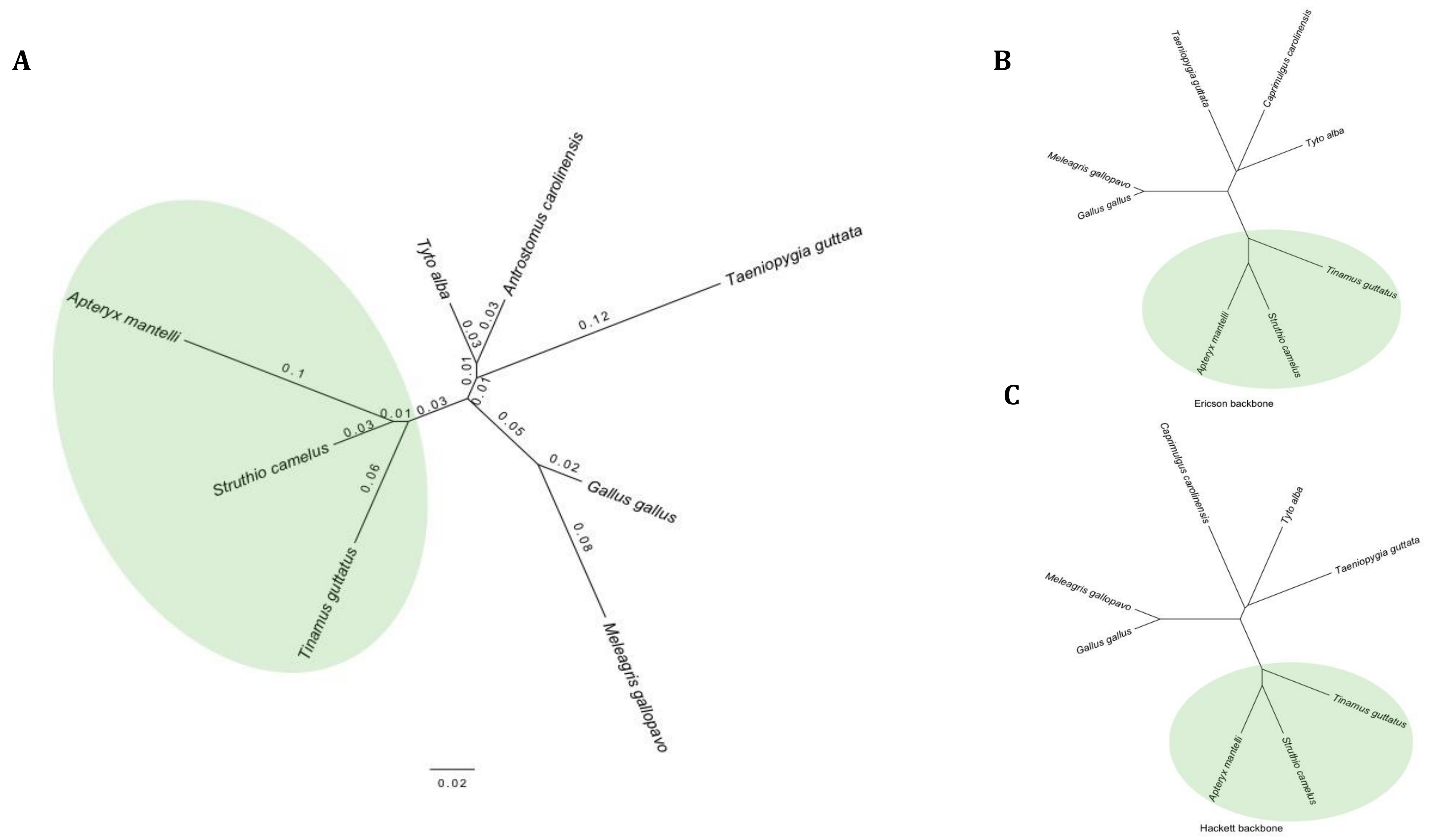

Supplementary Fig. S6. Phylogeny constructed to validate the position of *Tinamous guttatus* in the *Palaeognathae* clade (highlighted in light green; see Figure 1). (A) 3,939 orthologs (14,104,428 bp) in the 8 bird species were used for the tree [10]. 100 bootstraps were performed and each branch received 100% support. (B) Same species were selected in birdtree (http://birdtree.org/) and 100 trees were generated using Ericson backbone and (C) Hackett backbone [11]. All generated trees supported the same topology.

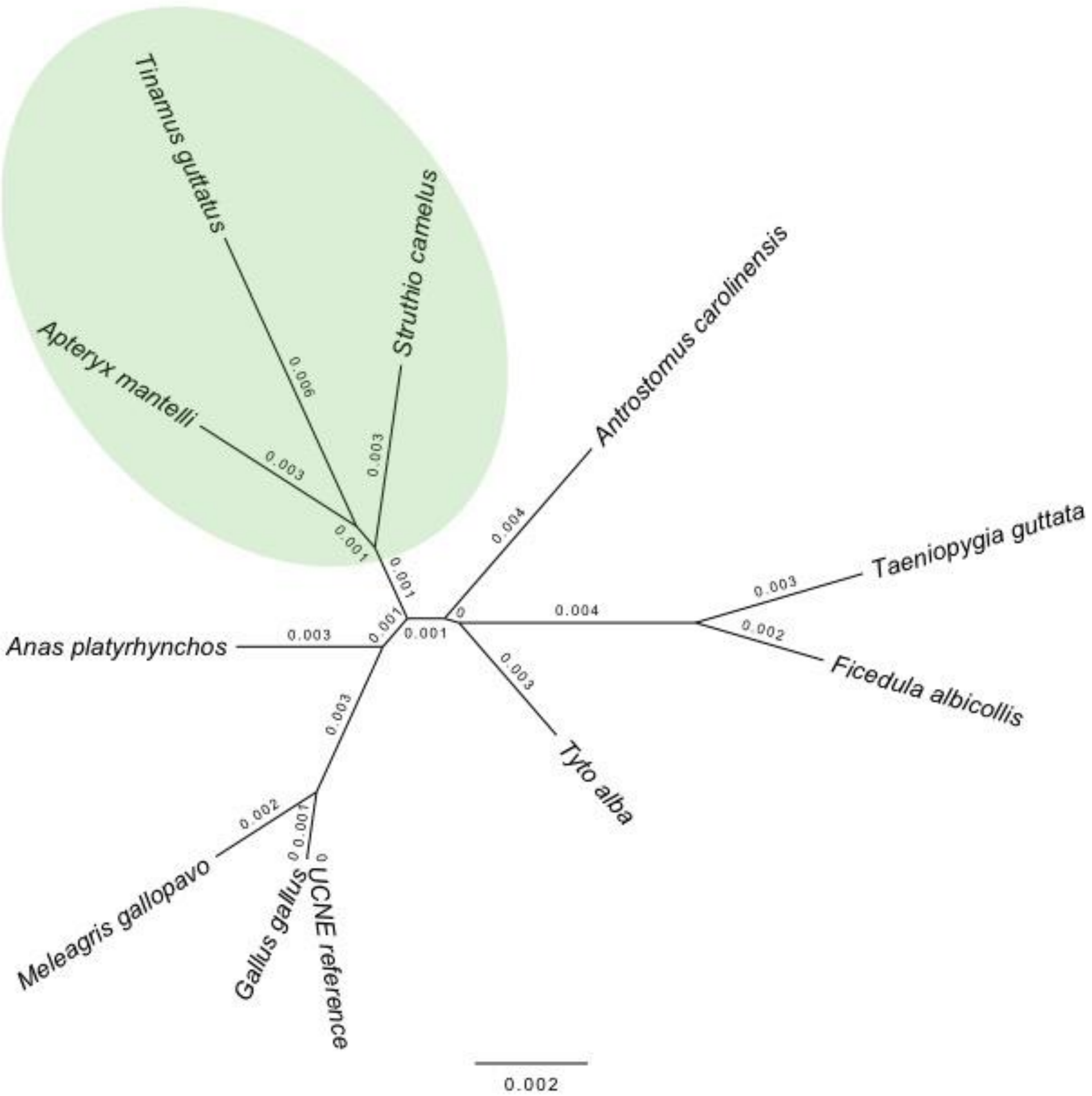

Supplementary Fig. S7. Phylogeny [10] built using 3,076 (1,011,462 bp) ultra-conserved non-coding regions [12], which had in all investigated genomes a length of at least 95% of the reference UCNE [12]. 100 bootstraps were performed and each branch received 100% bootstrap support. Galgal4 genome from Ensembl was used as a control of the orthologous region assignment to the reference chicken UCNE. *Palaeognathae* clade is highlighted in light green.

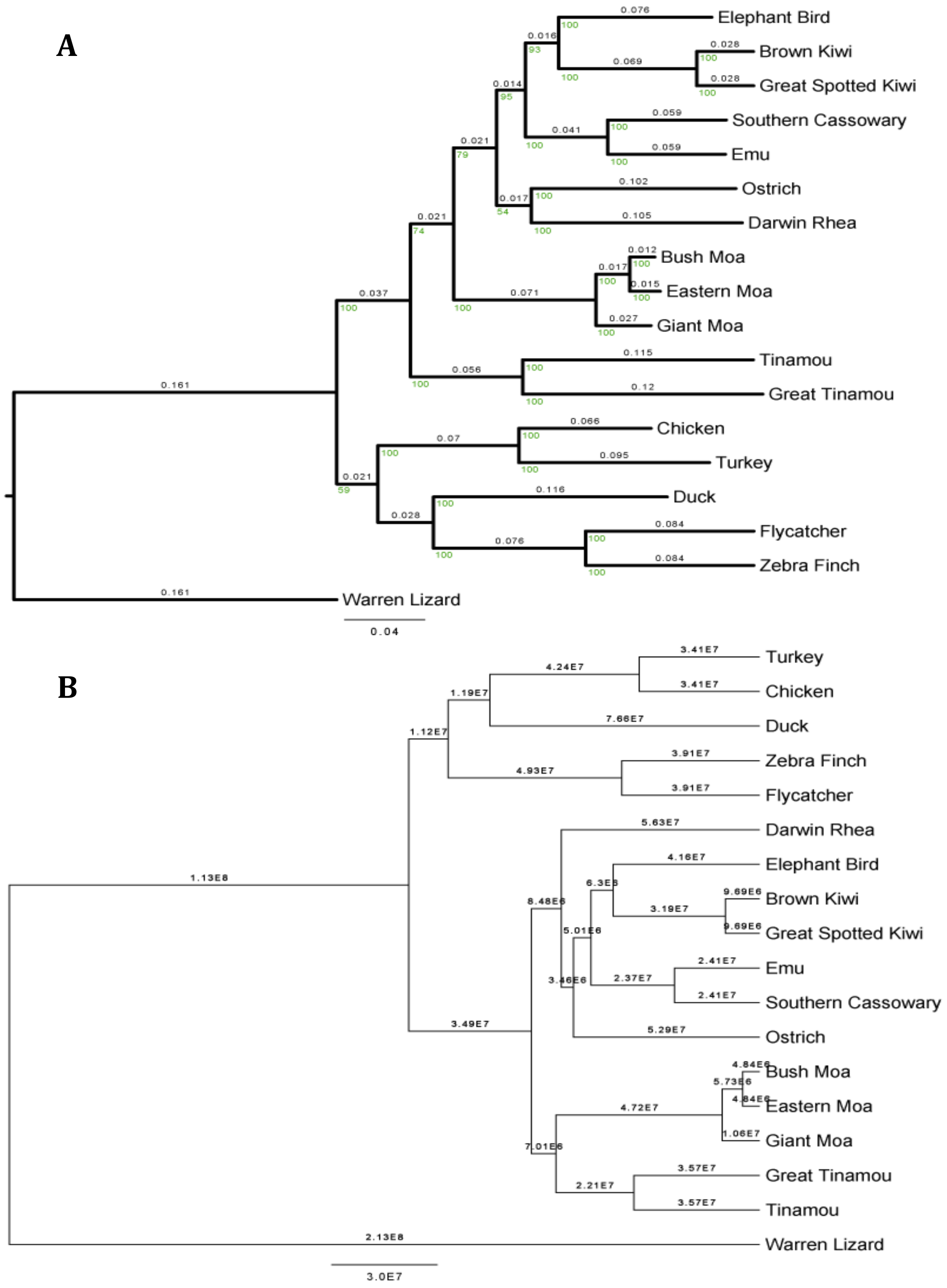

Supplementary Fig. S8. Phylogenetic trees constructed using mitochondrial genomes (GenBank Accession IDs in ***Supplementary Table S17***). (A) Maximum likelihood tree computed with PAUP* [10]. Numbers in green under each branch represent the bootstrap support (%) from 100 replicates. The numbers on each branch represent the branch lengths based on the number of mutations per 100 bp. (B) Molecular phylogeny of same mitochondrial genomes calculated using Bayesian inference with BEAST [13]. Branch lengths give the split time in years.

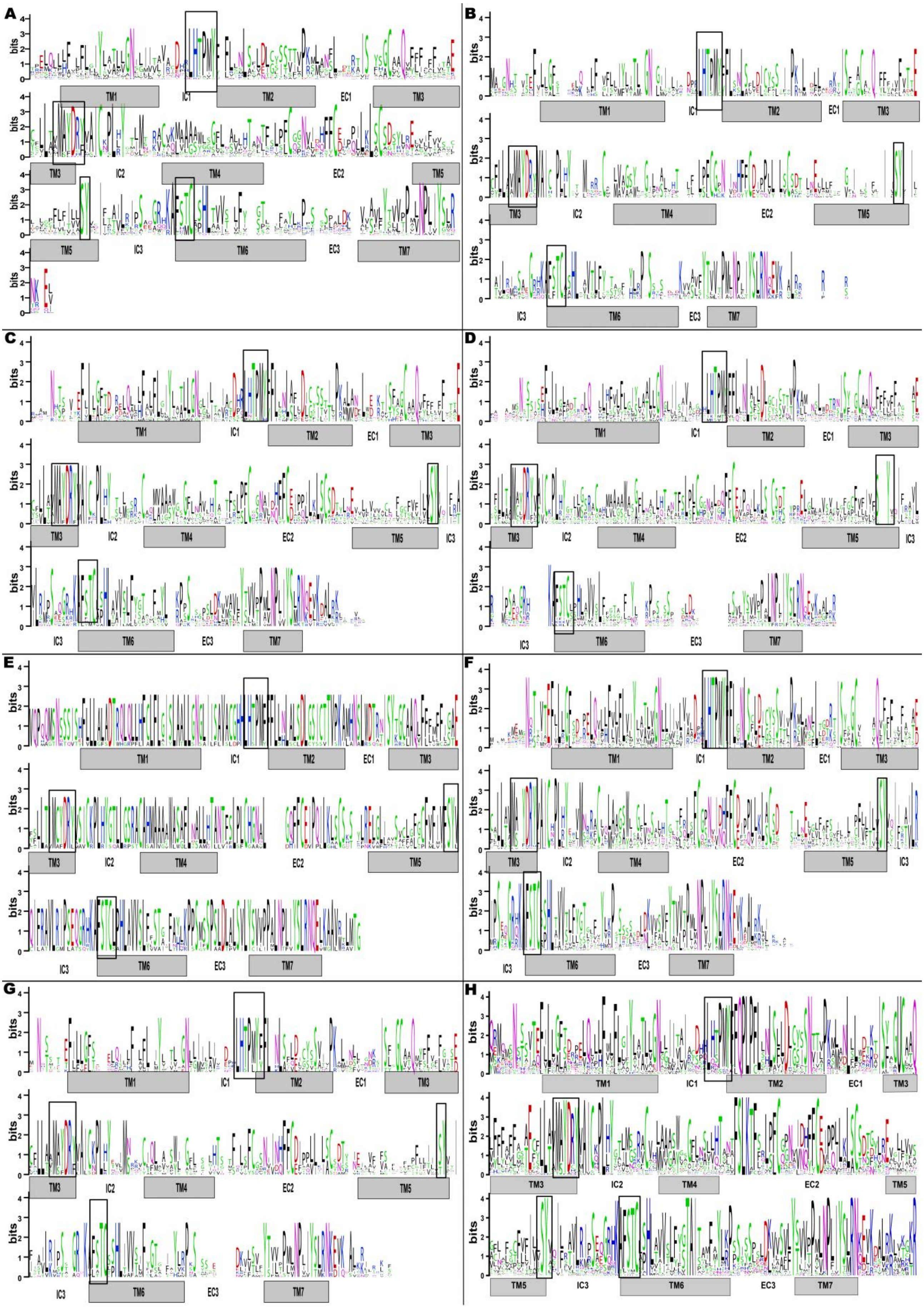
A
B
C
D
E
F
G
H
bits
TM1
IC1
TM2
EC1
TM3
IC2
TM4
EC2
TM5
IC3
TM6
EC3
TM7

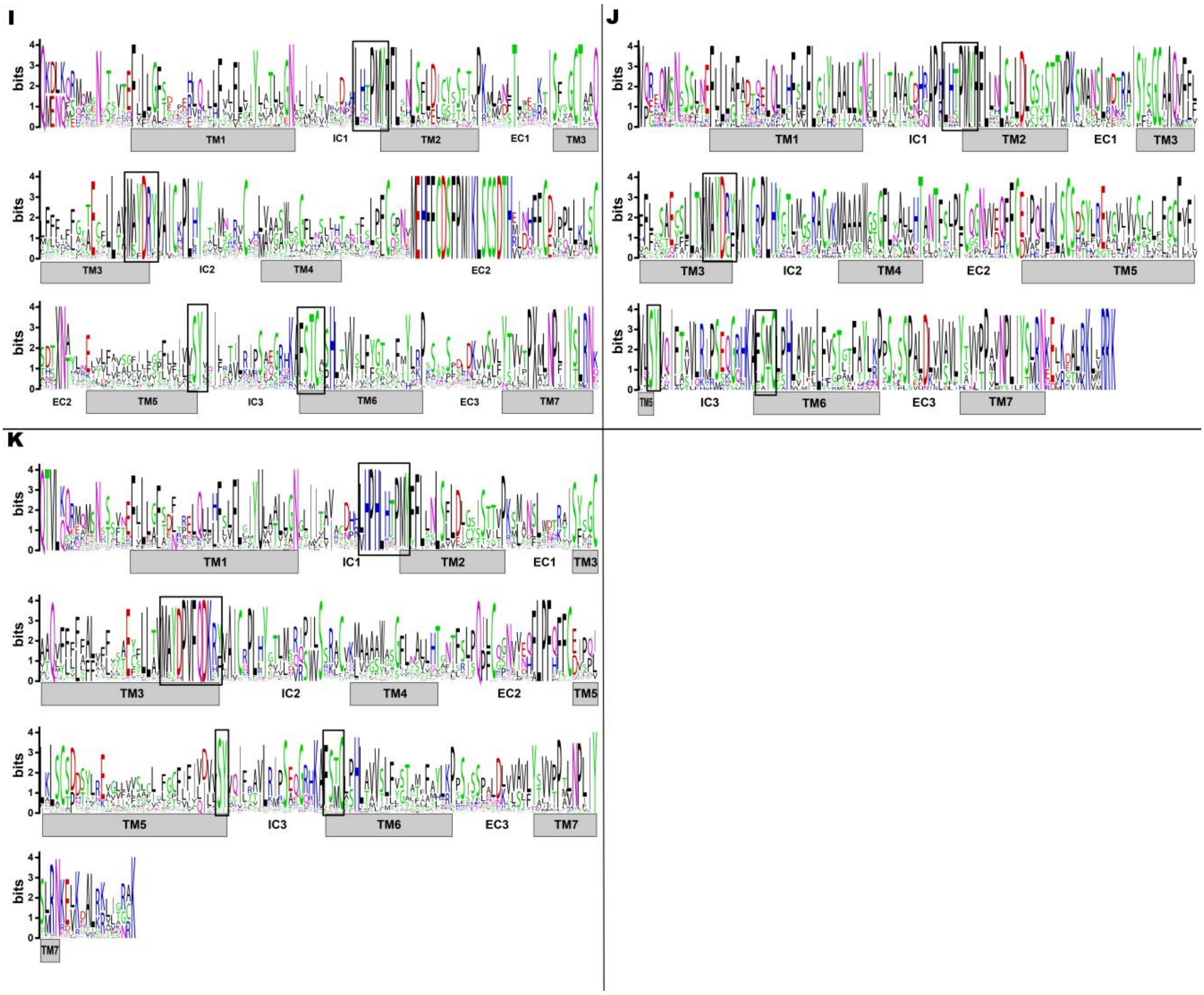

Supplementary Fig. S9. Amino acid sequence conservation in the reptilian and avian γ OR gene repertoires. Sequence logos of (A) kiwi, (B) chicken, (C) turkey, (D) flycatcher, (E) zebra finch, (F) Chinese softshell turtle, (G) green anole, (H) barn owl, (I) chuck-will's-widow, (J) ostrich, and (K) tinamou. Logos were generated using the program WebLogo [14]. Heights of amino acid letters represent the relative frequency at a given position and the overall height indicates the level of sequence conservation. Transmembrane regions (TM), intracellular (IC), and extracellular (EC) domains are marked according to sequence conservation and have not been verified experimentally in this study. Characteristic motifs and submotifs for ORs are represented by black boxes.

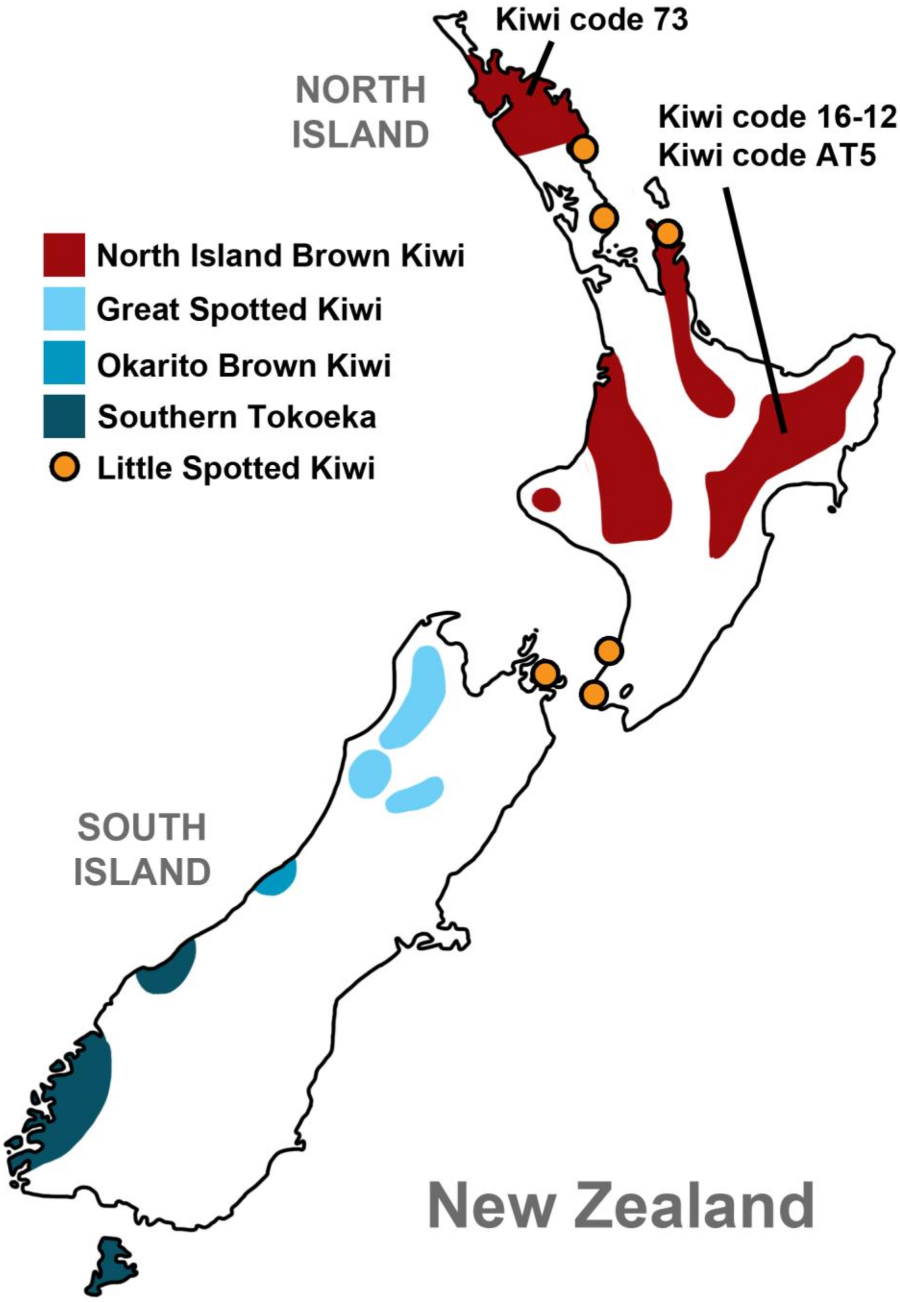

Supplementary Fig. S10. Present distribution of kiwi (*Apteryx* spp.) in New Zealand (source "Kiwi (*Apteryx* spp.) recovery plan 2008-2018" [15]). The three sequenced individuals originate from the far North (kiwi code 73) and central part – Lake Waikaremoana (kiwi code AT5 and kiwi code 16-12) of North Island. They were sampled in 1986 (kiwi code 73) and 1997 (kiwi code AT5 and 16-12) in 'operation nest egg' carried out by Rainbow and Fairy Springs, Rotorua. The genome was assembled with iwi approval.

A

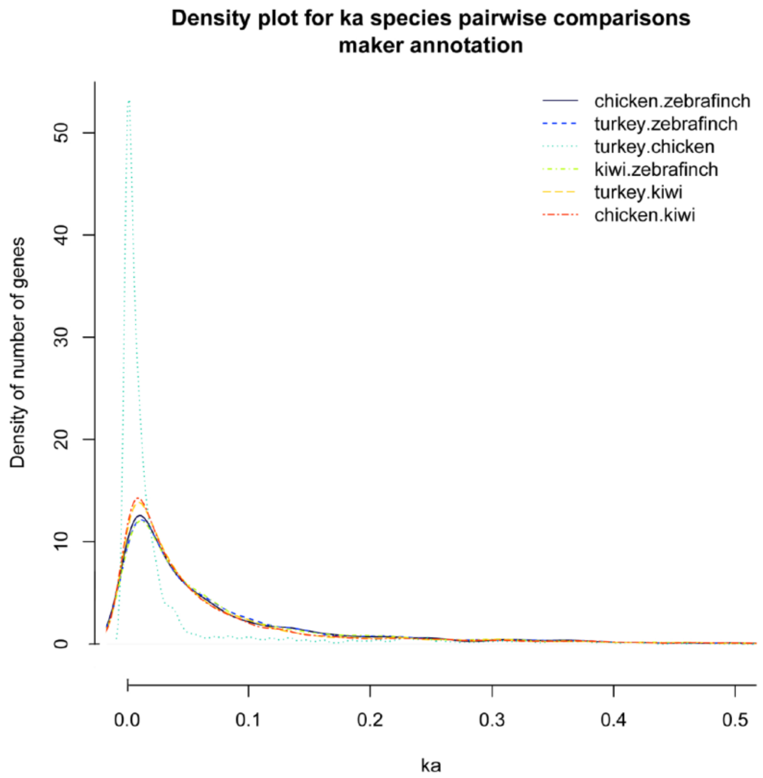

B

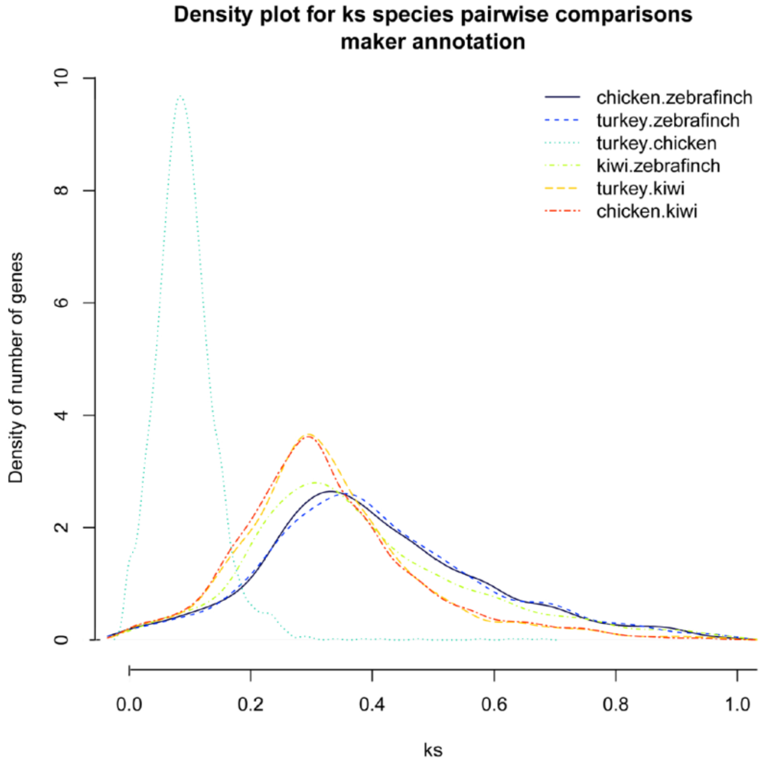

Supplementary Fig. S11. Distribution of (A) Ka and (B) Ks on the set of 3,754 orthologous genes in chicken, zebra finch, turkey, and kiwi, which presented no frame shifting indels after multiple sequence alignment. Ka values are much lower than Ks, confirming that non-synonymous mutations occur with a lower frequency.

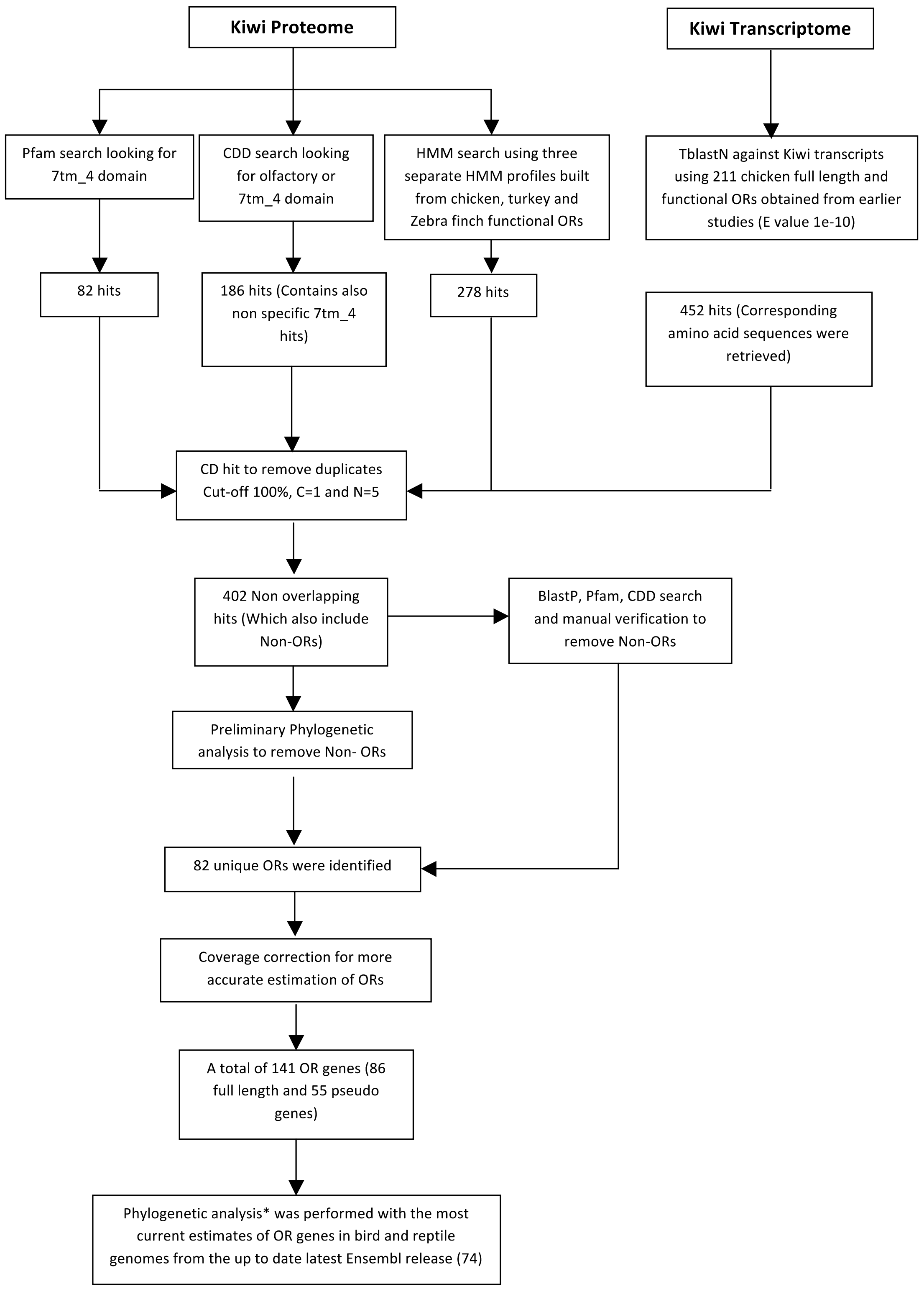

Supplementary Fig. S12. Flowchart depicting the olfactory receptors annotation process. * For phylogenetic analysis (see Figure 3), we downloaded all bird and reptile genomes present in Ensembl 74 [16] and ostrich, tinamou, barn owl, and chuck-will's-widow from GigaDB [17]. ORs for all investigated genomes were annotated using the same approach mentioned in the flowchart. The major difference from previous estimates (Ensembl 73) was found in the estimates for chicken, where, after curation, most of the artificial duplicates that had been mapped on the chromosome 'unknown' were removed. Thus, for phylogeny analysis, the estimates from the Ensemble 74 for all bird and reptile genomes were kept (see Figure 3).

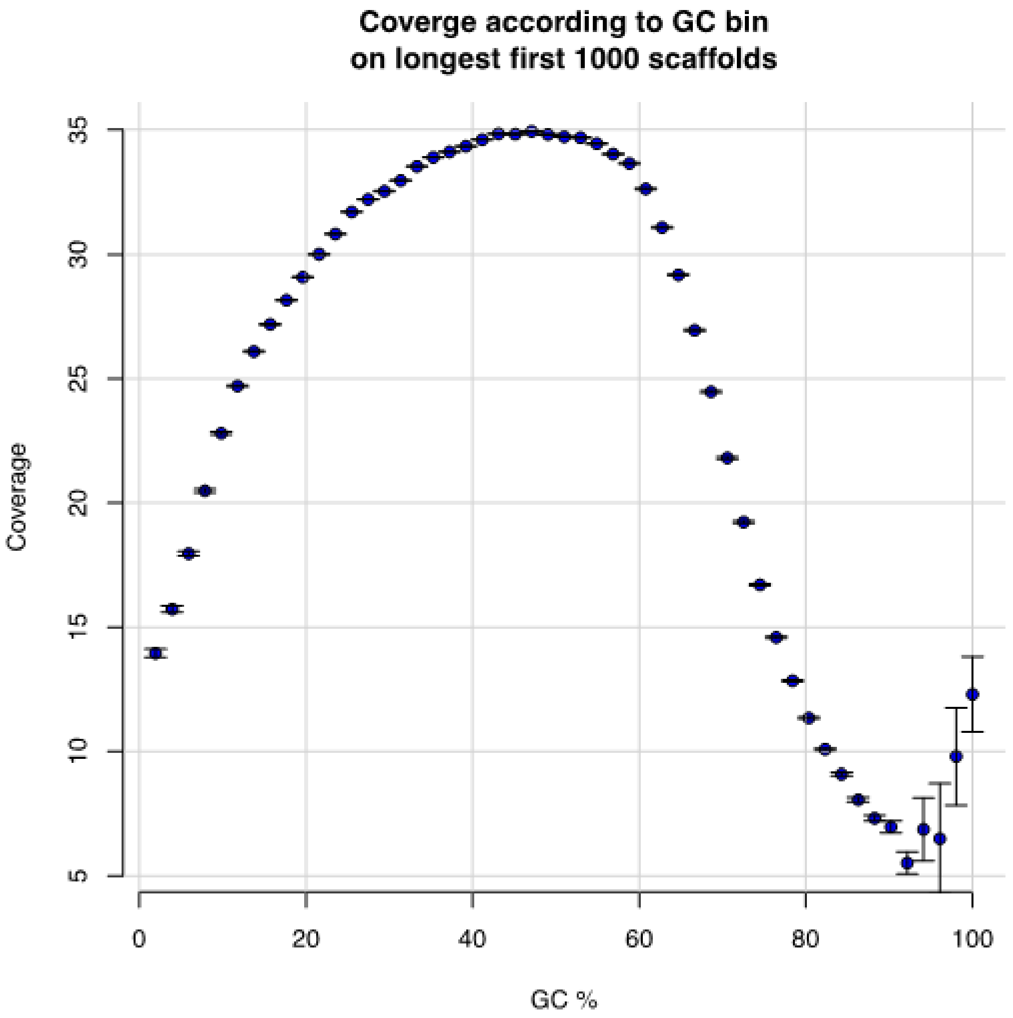

Supplementary Fig. S13. Mean coverage for GC content calculated after realigning error-corrected reads from short-insert-size libraries to the 1,000 longest scaffolds of the assembled genome. Error bars represent 95% binomial confidence intervals. Coverage for regions with GC content between 25% and 62% correspond to the coverage observed genome wide (35.85-fold).

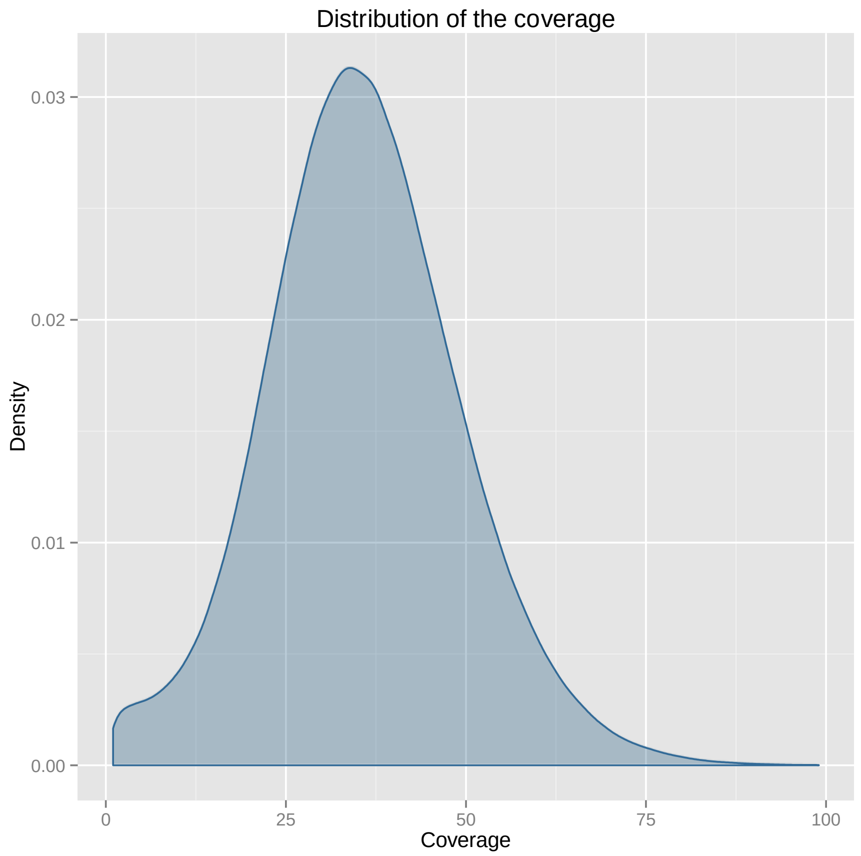

Supplementary Fig. S14. Coverage density distribution calculated after realigning short-insert-size libraries reads to the assembled genome. The figure shows the distribution on a subset of 1% of the data by using only reads that mapped to the first 1,000 longest scaffolds and filtered for a mapping quality higher than 30. The mean coverage is 35.85-fold.

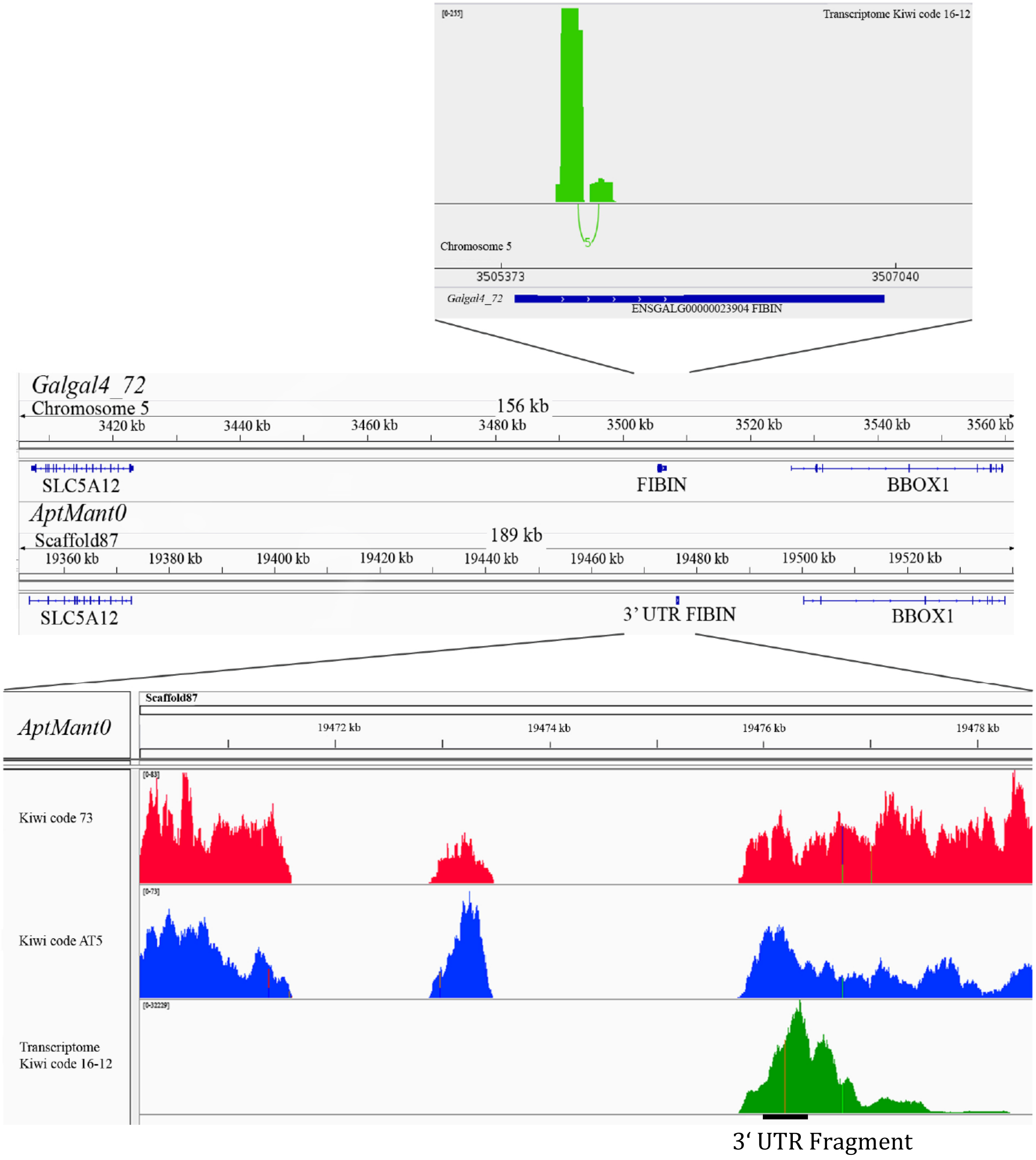

Supplementary Fig. S15. Schematic view of *fibin* region coverage. The upper box (A) shows transcriptomic reads (kiwi code 16-12) aligned to the chicken genome (Galgal4_72). (B) Comparison of the *fibin* gene region between *Gallus gallus* and *Apteryx mantelli*. The synteny and physical distances between genes are conserved in both species. The lower box (C) shows genomic coverage on scaffold87 in the 3' UTR *fibin* region. For primer sequences used to amplify the *fibin* coding sequence refer to Supplementary Table S14.

Supplementary Tables

Supplementary Table S1. Overview of generated sequencing data

[1] Library sequenced on Illumina MySeq Technology with paired-end reads 76 bp.

* Reads from individual AT5 were used only for estimating heterozygosity and were not included in the genome assembly.

** Reads from mate-paired-end libraries were used only for scaffolding and are not part of the consensus sequence.

Insert size	Raw data (Gb)	Data used for contig assembly (Gb)	Kiwi individual code
350	45.99	*	AT5
240	42.88	30.54	73
420	32.56	16.7	73
800	7.28	5.29	73
2,000[1]	0.75	**	73
3,000	14.13	**	73
4,000	15.97	**	73
7,000	4.39	**	16-12
9,000	2.76	**	16-12
11,000	46.85	**	16-12
13,000	35.83	**	16-12
**Sum**	**249.39**	**52.53**	

Supplementary Table S2. SOAP *de novo* assembly metrics after stepwise inclusion of different insert size library data.

**Step**	**Library**	**N50 (bp)**	**N90 (bp)**	**Average length (bp)**	**Longest scaffold (bp)**	**Total length (bp)**
Contig	240, 420, 800 (corrected)	1,550	281	731	22,713	1,226,568,938
Scaffold 1	240	13,640	1,143	5,190	170,761	1,173,734,991
Scaffold 2	420	25,945	1,946	7,651	322,977	1,253,654,343
Scaffold 3	800	31,909	2,394	8,887	368,910	1,285,674,594
Scaffold 4	2,000	32,914	2,581	9,705	368,990	1,285,409,640
Scaffold 5	3,000	66,802	4,737	14,334	969,600	1,436,782,364
Scaffold 6	4,000	154,116	9,567	20,545	3,273,144	1,541,358,432
Scaffold 7	7,000	377,029	13,316	23,816	4,665,740	1,565,824,628
Scaffold 8	9,000	931,848	17,816	26,056	11,355,285	1,582,473,408
Scaffold 9	11,000	3,663,049	29,572	33,089	38,853,025	1,662,413,992
Scaffold10	13,000	5,026,352	35,043	37,585	73,122,679	1,747,282,849

Supplementary Table S3. Assembly metrics after gap closing (*AptMant0*).

Assumed genome size (bp)	1,650,000,000
Number of scaffolds	326,827
Total size of scaffolds (bp)	1,595,278,775
Total scaffold length as percentage of assumed genome size	96.68%
Longest scaffold (bp)	63,182,071
Number of scaffolds > 1K nt	24,710
Number of scaffolds > 10K nt	6,641
Number of scaffolds > 100K nt	1,040
Number of scaffolds > 1M nt	221
Number of scaffolds > 10M nt	32
N50 scaffold length (bp)	3,956,354
Scaffold %A	25
Scaffold %C	18
Scaffold %G	18
Scaffold %T	25
Scaffold %N	13
Number of contigs	508,831
Total size of contigs	1,382,272,215
Longest contig	166,809
Number of contigs > 1K nt	146,153
Number of contigs > 10K nt	40,984
Number of contigs > 100K nt	69
Number of contigs > 1M nt	0
N50 contig length	16,480

Supplementary Table S4. Genome sizes (A) calculated according to the C-value estimates. (http://www.genomesize.com) and (B) assembled in the Avian Phylogenomics Project (http://avian.genomics.cn/en/).

A)

**Species**	**Common name**	**C value (pg)**	**Estimated genome size (Gb)**
Apteryx mantelli	Kiwi		1.65
Struthio camelus	Ostrich	2.16	2.11
Dromaius novaehollandiae	Emu	1.55	1.52
Crypturellus obsoletus	Brown tinamou	1.35	1.33
Meleagris gallopavo	Turkey	1.31	1.28
Gallus domesticus	Domestic chicken	1.25	1.22
Taeniopygia guttata	Zebra finch	1.25	1.22
Archilochus alexandri	Black-chinned hummingbird	0.91	0.89
**Average over 358 bird species**		**1.38±0.01**	**1.35**

B)

**Species**	**Common name**	**Size of assembly (Gb)**	**N50**
Haliaeetus leucocephalus	Bald eagle	1.26	670 kb
Aptenodytes forsteri	Emperor penguin	1.26	5.1 Mb
Struthio camelus	Common ostrich	1.23	3.5 Mb
Pygoscelis adeliae	Adelie penguin	1.23	5.0 Mb
Taeniopygia guttata	Zebra finch	1.2	10 Mb
Egretta garzetta	Little egret	1.2	3.1 Mb
Charadrius vociferus	Killdeer	1.2	3.6 Mb
Falco peregrinus	Peregrine falcon	1.18	3.9 Mb
Tauraco erythrolophus	Red-crested turaco	1.17	55 kb
Picoides pubescens	Downy woodpecker	1.17	2 Mb
Pelecanus crispus	Dalmatian pelican	1.17	43 kp
Nipponia nippon	Crested ibis	1.17	5.4 Mp
Cathartes aura	Turkey vulture	1.17	35 kb
Phaethon lepturus	White-tailed tropicbird	1.16	47 kb
Podiceps cristatus	Great-crested grebe	1.15	30 kb
Phalacrocorax carbo	Great cormorant	1.15	48 kb
Leptosomus discolor	Cuckoo-roller	1.15	61 kb
Gavia stellata	Red-throated loon	1.15	45 kb
Cuculus canorus	Common cuckoo	1.15	3 Mb
Cariama cristata	Red-legged seriema	1.15	54 kb
Antrostomus carolinensis	Chuck-will's-widow	1.15	45 kb
Tyto alba	Barn owl	1.14	51 kb
Phoenicopterus ruber	American flamingo	1.14	37 kb

Species	Common name	Size of assembly (Gb)	N50
Ophisthocomus hoazin	Hoatzin	1.14	2.9 Mb
Nestor notabilis	Kea	1.14	37 kb
Haliaeetus albicilla	White-tailed eagle	1.14	56 kb
Fulmarus glacialis	Northern fulmar	1.14	46 kb
Balearica regulorum	Grey-crowned crane	1.14	51 kb
Manacus vitellinus	Golden-collared manakin	1.12	2.5 Mb
Columba livia	Pigeon	1.11	3.2 Mb
Mesitornis unicolor	Brown mesite	1.1	46 kb
Melopsittacus undulatus	Budgerigar	1.1	10.6 Mb
Eurypyga helias	Sunbittern	1.1	46 kb
Corvus brachyrhynchos	American crow	1.1	6.9 Mb
Chaetura pelagica	Chimney swift	1.1	3.8 Mb
Calypte anna	Anna's hummingbird	1.1	4 Mb
Anas platyrhynchos	Peking duck	1.1	1.2 Mb
Chlamydotis macqueenii	Macqueen's bustard	1.09	45 kb
Colius striatus	Speckled mousebird	1.08	45 kb
Buceros rhinoceros	Rhinoceros hornbill	1.08	51 kb
Apaloderma vittatum	Bar-tailed trogon	1.08	56 kb
Pterocles gutturalis	Yellow-thoated sandgrouse	1.07	49 kb
Geospiza fortis	Medium ground finch	1.07	5.2 Mb
Merops nubicus	Carmine bee-eater	1.06	47 kb
Tinamus guttatus	White-throated tinamou	1.05	242 kb
Gallus gallus	Chicken	1.05	7.07 Mb
Acanthisitta chloris	Rifleman	1.05	64 kb
Meleagris gallopavo	Turkey	1.04	1.5 Mb

Supplementary Table S5. Statistics for chaining of the *AptMant0* assembly to the (A) chicken and (B) zebra finch chromosomes respectively.

A)

Chicken chromosome	Chromosome size	Chained sequence	Percentage covered	Different sites	Percentage difference
1	195,276,750	155,364,546	79.56	38,041,358	24.49
2	148,809,762	119,829,029	80.53	29,079,104	24.27
3	110,447,801	90,986,045	82.38	21,964,886	24.14
4	90,216,835	73,355,842	81.31	17,952,272	24.47
5	59,580,361	49,279,180	82.71	11,716,073	23.77
6	34,951,654	28,482,813	81.49	6,922,076	24.30
7	36,245,040	30,381,351	83.82	7,225,727	23.78
8	28,767,244	23,701,768	82.39	5,608,128	23.66
9	23,441,680	19,428,562	82.88	4,710,455	24.25
10	19,911,089	16,608,908	83.42	3,874,140	23.33
11	19,401,079	16,287,461	83.95	3,714,888	22.81
12	19,897,011	16,543,187	83.14	3,950,557	23.88
13	17,760,035	14,547,755	81.91	3,553,626	24.43
14	15,161,805	12,272,985	80.95	2,971,220	24.21
15	12,656,803	10,445,778	82.53	2,422,682	23.19
16	535,270	138,258	25.83	40,898	29.58
17	10,454,150	8,462,801	80.95	1,973,263	23.32
18	11,219,875	8,968,833	79.94	2,151,082	23.98
19	9,983,394	7,880,147	78.93	1,837,173	23.31
20	14,302,601	11,531,740	80.63	2,762,076	23.95
21	6,802,778	5,144,080	75.62	1,206,947	23.46
22	4,081,097	2,979,422	73.01	704,872	23.66
23	5,723,239	4,245,544	74.18	1,023,977	24.12
24	6,323,281	4,905,374	77.58	1,167,829	23.81
25	2,191,139	1,233,391	56.29	317,079	25.71
26	5,329,985	3,870,041	72.61	957,303	24.74
27	5,209,285	3,195,539	61.34	807,582	25.27
28	4,742,627	3,080,012	64.94	735,990	23.90
M	16,775	15,221	90.74	3,403	22.36
W	1,248,174	334,909	26.83	97,280	29.05
Z	82,363,669	55,592,343	67.50	14,484,655	26.06
**Total**	**1,003,052,288**	**799,092,865**	**79.67**	**193,978,601**	**24.27**

B)

Zebra finch chromosome	Chromosome size	Chained sequence	Percentage covered	Different sites	Percentage difference
1	118,548,696	95,588,030	80.63	23,621,907	24.71
1A	73,657,157	60,848,047	82.61	14,881,959	24.46
1B	1,083,483	517,521	47.76	142,769	27.59
2	156,412,533	126,553,387	80.91	30,948,025	24.45
3	112,617,285	92,496,642	82.13	22,480,218	24.30
4	69,780,378	56,292,102	80.67	13,944,087	24.77
4A	20,704,505	16,075,521	77.64	3,968,127	24.68
5	62,374,962	50,105,661	80.33	12,040,151	24.03
6	36,305,782	29,094,931	80.14	7,011,544	24.10
7	39,844,632	32,173,733	80.75	7,737,045	24.05
8	27,993,427	22,620,918	80.81	5,357,610	23.68
9	27,241,186	21,639,691	79.44	5,349,694	24.72
10	20,806,668	17,088,758	82.13	4,017,138	23.51
11	21,403,021	17,155,722	80.16	4,043,564	23.57
12	21,576,510	17,301,317	80.19	4,219,639	24.39
13	16,962,381	13,449,468	79.29	3,309,012	24.60
14	16,419,078	12,812,141	78.03	3,143,441	24.53
15	14,428,146	11,071,597	76.74	2,649,060	23.93
16	9,909	196	1.98	14	7.14
17	11,648,728	8,750,950	75.12	2,098,487	23.98
18	11,201,131	8,232,172	73.49	2,036,004	24.73
19	11,587,733	8,528,917	73.60	2,062,133	24.18
20	15,652,063	11,894,185	75.99	2,910,542	24.47
21	5,979,137	4,062,176	67.94	1,004,306	24.72
22	3,370,227	1,927,566	57.19	504,101	26.15
23	6,196,912	3,820,547	61.65	965,711	25.28
24	8,021,379	4,950,626	61.72	1,268,459	25.62
25	1,275,379	594,306	46.60	158,546	26.68
26	4,907,541	3,060,663	62.37	800,428	26.15
27	4,618,897	2,659,963	57.59	703,489	26.45
28	4,963,201	2,980,655	60.06	748,802	25.12
MT	16,853	14,879	88.29	3,387	22.76
Un	175,225,315	113,600,653	64.83	28,906,824	25.45
Z	72,861,351	51,784,845	71.07	13,554,298	26.17
**Total**	**1,195,695,586**	**919,748,486**	**76.92**	**226,590,521**	**24.64**

Supplementary Table S6. Gene families (TreeFam [18]) with significant difference in size on *Apteryx mantelli* branch (p-value < 0.005, as tested by CAFE [2]).

G.a. = Gasterosteus aculeatus, A.c. = Anolis carolinensis, P.c. = Pelodiscus sinensis, H.s. = Homo sapiens, M.m. = Mus musculus, O.a. = Ornithorhynchus anatinus, G.g. = Gallus gallus, A.p. = Anas platyrhynchos, M.g. = Meleagris gallopavo, T.g. = Taeniopygia guttata, F.a. = Ficedula albicollis, A.cs. = Antrostomus carolinensis, T.a. = Tyto alba, A.m. = Apteryx mantelli, S.c. = Struthio camelus, T.gt. = Tinamus guttatus, A.m. = Apteryx mantelli* manually curated*.*

Presented Pfam ID is the one with highest percentage of hits for the respective TreeFam gene family.

Families expanded in *Apteryx mantelli*

**Description**	**TreeFam ID**	**Pfam ID**	***G.a.***	***A.c.***	***P.s.***	***H.s.***	***M.m.***	***O.a.***	***G.g.***	***A.p.***	***M.g.***	***T.g.***	***F.a.***	***A.cs.***	***T.a.***	***A.m.***	***S.c.***	***T.gt.***	***A.m.****
Protein phosphatase 1, regulatory (inhibitor) subunit 9	TF105540	PF00595	5	3	3	2	2	7	2	3	2	2	2	2	2	8	1	2	4
E1A-binding protein p400/Snf2-related CBP activator	TF106424	PF00176	2	2	2	2	3	5	1	1	1	2	1	3	2	9	1	1	3
Carbohydrate phosphorylase	TF300309	PF00343	3	3	2	3	3	6	2	2	3	2	2	2	0	7	1	2	3
Protein of unknown function (DUF1162)	TF300316	PF06650	2	2	2	1	1	7	1	4	3	3	2	1	3	8	2	2	5
Dynamin central region	TF300362	PF01031	5	2	3	3	3	5	3	4	2	4	3	5	3	10	3	3	4

Description	TreeFam ID	Pfam ID	*G.a.*	*A.c.*	*P.s.*	*H.s.*	*M.m.*	*O.a.*	*G.g.*	*A.p.*	*M.g.*	*T.g.*	*F.a.*	*A.cs.*	*T.a.*	*A.m.*	*S.c.*	*T.gt.*	*A.m.**
GTPase-activator protein for Ras-like GTPase	TF313078	PF00616	3	2	4	3	3	8	2	4	3	3	3	2	2	11	3	3	10
Ion channel family	TF313555	PF00520	6	5	3	3	3	7	3	4	3	4	3	3	3	11	3	2	4
Calcium-activated BK potassium channel alpha subunit	TF314283	PF03493	5	4	4	4	4	14	4	4	4	3	3	2	2	11	4	4	5
HEAT repeat domain	TF315201	PF02985	0	4	1	4	3	3	2	1	2	1	2	1	0	5	1	1	5
GHH signature containing HNH/Endo VII superfamily nuclease toxin	TF316833	PF15636	5	4	3	4	4	10	4	4	6	4	4	5	4	11	4	3	5
TPR repeat	TF323569	PF13414	1	1	1	2	1	6	1	1	2	2	2	1	1	5	1	1	5
Helicase conserved C-terminal domain	TF324610	PF00271	1	1	1	2	1	1	1	1	1	1	1	0	1	6	1	0	4
Pleckstrin homology domain	TF329258	PF00169	5	1	1	1	1	3	1	1	2	2	1	1	0	6	1	1	2
Zinc finger, C3HC4 type (RING finger)	TF329577	PF13923	2	2	1	0	0	2	0	1	1	2	0	1	1	11	1	1	6

Description	TreeFam ID	Pfam ID	*G.a.*	*A.c.*	*P.s.*	*H.s.*	*M.m.*	*O.a.*	*G.g.*	*A.p.*	*M.g.*	*T.g.*	*F.a.*	*A.cs.*	*T.a.*	*A.m.*	*S.c.*	*T.gt.*	*A.m.**
Sodium:neurotransmitter symporter family	TF342680	PF00209	8	4	3	6	5	8	5	3	5	5	4	4	4	11	4	3	10
Sea anemone cytotoxic protein	TF344188	PF06369	3	0	0	0	0	1	1	1	0	0	0	0	0	5	1	1	3
RhoGEF domain	TF351276	PF00621	3	2	3	2	2	2	2	2	5	4	2	3	2	7	2	2	4
SH2 domain	TF354288	PF00017	5	5	5	6	5	8	6	6	6	5	4	7	4	12	4	4	12
Vinculin family	TF313686	PF01044	5	7	9	6	6	13	7	6	8	7	6	4	7	13	6	6	8
RIH domain	TF312815	PF01365	4	3	5	3	3	15	3	3	4	5	3	4	6	10	4	5	5
Filamin/ABP280 repeat	TF313685	PF00630	5	4	3	4	3	5	1	3	1	2	2	1	1	6	1	1	4
G8 domain	TF316575	PF10162	2	2	3	2	2	8	3	3	4	2	2	3	3	9	4	3	7
Putative GTPase activating protein for Arf	TF325156	PF01412	4	4	4	3	12	8	5	5	4	3	3	2	2	9	3	4	6
CAP-Gly domain	TF326096	PF01302	5	4	4	5	4	8	3	3	4	4	3	5	2	9	3	4	5
Hsp70 protein	TF329492	PF00012	2	2	5	3	2	3	2	3	2	3	2	2	2	6	1	2	4
Calponin homology domain	TF329881	PF00307	4	2	1	1	1	5	1	2	2	1	1	0	2	6	1	2	3
Ankyrin repeats (3 copies)	TF344032	PF12796	2	4	4	3	3	4	2	3	2	1	3	2	2	6	1	1	2
Homeobox domain	TF350571	PF00046	15	7	8	19	12	8	4	5	4	6	5	4	3	11	5	5	9
Cadherin domain	TF331809	PF00028	13	7	11	14	15	12	7	8	6	5	10	6	5	12	6	6	8

Description	TreeFam ID	Pfam ID	*G.a.*	*A.c.*	*P.s.*	*H.s.*	*M.m.*	*O.a.*	*G.g.*	*A.p.*	*M.g.*	*T.g.*	*F.a.*	*A.cs.*	*T.a.*	*A.m.*	*S.c.*	*T.gt.*	*A.m.**
Epidermal growth factor receptor/v-erb-b2 erythroblastic leukemia viral oncogene	TF106002	PF01030	7	4	4	4	5	9	3	3	5	5	3	2	3	7	2	3	4
BAR domain	TF313542	PF03114	4	3	3	3	3	3	3	2	6	3	3	2	3	7	2	2	4
Beige/BEACH domain	TF313658	PF02138	3	2	3	2	2	8	3	2	3	4	2	4	3	7	3	3	4
Ubiquitin	TF314412	PF00240	2	2	4	5	6	1	3	4	2	2	2	2	2	7	3	3	5
RhoGAP domain	TF315892	PF00620	8	6	3	7	4	11	3	3	2	3	4	3	2	7	2	2	3
Plasma-membrane choline transporter	TF313325	PF04515	7	5	5	11	5	12	4	4	3	4	4	3	2	8	3	4	7
Tubulin/FtsZ family, GTPase domain	TF330882	PF00091	3	5	7	3	2	3	3	2	2	1	2	2	2	8	4	3	7
Glycosyl hydrolases family 31	TF314577	PF01055	3	5	2	6	3	16	4	5	4	7	4	3	3	9	4	5	6
von Willebrand factor type D domain	TF343473	PF00094	2	3	8	3	4	14	2	2	3	3	2	7	6	9	4	5	8
Coagulation Factor Xa inhibitory site	TF332034	PF14670	2	2	2	2	2	4	2	4	4	3	2	1	1	5	1	2	4
Dispanin	TF334894	PF04505	1	3	3	5	10	2	4	3	4	2	3	1	0	5	1	2	3

**Description**	**TreeFam ID**	**Pfam ID**	***G.a.***	***A.c.***	***P.s.***	***H.s.***	***M.m.***	***O.a.***	***G.g.***	***A.p.***	***M.g***	***T.g.***	***F.a.***	***A.cs.***	***T.a.***	***A.m.***	***S.c.***	***T.gt.***	***A.m.****
von Willebrand factor type D domain	TF336561	PF00094	3	4	3	1	2	4	2	3	2	1	2	1	1	5	1	1	2
PDZ domain (Also known as DHR or GLGF)	TF323171	PF00595	13	6	8	6	5	13	6	5	8	8	6	6	6	10	6	6	7
PDZ domain	TF330709	PF00595	9	5	7	4	4	12	5	5	4	8	5	4	5	10	5	4	7
Est1 DNA/RNA binding domain	TF327119	PF10373	3	5	3	3	3	6	3	3	6	5	4	2	2	6	3	3	4
von Willebrand factor type A domain	TF329914	PF00092	6	5	4	3	3	10	3	3	3	6	3	4	3	7	3	4	5
Helicase associated domain (HA2)	TF318311	PF04408	2	1	1	1	1	4	1	1	1	7	2	2	3	3	1	1	3
Immunoglobulin C1-set domain	TF334274	PF07654	10	1	5	8	2	1	2	1	2	0	0	1	1	3	1	1	3
no description	TF336669	no hits	0	0	0	0	0	4	12	2	7	0	0	0	0	3	1	1	3
Function to find	TF352798	PF13553	0	1	7	1	0	0	1	0	2	1	1	1	1	3	1	1	3
Vault protein inter-alpha-trypsin domain	TF328982	PF08487	7	7	6	6	5	13	4	6	5	5	4	3	2	8	4	4	5
O-Glycosyl hydrolase family 30	TF314254	PF02055	1	4	0	2	1	0	3	3	0	3	2	1	2	4	1	3	3

Description	TreeFam ID	Pfam ID	*G.a.*	*A.c.*	*P.s.*	*H.s.*	*M.m.*	*O.a.*	*G.g.*	*A.p.*	*M.g*	*T.g.*	*F.a.*	*A.cs.*	*T.a.*	*A.m.*	*S.c.*	*T.gt.*	*A.m.**
Flagellar-associated PapD-like	TF328687	PF14874	1	1	2	4	1	6	1	2	1	6	4	0	2	4	1	1	3
Alpha-2-Macroglobulin	TF335433	PF00207	1	1	2	11	1	1	2	3	2	1	2	1	1	4	1	1	4
NACHT domain	TF340267	PF05729	0	4	8	33	20	8	2	2	2	2	2	2	2	4	2	2	4
Kunitz/Bovine pancreatic trypsin inhibitor domain	TF315349	PF00014	6	11	5	4	3	2	3	4	4	2	4	1	2	5	2	3	5
Acetyltransferase (GNAT) family	TF324687	PF00583	4	3	3	2	10	5	1	1	2	0	1	1	1	2	1	1	2
DDE superfamily endonuclease	TF327972	PF13359	30	2	3	1	1	1	1	1	1	2	2	1	1	2	1	1	2
SET domain containing 1A/1B	TF106436	PF11764	3	2	4	2	2	4	1	1	1	5	1	1	2	3	1	1	3
Glycosyltransferase sugar-binding region containing DXD motif	TF324053	PF04488	0	2	15	2	2	2	2	2	2	4	3	3	3	3	2	2	3
Homeobox domain	TF351530	PF00046	7	3	4	3	3	2	4	2	2	3	5	1	1	3	1	1	3
Transforming growth factor beta superfam.	TF351791	PF00019	5	3	1	4	4	1	2	3	3	2	4	1	2	3	1	1	3

Description	TreeFam ID	Pfam ID	*G.a.*	*A.c.*	*P.s.*	*H.s.*	*M.m.*	*O.a.*	*G.g.*	*A.p.*	*M.g*	*T.g.*	*F.a.*	*A.cs.*	*T.a.*	*A.m.*	*S.c.*	*T.gt.*	*A.m.**
Homeobox domain	TF315976	PF00046	4	3	3	10	6	3	1	4	1	2	1	3	1	4	2	3	4
Rhodopsin-like receptors	TF341723	PF00001	7	11	25	11	12	8	2	4	4	3	4	3	3	4	2	3	4

Families contracted in *Apteryx mantelli*

Description	TreeFam ID	Pfam ID	*G.a.*	*A.c.*	*P.s.*	*H.s.*	*M.m.*	*O.a.*	*G.g.*	*A.p.*	*M.g*	*T.g.*	*F.a.*	*A.cs.*	*T.a.*	*A.m.*	*S.c.*	*T.gt.*
Cysteine-rich secretory proteins, antigen 5, and pathogenesis-related 1 proteins (CAP)	TF350472	PF00188	3	7	6	9	6	6	5	3	4	4	5	2	3	1	4	4
BTB/POZ domain	TF330633	PF00651	19	20	11	15	12	16	15	14	14	17	14	10	13	7	13	12
Kazal-type serine protease inhibitor domain	TF352550	PF00050	0	4	8	4	7	5	4	5	4	9	8	6	6	2	6	5
Ubiquitin-conjugating_enzyme_E2_E	TF101117	PF00179	11	5	5	7	10	8	4	7	5	9	5	5	6	3	6	6
Zinc finger, C4 type (two domains)	TF352167	PF00105	15	15	11	13	14	8	10	8	7	13	10	8	8	4	7	8
centromere_protein_F	TF101133	PF10473	2	1	2	1	1	1	1	1	1	4	1	4	3	1	3	4
Immunoglobulin C1-set domain	TF336715	PF07654	1	2	2	50	12	6	4	3	2	2	2	3	3	1	3	2
Pyridoxal-phosphate dependent	TF300784	PF00291	1	2	2	1	1	1	2	4	1	6	4	5	4	2	4	4

enzyme																		

Description	TreeFam ID	Pfam ID	*G.a.*	*A.c.*	*P.s.*	*H.s.*	*M.m.*	*O.a.*	*G.g.*	*A.p.*	*M.g*	*T.g.*	*F.a.*	*A.cs.*	*T.a.*	*A.m.*	*S.c.*	*T.gt.*
Coiled-coil serine-rich protein 2	TF331021	no hit	2	2	3	2	2	2	2	3	2	7	3	4	5	2	5	4
Homeobox domain	TF352857	PF00046	10	8	10	9	8	7	14	9	3	7	4	4	5	4	6	7
Histone	TF332276	PF00125	25	15	16	31	39	37	14	16	7	15	7	10	5	10	12	14
Mouse development and cellular proliferation protein Cullin-7	TF322454	PF11515	0	1	2	0	0	1	0	1	2	2	1	1	0	0	1	0
Cor1/Xlr/Xmr conserved region	TF328876	PF04803	0	0	1	4	83	2	1	1	1	1	1	1	0	0	1	1
Drug resistance and apoptosis regulator	TF336906	PF15017	0	0	1	1	1	0	1	0	0	0	0	1	0	0	1	0
SH3 domain	TF337296	PF00018	0	0	0	2	1	2	0	0	0	0	0	2	0	0	1	0
Immunoglobulin V-set domain	TF337790	PF07686	0	0	1	8	0	0	1	1	0	1	1	1	1	0	1	1
Dynein, axonemal, heavy chain 14	TF342240	no hit	0	0	0	1	0	1	1	1	0	0	0	1	0	0	1	0
Translation initiation factor 1A / IF-1	TF350394	PF01176	1	1	1	2	18	1	1	1	1	1	1	1	1	0	1	1
Zinc-finger double domain	TF350840	PF13465	0	0	0	2	1	0	0	0	0	0	0	2	0	0	1	0
CD59 antigen	TF352648	PF00021	0	15	6	1	1	1	1	1	1	1	1	1	1	0	1	1

Description	TreeFam ID	Pfam ID	*G.a.*	*A.c.*	*P.s.*	*H.s.*	*M.m.*	*O.a.*	*G.g.*	*A.p.*	*M.g*	*T.g.*	*F.a.*	*A.cs.*	*T.a.*	*A.m.*	*S.c.*	*T.gt.*
Glutathione S-transferase, N-terminal domain	TF353040	PF02798	2	4	3	6	13	1	0	0	0	0	1	1	0	0	1	1
CLASP N terminal	TF315518	PF12348	1	2	2	2	2	2	1	3	2	22	4	1	3	4	3	2

Supplementary Table S7. Annotated membrane proteome in *Apteryx mantelli*, *Homo sapiens,* and birds and reptiles. Significantly * = contracted, ** = expanded family in *Apteryx mantelli* in comparison to birds and reptiles ($p < 0.05$) (shown in bold), calculated using the Viterbi algorithm implemented in CAFE [2] with an estimated gene birth and death parameter of 0.000855882.

H.s = Homo sapiens, A.m. = Apteryx mantelli, G.g. = Gallus gallus, A.p. = Anas platyrhynchos, A.c. = Anolis carolinensis, A.cs. = Antrostomus carolinensis, F.a = Ficedula albicollis, M.g. = Meleagris gallopavo, P.s. = Pelodiscus sinensis, S.c. = Struthio camelus, T.g. = Taeniopygia guttata, T.gt. = Tinamus guttatus, T.a. = Tyto alba.

§ = Family which showed initially expansion in *Apteryx mantelli* (initially predicted number of genes/number of genes after manual curation).

**Category**	***H.s***	***A.m.***	***G.g.***	***A.p.***	***A.c.***	***A.cs.***	***F.a.***	***M.g.***	***P.s.***	***S.c.***	***T.g.***	***T.gt.***	***T.a.***
Classified	2,995	2,011	2,046	2,024	2,416	1,709	2,002	1,901	2,525	1,681	2,185	1,917	1,576
Unclassified	2,741	1,984	1,886	1,670	2,112	1,240	1,787	1,616	1,889	1,310	1,654	1,340	1,216
Total no predicted TM proteins (Phobius)	5,736	3,995	3,932	3,694	4,528	2,949	3,789	3,517	4,414	2,991	3,839	3,257	2,792
Total no of genes	20,406	18,033	15,508	15,634	18,596	14,676	15,303	14,125	18,188	16,178	17,488	15,773	13,613

**Class/Family**	***H.s***	***A.m.***	***G.g.***	***A.p.***	***A.c.***	***A.cs.***	***F.a.***	***M.g.***	***P.s.***	***S.c.***	***T.g.***	***T.gt.***	***T.a.***
**Enzymes**													
1	2	2	2	2	1	2	2	2	2	1	1	2	1
1.1	10	4	8	8	9	7	9	9	9	6	11	7	6
1.11	1	1	0	1	1	0	0	0	1	0	0	1	0
1.14	2	0	1	1	2	1	1	0	1	1	0	0	1
3.4	5	0	1	0	0	0	0	0	0	0	0	0	0
1.1.1.145/5.3.3.1	2	1	1	1	1	0	1	0	1	0	1	0	0
1.14.11	1	0	2	1	1	0	1	1	1	1	1	0	0
1.14.13	11	6	7	5	12	3	7	7	7	4	5	5	4
1.14.14	14	4	7	6	11	3	7	7	11	4	5	4	4
1.14.15	1	0	0	0	2	0	0	0	0	0	0	0	0

**Class/Family**	***H.s***	***A.m.***	***G.g.***	***A.p.***	***A.c.***	***A.cs.***	***F.a.***	***M.g.***	***P.s.***	***S.c.***	***T.g.***	***T.gt.***	***T.a.***
1.14.17	1	2	1	1	1	0	1	1	1	0	1	0	0
1.14.17.3/4.3.2.5	1	0	1	1	1	0	1	1	1	1	1	1	1
1.14.18	1	1	1	1	1	1	1	1	1	1	1	1	1
1.14.19	1	1	1	1	1	1	1	1	1	1	1	1	1
1.14.21	1	1	1	1	1	0	1	1	1	1	1	1	0
1.14.99	4	3	4	4	3	4	3	3	4	4	4	4	3
1.17.4	1	1	1	1	1	1	1	1	1	1	1	1	1
1.2.1	2	1	1	1	1	1	1	2	1	1	1	1	0
1.3.1	2	3	1	1	2	1	1	1	2	1	1	1	1
1.3.3	2	0	1	0	0	0	0	0	0	0	0	0	0
1.3.99	2	2	3	3	3	3	3	1	3	3	3	2	3
1.4.3	2	1	2	2	2	2	2	2	2	2	1	2	2
1.5.1	1	1	1	1	1	1	1	1	1	1	1	1	1
1.5.3	1	0	0	0	1	0	0	0	0	0	0	0	0
1.5.5	1	0	0	1	1	1	1	0	1	1	1	0	0
1.6.1	1	2	1	1	2	1	1	1	2	1	1	2	1
1.6.2	1	1	1	1	0	2	0	1	1	2	1	0	2
1.6.3	5	4	4	3	4	3	3	4	3	3	3	3	3
**1.6.5***	**15**	**2**	**13**	**6**	**14**	**3**	**13**	**13**	**9**	**4**	**15**	**9**	**8**
1.8.3	2	0	2	1	1	1	2	1	1	1	2	1	1
**1.9.3***	**13**	**1**	**6**	**1**	**6**	**2**	**6**	**5**	**7**	**3**	**6**	**2**	**3**
1.97.1	2	0	2	2	2	2	2	1	2	2	3	2	2
2.1.1	2	2	2	2	1	1	2	1	2	2	2	2	2
2.3.1	57	37	42	42	52	34	42	37	46	36	43	35	30
2.4.1	64	31	37	37	40	22	37	31	37	26	35	24	21
2.4.2	5	2	2	1	4	1	3	2	3	1	2	1	1
2.4.99	4	0	1	1	2	0	1	0	0	0	1	1	0
2.5.1	4	3	3	3	6	3	3	3	4	4	3	3	3
2.7.1	4	0	1	1	1	1	0	1	2	0	1	0	0
2.7.10	1	0	1	1	0	0	1	1	1	1	1	0	1
2.7.11	20	11	11	11	15	7	12	11	9	10	13	11	7
2.7.7	2	2	2	2	2	2	2	2	2	2	2	2	2
2.7.8	8	7	8	9	10	6	8	7	7	8	9	8	7

Class/Family	*H.s*	*A.m.*	*G.g.*	*A.p.*	*A.c.*	*A.cs.*	*F.a.*	*M.g.*	*P.s.*	*S.c.*	*T.g.*	*T.gt.*	*T.a.*
2.8.2	12	2	8	5	6	4	6	6	6	5	7	4	3
3.1.1	6	4	2	4	9	1	2	3	6	2	1	2	2
3.1.11	1	0	0	0	0	0	0	0	0	0	0	0	0
3.1.2	3	1	2	0	0	1	1	1	1	2	2	1	0
3.1.3	34	24	24	20	32	16	24	24	22	18	27	19	18
3.1.4	12	4	5	5	9	3	8	5	8	4	5	3	4
3.1.4.1/3.6.1.9	2	1	2	2	2	0	1	1	1	0	2	0	0
3.1.6	5	3	4	2	4	4	3	3	3	4	3	4	4
3.2.1	16	6	11	8	11	1	10	5	11	6	7	3	4
3.2.2	1	1	0	0	0	0	0	0	1	1	0	0	0
3.4.	1	1	1	1	1	0	1	1	0	1	0	0	0
3.4.11	3	2	3	3	3	2	3	1	2	2	2	2	1
3.4.15	1	1	1	1	1	2	1	1	1	2	1	2	2
3.4.17	3	1	3	3	1	2	2	2	3	2	3	0	2
3.4.19	1	0	0	0	0	0	1	0	0	0	0	0	0
3.4.21	17	8	13	12	11	8	13	13	11	6	10	6	5
3.4.23	2	2	2	2	2	1	2	2	1	1	2	1	1
3.4.24	36	22	30	27	32	11	26	28	24	18	20	17	16
3.5.1	3	3	3	3	1	1	3	2	3	2	1	2	2
3.6.1§	9	12/11	11	11	9	7	10	9	8	8	10	6	4
4.1.2	1	1	0	0	1	1	0	0	1	1	0	1	0
4.2.1	4	3	4	2	2	2	3	4	4	1	1	1	1
4.4.1	1	1	2	2	1	2	2	2	2	1	2	1	2
4.6.1	10	12	8	8	11	8	7	9	11	10	9	13	8
5.1.3	1	1	1	1	1	1	1	1	1	1	1	1	1
5.2.1	2	1	1	0	1	0	2	0	2	1	2	1	0
5.3.3	2	1	1	1	2	0	1	1	2	1	1	1	0
5.3.4	1	1	0	0	0	0	0	0	1	1	0	0	0
5.3.99	2	1	2	2	2	2	2	2	2	1	2	0	1
6.2.1	5	3	4	5	5	3	4	5	4	5	5	4	3
6.3.2	2	2	2	2	2	1	2	2	1	2	2	2	2
**Miscellaneous**													
Ligand Delta	5	3	4	4	5	4	4	4	4	3	4	4	3

Class/Family	*H.s*	*A.m.*	*G.g.*	*A.p.*	*A.c.*	*A.cs.*	*F.a.*	*M.g.*	*P.s.*	*S.c.*	*T.g.*	*T.gt.*	*T.a.*
Ligand EphB	3	1	2	1	3	1	2	2	3	2	2	2	1
Ligand IG Ligand	2	2	4	2	1	2	3	3	2	2	2	2	2
Ligand Jagged	2	2	2	2	2	0	2	2	2	2	2	2	1
**Ligand MHC****	**18**	**4/4**	**9**	**3**	**9**	**1**	**4**	**4**	**4**	**0**	**2**	**0**	**0**
Ligand Neuroligin	5	4	3	3	4	3	3	3	3	3	2	2	2
Ligand NKG2DL	4	0	0	0	0	0	0	0	0	0	0	0	0
Ligand Semaphorins	11	6	8	6	10	3	8	8	8	6	7	5	3
Butyrophylin	15	4	12	12	4	2	5	9	51	3	1	4	4
CMTM	9	5	6	6	6	5	5	4	5	5	6	5	5
DnaJ	11	6	7	6	11	5	7	6	9	4	7	7	6
DPY19	4	3	3	3	3	3	3	3	3	3	4	3	3
Ferlin	4	4	6	5	4	3	4	7	3	3	4	2	3
GBP	7	2	4	2	3	0	2	5	3	2	2	2	1
HERV1	2	0	0	0	8	4	0	0	0	0	1	2	7
IFITM	4	2	4	2	3	0	3	3	2	0	2	1	0
LASS	9	11	10	10	7	9	7	9	9	9	8	8	9
LRRC	64	44	56	43	57	38	52	44	55	34	40	39	30
LRRC8	5	4	4	4	5	4	4	4	5	4	4	4	4
MAL	5	5	4	5	7	4	4	5	7	3	4	5	3
REEP	4	1	4	4	4	5	5	4	5	5	5	4	3
RTP	4	1	0	0	0	0	0	0	1	0	0	0	0
SCAMP	4	4	5	5	4	5	5	2	5	5	5	4	4
SIRP	3	0	0	1	2	0	1	2	2	0	2	0	0
Synaptogyrin	4	3	3	2	4	1	3	1	3	1	2	1	1
Synaptophysin	4	2	3	3	5	3	3	2	4	2	4	3	2
Synaptotagmin	14	9	11	9	12	10	11	10	12	9	11	10	10
**Teneurin***	**4**	**0**	**3**	**5**	**3**	**1**	**4**	**4**	**3**	**4**	**3**	**3**	**1**
Tetraspanin	32	24	27	26	35	21	29	25	31	22	27	23	20
TMED	8	6	7	8	8	4	7	6	10	5	4	5	5
Structural and Adhesion CadherinClassic	22	16	19	19	21	25	20	18	19	18	14	19	13
Structural and Adhesion Cadherin	7	0	3	3	3	3	3	2	4	3	2	3	3
Structural and Adhesion Calsyntenin	3	2	3	3	3	2	3	3	3	3	3	3	2

Class/Family	*H.s*	*A.m.*	*G.g.*	*A.p.*	*A.c.*	*A.cs.*	*F.a.*	*M.g.*	*P.s.*	*S.c.*	*T.g.*	*T.gt.*	*T.a.*
Structural and Adhesion Claudin§	23	16/15	18	20	24	13	19	17	19	11	16	10	13
Structural and Adhesion Crumbs Protein	3	2	2	2	2	1	2	2	2	2	3	1	1
Structural and Adhesion EMP-PMP22-LIM	5	3	3	3	6	3	3	3	6	3	2	3	2
Structural and Adhesion GapJunction	20	18	16	18	18	16	20	14	17	15	20	16	13
Structural and Adhesion IG Adhesion Proteins	43	12	17	17	22	14	16	15	18	14	15	28	10
Structural and Adhesion IG MPZ	4	3	3	3	2	2	3	3	4	3	3	2	3
**Structural and Adhesion Junctophilin****	**4**	**2/2**	**2**	**3**	**3**	**0**	**2**	**3**	**3**	**0**	**3**	**0**	**0**
**Structural and Adhesion Protocadherins Beta****	**15**	**2/2**	**3**	**1**	**15**	**0**	**4**	**1**	**3**	**1**	**1**	**0**	**0**
**Structural and Adhesion Protocadherins***	**23**	**22**	**24**	**20**	**24**	**29**	**24**	**25**	**21**	**50**	**18**	**42**	**8**
Structural and Adhesion Sarcoglycan	6	2	6	4	6	4	4	2	5	4	5	3	4
Structural and Adhesion UPK3	2	1	2	1	0	0	1	2	3	0	0	0	0
ARMC	6	0	1	0	1	0	0	0	0	0	0	0	0
CNIH	4	2	2	3	4	1	3	3	3	2	3	3	2
**CSMD***	**3**	**4**	**3**	**3**	**3**	**6**	**4**	**2**	**3**	**15**	**3**	**16**	**0**
cTAGE	3	1	2	0	0	1	1	0	0	0	1	0	1
DC2	1	1	1	1	1	1	1	1	1	1	1	1	1
ELOVL	6	6	6	7	5	5	7	5	8	4	6	8	6
FAM163	3	2	2	2	2	1	2	2	2	1	2	1	0
FAM74	1	0	0	0	0	0	0	0	0	0	0	0	0
FAM75	8	0	0	0	0	0	0	0	0	0	0	0	0
HIGD	4	2	2	2	3	2	2	2	1	1	2	1	2
**IG Unknown function****	**9**	**5/5**	**5**	**3**	**5**	**3**	**5**	**4**	**7**	**3**	**3**	**1**	**1**
ITM2	3	1	3	3	3	3	3	3	2	3	3	3	3
LHFP	6	5	4	5	5	4	5	3	7	3	6	5	2
LRRC37	4	0	0	0	1	0	0	0	1	0	0	0	0
MS4A	14	0	1	1	5	0	2	1	7	1	1	1	0
NTMG1	1	0	0	0	0	4	1	0	0	0	1	1	0

**Class/Family**	***H.s***	***A.m.***	***G.g.***	***A.p.***	***A.c.***	***A.cs.***	***F.a.***	***M.g.***	***P.s.***	***S.c.***	***T.g.***	***T.gt.***	***T.a.***
ORM1DL	3	3	3	2	3	2	2	2	3	3	2	3	2
Reticulon	4	3	2	2	4	0	2	2	3	3	2	1	1
RNFT	2	2	2	2	2	2	2	2	2	2	2	2	2
TetraspaninL6	6	1	5	5	5	5	5	5	6	5	5	5	4
TM9SF	4	6	4	4	5	4	4	4	4	4	5	4	4
TMEM132	5	4	4	4	5	4	5	4	4	3	4	4	3
TMEM16	9	11	8	9	10	8	8	8	10	9	8	9	7
TMEM30	2	2	2	2	2	2	2	2	2	2	2	2	2
TMEM63	3	4	3	3	3	3	3	3	3	3	3	3	3
**Receptors**													
GPCR Adhesion	30	27	23	22	22	19	22	21	31	35	20	35	15
GPCR Frizzled	11	13	9	11	10	9	11	10	8	8	12	10	6
GPCR Glutamate§	22	19/18	17	17	69	13	17	18	20	14	16	14	11
GPCR Olf	366	82	30	77	89	85	39	43	282	26	234	138	82
GPCR Other	5	3	3	3	4	4	3	3	3	3	4	3	3
GPCR PutativeOlfR	7	0	1	0	0	0	0	0	17	0	0	1	0
GPCR Rhodopsin	280	254	254	271	298	209	251	229	323	163	247	202	198
GPCR Secretin	14	16	15	14	15	13	14	15	14	12	20	15	13
GPCR TAS2R	24	0	1	3	3	2	3	1	0	1	3	1	1
GPCR V1R	3	0	0	0	0	0	0	0	0	0	0	0	0
IG FcR	13	1	2	2	0	1	2	1	1	1	1	0	1
IG KIR	6	0	0	0	0	0	0	0	0	0	0	0	0
IG LILR	10	1	7	0	2	0	0	0	2	0	0	0	0
IG NetrinR	12	10	10	9	10	9	9	10	10	7	10	10	9
**IG CD1****	**4**	**2/2**	**2**	**5**	**1**	**0**	**1**	**1**	**1**	**0**	**1**	**0**	**0**
IG CD300	6	1	2	1	1	0	1	3	3	0	0	0	0
**IG Misc****	**6**	**6/6**	**4**	**2**	**3**	**1**	**3**	**2**	**4**	**1**	**1**	**4**	**1**
IG NCR	3	0	1	0	0	0	0	0	1	0	0	0	0
IG Other	8	4	4	3	6	4	5	3	6	6	4	4	4
**IG PVR****	**5**	**2/2**	**2**	**2**	**3**	**0**	**2**	**1**	**4**	**0**	**2**	**0**	**0**
**IG ROBO***	**4**	**2**	**4**	**3**	**4**	**8**	**4**	**4**	**4**	**8**	**2**	**4**	**8**
IG TREM	6	1	1	1	1	1	1	2	2	1	1	1	1
IG TCR	2	0	0	0	1	0	0	1	0	0	0	0	0

**Class/Family**	***H.s***	***A.m.***	***G.g.***	***A.p.***	***A.c.***	***A.cs.***	***F.a.***	***M.g.***	***P.s.***	***S.c.***	***T.g.***	***T.gt.***	***T.a.***
IG Type1 CytokineR	40	21	34	30	26	22	33	25	30	23	26	26	19
IG Type2 CytokineR	10	5	9	10	7	5	7	10	9	4	5	5	4
Kinase Act.TGFB	12	10	12	10	12	7	11	11	12	8	11	10	7
Kinase Axl	3	2	2	2	3	1	2	2	2	2	2	1	2
Kinase EGFR	4	2	3	3	4	1	3	4	3	2	3	2	2
**Kinase Eph****	**14**	**14/12**	**13**	**13**	**14**	**2**	**13**	**13**	**13**	**5**	**11**	**6**	**2**
Kinase FGFR	4	4	4	4	3	2	4	3	3	6	5	3	2
Kinase InsR	3	3	3	2	2	2	3	3	3	2	2	2	2
Kinase neutrophin	3	2	3	2	3	1	2	3	3	2	3	2	1
Kinase Other	17	16	16	15	16	16	17	16	16	17	16	18	14
Kinase PDGFR	5	3	5	5	5	4	5	5	5	5	6	4	5
Kinase RGC	6	5	5	5	5	3	5	3	4	5	5	3	3
ADIPO-PAQR	11	9	8	9	9	9	6	8	10	8	8	8	8
Contactin Ass. Prot.	7	5	4	3	4	2	4	4	4	3	3	3	1
Derlin	3	2	3	3	2	3	3	2	2	3	5	3	3
IL17	5	0	3	2	5	2	3	3	2	2	3	2	2
Integrin	26	12	15	16	25	12	17	16	26	13	14	13	8
KDELR	3	2	2	2	3	2	2	2	3	2	3	2	2
**LDLR***	**15**	**10**	**13**	**13**	**14**	**26**	**14**	**12**	**12**	**22**	**16**	**22**	**11**
Neurexin	3	3	1	2	3	1	2	2	3	1	2	1	1
Neuropilin	2	1	2	2	2	0	2	2	2	1	2	1	1
Notch	4	1	2	2	3	0	2	2	2	0	2	0	0
Patched	2	4	2	2	2	2	2	2	1	3	2	5	2
Plexin	9	7	7	7	9	6	7	6	7	7	7	7	4
RAMP	3	1	3	3	3	1	3	3	3	1	2	1	1
Receptor Type Phosphatases	20	13	20	17	21	16	19	19	14	20	20	22	15
Selectin	3	2	2	2	1	0	2	2	1	1	2	1	3
Syndecan	3	2	2	3	3	3	3	3	3	3	2	3	3
TNFNGF	26	8	19	16	16	11	18	16	17	9	12	9	9
TOLL	10	11	10	10	15	4	11	8	10	8	9	8	5
Transferrin	2	1	1	1	0	1	1	1	2	1	1	1	1
VPS10R	4	4	3	3	4	2	2	3	3	4	4	4	3
SCAR Class A	3	2	2	2	2	2	1	2	1	2	2	1	2

**Class/Family**	***H.s***	***A.m.***	***G.g.***	***A.p.***	***A.c.***	***A.cs.***	***F.a.***	***M.g.***	***P.s.***	***S.c.***	***T.g.***	***T.gt.***	***T.a.***
SCAR Class B	3	2	3	3	3	3	3	3	3	3	3	2	3
SCAR Class D	4	4	3	2	2	2	3	3	4	2	2	2	2
SCAR Class E	38	2	9	7	16	3	6	2	25	3	4	3	3
SCAR Class F	5	3	5	3	5	4	5	3	5	5	4	4	5
SCAR Class G	1	0	0	0	0	0	0	0	0	0	0	0	0
SCAR Class H	2	2	1	2	2	2	2	1	2	2	1	1	0
**SCAR Macrophage Mannose R***	**5**	**2**	**7**	**6**	**5**	**8**	**8**	**4**	**6**	**7**	**5**	**7**	**4**
**Transporters**													
Active transporters ABCA	12	14	10	10	10	16	10	10	9	10	13	12	16
Active transporters ABCB	11	12	9	9	12	7	9	8	7	12	8	13	8
Active transporters ABCC	12	15	11	11	12	12	10	11	13	12	14	13	13
Active transporters ABCD	4	2	3	3	4	3	3	3	3	3	3	3	3
Active transporters ABCG	5	8	5	6	7	6	6	5	8	6	8	6	5
Active transporters P-ATPase§	36	43/35	30	34	45	32	31	31	35	33	39	36	34
Auxiliary Transport Unit ATP1B	5	2	4	4	4	4	4	4	3	4	5	4	3
Auxiliary Transport Unit CACNA2D	5	3	3	3	2	3	4	4	4	3	3	2	2
Auxiliary Transport Unit CACNG	9	6	9	6	8	6	6	5	8	5	7	6	4
Auxiliary Transport Unit KCNE	5	3	4	5	4	3	4	4	4	3	3	3	3
Auxiliary Transport Unit KCNMB	4	3	3	3	3	2	3	3	3	2	3	2	2
Auxiliary Transport Unit NKAIN	4	5	4	4	2	5	4	4	4	3	4	4	4
Auxiliary Transport Unit PKD1	4	7	3	3	5	3	3	3	4	3	2	3	3
Auxiliary Transport Unit Sodium Channel Beta	3	3	3	3	4	3	3	2	3	2	3	3	2
Aquaporins	13	9	9	9	13	7	10	9	12	7	8	9	7
Chloride channels CLC	9	9	9	10	9	6	8	11	10	7	8	9	7
Chloride channels Tweety	3	2	2	1	3	2	2	2	2	1	2	1	1
Ligand gated ATP gated ion channels	7	4	7	7	5	4	5	7	6	5	6	5	5
Ligand gated ion channels cys-loop	47	38	43	40	40	34	42	41	44	34	43	39	39
Ligand gated Glutamate ion channels§	18	27/18	17	18	21	15	17	19	17	17	17	16	15
Voltage gated Ca Activated Potassium Channels	8	6	6	7	7	4	5	6	8	5	6	7	2
Voltage gated Calcium Channels§	10	22/11	8	10	13	15	8	8	11	17	9	34	18
Voltage gated catSper-Two-P	6	4	3	3	7	4	3	3	7	4	4	5	4

Class/Family	*H.s*	*A.m.*	*G.g.*	*A.p.*	*A.c.*	*A.cs.*	*F.a.*	*M.g.*	*P.s.*	*S.c.*	*T.g.*	*T.gt.*	*T.a.*
Voltage gated cyclic Nucleotide Regulated Channels	10	9	10	10	8	6	9	8	8	8	9	8	7
Voltage gated inwardly Rectifying K channel	15	15	14	15	16	13	16	15	15	13	13	13	11
Voltage gated Potassium channels	40	38	36	38	42	32	38	35	40	33	35	34	27
Voltage gated RYR ITPR	6	7	5	5	6	4	5	6	5	4	6	5	5
Voltage gated Sodium Channels§	10	20/11	9	10	10	29	9	10	10	17	11	17	28
Voltage gated TRP channels§	27	33/27	25	25	27	21	24	25	29	20	30	27	23
Voltage gated Two-p K channels	15	14	13	13	16	12	12	11	14	12	12	13	12
BCL2	4	1	3	2	1	1	2	3	3	2	3	2	1
Bestrophin	4	3	3	3	4	3	2	3	2	3	4	3	3
SERINC	5	8	5	5	5	4	5	5	4	4	5	6	3
Sidoreflexins	5	0	5	4	5	3	5	5	4	3	5	3	4
STEAP	5	3	4	4	4	4	4	3	4	4	6	5	4
Syntaxin	12	1	7	7	12	4	6	7	11	2	9	3	3
VAMP	6	0	4	4	5	3	4	4	7	3	4	3	3
XK	8	6	7	8	6	4	8	8	8	4	7	6	4
APC SLC12	9	11	8	8	14	7	8	8	6	7	8	9	6
APC SLC23	3	4	4	4	4	4	4	4	3	4	5	3	4
APC SLC26	11	14	11	11	11	10	9	11	11	10	13	9	10
APC SLC32	1	1	2	2	2	2	2	2	2	2	2	2	2
APC SLC36	4	2	2	2	2	2	2	2	3	2	4	2	3
APC SLC38	11	14	11	10	12	8	11	13	12	11	12	11	9
APC SLC4§	10	16/9	10	9	12	12	10	11	9	6	11	9	7
APC SLC5§	12	16/13	10	11	12	8	9	12	14	7	11	9	9
APC SLC7	13	18	16	15	17	12	15	15	16	11	19	14	13
DMT AMAC	5	1	1	1	2	1	1	1	2	1	1	1	1
DMT NIPA	6	8	7	7	7	8	7	7	7	7	8	7	7
DMT SLC35	23	23	20	22	22	20	22	18	19	22	24	20	20
MFS HIAT	4	3	3	4	3	4	4	3	4	5	4	4	5
MFS SLC15	4	5	3	3	4	4	3	3	4	3	3	4	4
MFS SLC16	14	15	16	14	15	13	14	14	15	12	15	13	13
MFS SLC17	9	7	4	5	5	4	4	4	6	4	6	4	5

Class/Family	*H.s*	*A.m.*	*G.g.*	*A.p.*	*A.c.*	*A.cs.*	*F.a.*	*M.g.*	*P.s.*	*S.c.*	*T.g.*	*T.gt.*	*T.a.*
MFS SLC18	6	9	5	6	7	5	6	6	6	5	6	5	3
MFS SLC19	3	4	4	4	3	4	4	3	2	3	6	4	4
MFS SLC2	14	18	15	17	18	15	16	15	18	16	19	13	16
MFS SLC22	22	17	14	17	24	16	14	15	21	12	15	12	11
MFS SLC29	4	4	3	3	3	3	3	3	4	3	3	3	2
MFS SLC37	3	3	4	4	4	2	3	4	3	3	4	4	3
MFS SLC43	3	2	2	2	3	1	2	2	3	1	3	1	1
MFS SLC45	4	6	5	4	4	4	4	4	4	4	5	5	4
MFS SLC46	3	3	3	3	3	3	3	3	3	2	3	3	3
MFS SLCO	11	13	10	11	11	8	10	9	11	8	14	10	8
MFS Spinster	3	3	2	2	2	2	2	2	2	1	2	1	1
MFS SV2	3	4	3	3	2	2	2	2	3	2	2	3	1
Mitochondrial carrier SLC25	11	6	6	5	11	5	7	6	8	5	7	4	5
SLC1	7	7	7	7	7	6	7	9	9	5	9	6	3
SLC10	7	5	4	5	5	2	4	4	6	1	4	2	2
SLC11	2	2	1	2	2	0	2	1	2	2	1	2	1
SLC13	5	5	5	5	5	5	5	6	6	4	5	5	5
SLC14	2	2	1	1	1	4	1	3	1	2	1	4	3
SLC20	2	2	2	2	1	2	2	2	2	2	2	2	2
SLC24	6	8	6	6	5	5	6	6	6	6	7	6	5
SLC27	6	1	2	3	4	2	3	1	3	1	1	1	0
SLC28	3	3	2	2	2	2	2	2	2	2	2	2	2
SLC3	2	1	1	1	2	1	1	1	2	1	1	1	1
SLC30	10	8	9	9	11	9	10	9	11	8	11	8	8
SLC31	2	4	1	2	1	2	2	2	1	2	2	2	1
SLC33	1	1	1	1	1	1	1	1	1	1	2	1	1
SLC34	3	3	2	2	2	1	2	2	1	2	3	3	1
SLC39	13	10	9	9	12	7	8	8	11	8	11	7	7
SLC40	1	1	1	1	1	1	1	1	2	1	1	1	1
SLC41	3	3	4	3	3	3	3	3	3	3	3	3	3
SLC42(Rh)	5	5	5	5	4	3	4	5	4	3	4	3	3
SLC44	5	6	4	4	5	3	4	3	5	3	4	4	2
SLC6[§]	20	25/21	20	18	21	18	19	21	17	15	23	17	18

Class/Family	*H.s*	*A.m.*	*G.g.*	*A.p.*	*A.c.*	*A.cs.*	*F.a.*	*M.g.*	*P.s.*	*S.c.*	*T.g.*	*T.gt.*	*T.a.*
SLC8	3	5	2	2	6	3	3	2	4	2	2	2	2
SLC9	11	10	11	10	9	9	10	10	10	9	11	9	8

Supplementary Table S8. Gene Ontology categories enriched for genes, which evolve faster in kiwi; (A) kiwi-specific and (B) shared with any of the other tested *Palaeognathae* (ostrich, tinamou) or nocturnal birds (chuck-will's-widow, barn owl). The enrichment was performed using a hypergeometric test [19] on genes evolving significantly faster in kiwi. The background species considered in CODEML [9] were chicken, turkey, zebra finch, ostrich, tinamou, chuck-will's-widow, and barn owl. (C) Genes belonging to the potentially biological relevant categories for kiwi's specific physiology. None of the categories remained significant after family-wise error rate multiple testing correction.

A)

Node name	Node ID	No. genes in node	No. significant genes	Raw p over representation
**Biological process**				
oxidation-reduction process	GO:0055114	637	43	0.001
protein targeting	GO:0006605	270	18	0.024
small molecule catabolic process	GO:0044282	124	10	0.027
single-organism catabolic process	GO:0044712	124	10	0.027
response to alcohol	GO:0097305	76	9	0.003
**visual perception**	**GO:0007601**	**96**	**9**	**0.015**
regulation of ERK1 and ERK2 cascade	GO:0070372	112	9	0.036
ERK1 and ERK2 cascade	GO:0070371	117	9	0.046
organic acid catabolic process	GO:0016054	95	8	0.037
regulation of leukocyte mediated immunity	GO:0002703	78	7	0.037
establishment of protein localization to organelle	GO:0072594	82	7	0.046
response to ethanol	GO:0045471	23	6	<0.001
histone ubiquitination	GO:0016574	32	6	0.002
protein targeting to membrane	GO:0006612	50	6	0.014
positive regulation of epithelial cell migration	GO:0010634	60	6	0.033
spermatid development	GO:0007286	63	6	0.040
endothelial cell proliferation	GO:0001935	64	6	0.043
spermatid differentiation	GO:0048515	65	6	0.046
negative regulation of TOR signaling cascade	GO:0032007	17	5	<0.001
ER-associated protein catabolic process	GO:0030433	30	5	0.006
peptidyl-proline modification	GO:0018208	38	5	0.017
regulation of TOR signaling cascade	GO:0032006	38	5	0.017
glycogen metabolic process	GO:0005977	43	5	0.028
cellular glucan metabolic process	GO:0006073	43	5	0.028
purine nucleoside triphosphate biosynthetic process	GO:0009145	43	5	0.028
glucan metabolic process	GO:0044042	43	5	0.028
negative regulation of angiogenesis	GO:0016525	45	5	0.033

Node name	Node ID	No. genes in node	No. significant genes	Raw p over representation
monocarboxylic acid catabolic process	GO:0072329	45	5	0.033
**energy reserve metabolic process**	**GO:0006112**	**47**	**5**	**0.039**
organophosphate ester transport	GO:0015748	47	5	0.039
TOR signaling cascade	GO:0031929	47	5	0.039
regulation of interleukin-6 production	GO:0032675	48	5	0.042
acrosome assembly	GO:0001675	9	4	<0.001
histone H2A ubiquitination	GO:0033522	17	4	0.004
amino sugar metabolic process	GO:0006040	22	4	0.011
fatty acid beta-oxidation	GO:0006635	25	4	0.017
regulation of smooth muscle contraction	GO:0006940	26	4	0.019
**feeding behavior**	**GO:0007631**	**40**	**4**	**0.021**
protein peptidyl-prolyl isomerization	GO:0000413	27	4	0.022
axon cargo transport	GO:0008088	27	4	0.022
RNA phosphodiester bond hydrolysis, endonucleolytic	GO:0090502	28	4	0.024
**eye photoreceptor cell differentiation**	**GO:0001754**	**30**	**4**	**0.031**
translational elongation	GO:0006414	31	4	0.034
ATP biosynthetic process	GO:0006754	31	4	0.034
protein autoubiquitination	GO:0051865	31	4	0.034
negative regulation of smooth muscle contraction	GO:0045986	7	3	0.002
negative regulation of muscle contraction	GO:0045932	9	3	0.005
regulation of translational fidelity	GO:0006450	10	3	0.006
histone H2A acetylation	GO:0043968	11	3	0.008
protein-chromophore linkage	GO:0018298	12	3	0.011
peptide hormone processing	GO:0016486	13	3	0.014
negative regulation of purine nucleotide metabolic process	GO:1900543	14	3	0.017
negative regulation of muscle tissue development	GO:1901862	14	3	0.017
regulation of translational elongation	GO:0006448	15	3	0.020
negative regulation of nucleotide metabolic process	GO:0045980	15	3	0.020
regulation of actin cytoskeleton reorganization	GO:2000249	15	3	0.020
nuclear envelope organization	GO:0006998	16	3	0.024
gamma-aminobutyric acid signaling pathway	GO:0007214	16	3	0.024
protein localization to vacuole	GO:0072665	17	3	0.029
synaptic transmission, dopaminergic	GO:0001963	18	3	0.033
histone monoubiquitination	GO:0010390	19	3	0.039
glycogen biosynthetic process	GO:0005978	21	3	0.050
glucan biosynthetic process	GO:0009250	21	3	0.050
positive regulation vascular endothelial growth factor production	GO:0010575	21	3	0.050

Node name	Node ID	No. genes in node	No. significant genes	Raw p over representation
**Cellular component**				
**mitochondrion**	**GO:0005739**	**1069**	**54**	**0.020**
vacuolar part	GO:0044437	158	14	0.003
lysosomal membrane	GO:0005765	126	10	0.023
histone acetyltransferase complex	GO:0000123	69	7	0.016
peroxisome	GO:0005777	77	7	0.028
microbody	GO:0042579	77	7	0.028
Cul5-RING ubiquitin ligase complex	GO:0031466	5	3	0.001
Swr1 complex	GO:0000812	7	3	0.002
costamere	GO:0043034	13	3	0.012
NuA4 histone acetyltransferase complex	GO:0035267	14	3	0.015
H4/H2A histone acetyltransferase complex	GO:0043189	15	3	0.018
INO80-type complex	GO:0097346	15	3	0.018
presynaptic membrane	GO:0042734	19	3	0.034
SAGA-type complex	GO:0070461	19	3	0.034
small nuclear ribonucleoprotein complex	GO:0030532	20	3	0.039
**Molecular function**				
oxidoreductase activity	GO:0016491	569	35	0.004
ligase activity	GO:0016874	337	23	0.006
ligase activity, forming carbon-nitrogen bonds	GO:0016879	259	19	0.005
acid-amino acid ligase activity	GO:0016881	232	18	0.004
small conjugating protein ligase activity	GO:0019787	212	17	0.003
ubiquitin-protein ligase activity	GO:0004842	191	16	0.003
protein complex binding	GO:0032403	244	15	0.049
magnesium ion binding	GO:0000287	129	12	0.004
isomerase activity	GO:0016853	100	10	0.005
hydrolase activity, acting on glycosyl bonds	GO:0016798	98	8	0.035
cation-transporting ATPase activity	GO:0019829	55	6	0.018
transmembrane receptor protein kinase activity	GO:0019199	62	6	0.031
drug binding	GO:0008144	33	5	0.008
methylated histone residue binding	GO:0035064	36	5	0.012
transmembrane receptor protein tyrosine kinase activity	GO:0004714	47	5	0.033
intramolecular transferase activity	GO:0016866	23	4	0.011
ATPase activity, coupled to transmembrane movement of ions	GO:0042625	55	4	0.018
cis-trans isomerase activity	GO:0016859	31	4	0.030
aminoacyl-tRNA editing activity	GO:0002161	8	3	0.003
GDP-dissociation inhibitor activity	GO:0005092	8	3	0.003
signal sequence binding	GO:0005048	12	3	0.010
thyroid hormone receptor binding	GO:0046966	15	3	0.018
hyaluronic acid binding	GO:0005540	17	3	0.026
GABA-A receptor activity	GO:0004890	18	3	0.030

Node name	Node ID	No. genes in node	No. significant genes	Raw p over representation
tumor necrosis factor receptor binding	GO:0005164	18	3	0.030
hydrolase activity, hydrolyzing N-glycosyl compounds	GO:0016799	19	3	0.035
neurotransmitter:sodium symporter activity	GO:0005328	20	3	0.039
GABA receptor activity	GO:0016917	20	3	0.039

B)

Node name	Node ID	No. genes in node	No. significant genes	Raw p over representation	Other species
generation of precursor metabolites and energy	GO:0006091	161	14	0.005	chuck-will's-widow
energy derivation by oxidation of organic compounds	GO:0015980	116	9	0.044	chuck-will's-widow
regulation of production of molecular mediator of immune response	GO:0002700	47	5	0.039	chuck-will's-widow
regulation of B cell mediated immunity	GO:0002712	19	3	0.039	chuck-will's-widow
regulation of immunoglobulin mediated immune response	GO:0002889	19	3	0.039	chuck-will's-widow
cofactor binding	GO:0048037	221	14	0.046	chuck-will's-widow
coenzyme binding	GO:0050662	157	11	0.040	chuck-will's-widow
flavin adenine dinucleotide binding	GO:0050660	61	9	<0.001	chuck-will's-widow
nucleotide-sugar metabolic process	GO:0009225	16	4	0.003	barn owl
peptidyl-prolyl cis-trans isomerase activity	GO:0003755	29	4	0.024	barn owl
hydrogen peroxide metabolic process	GO:0042743	13	3	0.014	ostrich, tinamou
vacuolar membrane	GO:0005774	154	13	0.006	ostrich, tinamou
apical part of cell	GO:0045177	162	11	0.047	ostrich, tinamou
ribonuclease activity	GO:0004540	50	5	0.042	ostrich, tinamou
**sensory perception of light stimulus**	**GO:0050953**	**98**	**9**	**0.017**	**ostrich**
carboxylic acid catabolic process	GO:0046395	95	8	0.037	ostrich
purine ribonucleoside	GO:0009206	42	5	0.026	ostrich

triphosphate biosynthetic process					

Node name	Node ID	No. genes in node	No. significant genes	Raw p over representation	Other species
ribonucleoside triphosphate biosynthetic process	GO:0009201	48	5	0.042	ostrich
RNA phosphodiester bond hydrolysis	GO:0090501	49	5	0.046	ostrich
superoxide anion generation	GO:0042554	14	3	0.017	ostrich
endoribonuclease activity	GO:0004521	25	4	0.014	ostrich
**photoreceptor activity**	**GO:0009881**	**15**	**3**	**0.018**	**ostrich**
nucleus organization	GO:0006997	62	6	0.037	tinamou
transferase activity, transferring phosphorus-containing groups	GO:0016772	767	39	0.044	tinamou
ephrin receptor activity	GO:0005003	13	3	0.012	tinamou

C) *LRT = Likelihood ratio test between kiwi as foreground (model = 2) and the null model (model = 0) as calculated by PAML package [9].

**Gene Name**	***Apteryx mantelli* gene ID**	***Gallus gallus* gene ID**	**ω background**	**ω *Apteryx mantelli***	**LRT**
**eye photoreceptor cell differentiation GO:0001754, sensory perception of light stimulus GO:0050953, visual perception GO:0007601, photoreceptor activity GO:0009881**					
MYO7A	maker-scaffold492-augustus-gene-3.1-mRNA-1	ENSGALG00000000733	0.001	0.076	4.159
TRPM1	augustus_masked-scaffold71-abinit-gene-52.1-mRNA-1	ENSGALG00000003849	0.045	0.393	16.202
RGS9BP	augustus_masked-scaffold150-abinit-gene-103.0-mRNA-1	ENSGALG00000004675	0.022	0.150	6.113
PDE6C	augustus_masked-scaffold37-abinit-gene-1.1-mRNA-1	ENSGALG00000006626	0.027	0.185	6.013
RRH	maker-scaffold39-augustus-gene-121.1-mRNA-1	ENSGALG00000012181	0.126	0.922	8.455
GABRR2	maker-scaffold3-augustus-gene-55.4-mRNA-1	ENSGALG00000015776	0.026	0.241	7.893
cOpn5L2	augustus_masked-scaffold1066-abinit-gene-0.4-mRNA-1	ENSGALG00000016699	0.067	0.409	10.793
CLN5	augustus_masked-scaffold38-abinit-gene-112.0-mRNA-1	ENSGALG00000016917	0.108	0.373	4.199
No ID	augustus_masked-scaffold50-abinit-gene-143.1-mRNA-1	ENSGALG00000016802	0.110	0.666	9.416
**feeding behavior GO:0007631**					
CCK	maker-scaffold354-augustus-gene-73.4-mRNA-1	ENSGALG00000011940	0.133	0.724	5.977
DRD2	augustus_masked-scaffold835-abinit-gene-0.0-mRNA-1	ENSGALG00000007794	0.046	0.407	6.569
STRA6	augustus_masked-scaffold1289-abinit-gene-1.5-mRNA-1	ENSGALG00000001449	0.089	1.651	11.377
HTR2C	augustus_masked-scaffold4345-abinit-gene-0.0-mRNA-1	ENSGALG00000005853	0.067	0.703	9.006
**energy reserve metabolic process GO:0006112**					
STK40	maker-scaffold34-augustus-gene-7.6-mRNA-1	ENSGALG00000002130	0.006	0.223	25.001
PHKB	maker-scaffold150-augustus-gene-70.2-mRNA-1	ENSGALG00000004004	0.074	0.427	10.626
AGL	augustus_masked-scaffold121-abinit-gene-92.0-mRNA-1	ENSGALG00000005407	0.100	0.209	12.094
GYS2	augustus_masked-scaffold27-abinit-gene-541.0-mRNA-1	ENSGALG00000013265	0.030	0.448	4.813
GBE1	maker-scaffold33-augustus-gene-186.4-mRNA-1	ENSGALG00000015506	0.086	0.832	6.413
**Mitochondrion GO:0005739**					
HEATR1	maker-scaffold19-augustus-gene-3.1-mRNA-1	ENSGALG00000000258	0.099	0.489	4.423
C20H20ORF24	augustus_masked-scaffold123-abinit-gene-7.1-mRNA-1	ENSGALG00000001054	0.027	0.275	3.972
ACOX1	maker-scaffold1547-augustus-gene-2.3-mRNA-1	ENSGALG00000002159	0.179	0.542	9.082

**Gene Name**	***Apteryx mantelli* gene ID**	***Gallus gallus* gene ID**	**ω background**	**ω *Apteryx mantelli***	**LRT**
GOT2	maker-scaffold548-augustus-gene-13.2-mRNA-1	ENSGALG00000002321	0.053	0.195	5.830
POLDIP2	maker-scaffold1645-augustus-gene-3.14-mRNA-1	ENSGALG00000003211	0.004	0.210	27.921
LACTB	maker-scaffold71-augustus-gene-30.1-mRNA-1	ENSGALG00000003505	0.129	0.333	4.896
HK1	augustus_masked-scaffold312-abinit-gene-0.0-mRNA-1	ENSGALG00000004222	0.012	0.039	5.689
AIFM2	augustus_masked-scaffold1638-abinit-gene-1.1-mRNA-1	ENSGALG00000004777	0.174	2.824	5.912
PEMT	maker-scaffold2428-augustus-gene-3.1-mRNA-1	ENSGALG00000004875	0.094	0.720	7.871
CHDH	maker-scaffold105-augustus-gene-2.2-mRNA-1	ENSGALG00000005363	0.095	0.429	5.861
No ID	maker-scaffold1167-augustus-gene-3.1-mRNA-1	ENSGALG00000005494	0.026	0.315	6.166
PDE12	augustus_masked-scaffold815-abinit-gene-3.0-mRNA-1	ENSGALG00000005620	0.117	0.212	4.041
BCL2A1	augustus_masked-scaffold118-abinit-gene-5.0-mRNA-1	ENSGALG00000006511	0.160	0.564	8.542
HSPA4	maker-scaffold241-augustus-gene-24.7-mRNA-1	ENSGALG00000007273	0.060	0.306	18.624
MPP7	maker-scaffold308-augustus-gene-59.3-mRNA-1	ENSGALG00000007408	0.033	1.015	31.363
ICT1	maker-scaffold1928-augustus-gene-7.13-mRNA-1	ENSGALG00000007822	0.107	1.010	11.852
TRAF6	augustus_masked-scaffold87-abinit-gene-135.1-mRNA-1	ENSGALG00000007932	0.073	0.604	13.019
ATG13	augustus_masked-scaffold87-abinit-gene-79.3-mRNA-1	ENSGALG00000008347	0.044	0.196	4.268
NDUFA5	maker-scaffold677-augustus-gene-25.6-mRNA-1	ENSGALG00000008821	0.083	157.881	3.947
No ID	augustus_masked-scaffold121-abinit-gene-155.2-mRNA-1	ENSGALG00000008834	0.270	1.259	3.994
ADCK3	maker-scaffold147-augustus-gene-13.3-mRNA-1	ENSGALG00000009082	0.056	0.188	7.158
CRLS1	maker-scaffold10-augustus-gene-41.2-mRNA-1	ENSGALG00000009214	0.007	0.284	13.245
IARS2	augustus_masked-scaffold32-abinit-gene-90.0-mRNA-1	ENSGALG00000009566	0.131	0.302	5.423
FLVCR1	maker-scaffold32-augustus-gene-52.2-mRNA-1	ENSGALG00000009807	0.065	1.369	7.988
MRPS18A	augustus_masked-scaffold19-abinit-gene-96.2-mRNA-1	ENSGALG00000010296	0.137	1.173	9.235
ALKBH1	augustus_masked-scaffold15-abinit-gene-146.0-mRNA-1	ENSGALG00000010485	0.183	0.501	6.287
ADCK1	maker-scaffold15-augustus-gene-147.3-mRNA-1	ENSGALG00000010504	0.050	0.340	4.968
SNX13	augustus_masked-scaffold151-abinit-gene-120.0-mRNA-1	ENSGALG00000010840	0.037	0.200	7.427
LARS2	augustus_masked-scaffold354-abinit-gene-61.0-mRNA-1	ENSGALG00000011877	0.166	0.400	4.529
XPNPEP3	augustus_masked-scaffold27-abinit-gene-324.0-mRNA-1	ENSGALG00000012003	0.160	0.536	4.591

**Gene Name**	***Apteryx mantelli* gene ID**	***Gallus gallus* gene ID**	**ω background**	**ω *Apteryx mantelli***	**LRT**
CCDC109B	maker-scaffold39-augustus-gene-119.1-mRNA-1	ENSGALG00000012196	0.140	0.842	10.239
PPP3CA	maker-scaffold39-augustus-gene-84.3-mRNA-1	ENSGALG00000012280	<0.001	0.686	36.131
FASTKD3	maker-scaffold110-augustus-gene-14.6-mRNA-1	ENSGALG00000013054	0.350	1.458	9.075
SAMM50	maker-scaffold27-augustus-gene-567.3-mRNA-1	ENSGALG00000014194	0.033	0.771	32.540
MRPL39	maker-scaffold57-augustus-gene-38.1-mRNA-1	ENSGALG00000015742	0.105	0.888	7.592
SOD1	augustus_masked-scaffold57-abinit-gene-64.1-mRNA-1	ENSGALG00000015844	0.169	1.206	7.717
No ID	augustus_masked-scaffold57-abinit-gene-77.0-mRNA-1	ENSGALG00000016007	0.080	0.403	5.759
AGPAT5	maker-scaffold45-augustus-gene-10.1-mRNA-1	ENSGALG00000016329	0.208	1.384	7.099
Tpo	augustus_masked-scaffold693-abinit-gene-9.0-mRNA-1	ENSGALG00000016370	0.075	6.023	7.469
RSAD2	augustus_masked-scaffold55-abinit-gene-11.1-mRNA-1	ENSGALG00000016400	0.135	0.653	8.632
MRPL48	maker-scaffold289-augustus-gene-0.3-mRNA-1	ENSGALG00000017319	0.090	0.305	3.889
PICK1	augustus_masked-scaffold27-abinit-gene-341.2-mRNA-1	ENSGALG00000019313	0.016	0.146	4.794
UNG	augustus_masked-scaffold1033-abinit-gene-3.3-mRNA-1	ENSGALG00000021285	0.032	0.333	7.037
FEN1	augustus_masked-scaffold1654-abinit-gene-0.2-mRNA-1	ENSGALG00000024076	0.027	0.139	6.155

Supplementary Table S9. Gene Ontology categories enriched for genes, which evolve slower in kiwi; (A) kiwi-specific and (B) shared with any of the other tested *Palaeognathae* (ostrich and tinamou) or nocturnal birds (chuck-will's-widow and barn owl). The enrichment was performed using a hypergeometric test [19] on genes evolving significantly slower in kiwi. The background species considered in CODEML [9] were chicken, turkey, zebra finch, ostrich, tinamou, chuck-will's-widow, and barn owl. (C) Genes belonging to the potentially biological relevant categories for kiwi's specific physiology. None of the categories remained significant after family-wise error rate multiple testing correction.

A)

Node name	Node ID	No. genes in node	No. significant genes	Raw p over representation
cellular amino acid biosynthetic process	GO:0008652	67	4	0.004
negative regulation of catabolic process	GO:0009895	68	4	0.005
regulation of ligase activity	GO:0051340	25	3	0.002
cellular modified amino acid biosynthetic process	GO:0042398	29	3	0.003
negative regulation of cellular catabolic process	GO:0031330	42	3	0.008
protein deacetylation	GO:0006476	49	3	0.012
protein deacylation	GO:0035601	52	3	0.015
sulfur compound biosynthetic process	GO:0044272	55	3	0.017
B cell proliferation	GO:0042100	60	3	0.021
chromosome, telomeric region	GO:0000781	36	3	0.005
**mitochondrial outer membrane**	**GO:0005741**	**56**	**3**	**0.018**
mRNA binding	GO:0003729	80	4	0.008
double-stranded RNA binding	GO:0003725	33	3	0.004
chaperone binding	GO:0051087	37	3	0.006
unfolded protein binding	GO:0051082	53	3	0.016

B)

Node name	Node ID	No. genes in node	No. significant genes	Raw p over representation	Other species
**anion channel activity**	**GO:0005253**	**49**	**3**	**0.013**	**chuck-will's-widow**
cell aging	GO:0007569	58	3	0.020	ostrich
negative regulation of proteasomal protein	GO:1901799	19	3	0.001	tinamou

catabolic process

Node name	Node ID	No. genes in node	No. significant genes	Raw p over representation	Other species
negative regulation of proteolysis	GO:0045861	32	3	0.004	tinamou
telomere maintenance	GO:0000723	33	3	0.004	tinamou
telomere organization	GO:0032200	33	3	0.004	tinamou
negative regulation of protein catabolic process	GO:0042177	41	3	0.008	tinamou

C)

Gene Name	*Apteryx mantelli* gene ID	*Gallus gallus* gene ID	ω background	ω *Apteryx mantelli*	LRT
**anion channel activity GO:0005253**					
VDAC2	maker-scaffold241-augustus-gene-43.2-mRNA-1	ENSGALG00000005002	0.034	0.007	5.753
CLIC4	augustus_masked-scaffold57-abinit-gene-82.0-mRNA-1	ENSGALG00000001262	0.031	0.002	5.057
NMUR2	augustus_masked-scaffold1749-abinit-gene-0.0-mRNA-1	ENSGALG00000004101	0.049	0.004	5.070
**mitochondrial outer membrane GO:0005741**					
VDAC2	maker-scaffold241-augustus-gene-43.2-mRNA-1	ENSGALG00000005002	0.034	0.007	5.753
TMEM173	augustus_masked-scaffold517-abinit-gene-13.1-mRNA-1	ENSGALG00000000852	0.275	<0.001	4.002
SPATA18	augustus_masked-scaffold31-abinit-gene-56.0-mRNA-1	ENSGALG00000013964	0.363	<0.001	5.251

Supplementary Table S10. Selection scan on opsins in kiwi, chuck-will's-widow, barn owl ostrich, tinamou, chicken, turkey, and zebra finch.

LRT1 = likelihood ratio testing with one degree of freedom, between the null model (model = 0) and a model where the appointed branch differs from the other birds (model = 2), implemented in CODEML from the PAML package.

LRT2 = likelihood ratio testing with one degree of freedom, between the model 2 with estimated ω and the model 2 with ω fixed to 1 (neutral evolution).

* ω = 999.000 shows the absence of synonymous differences (the ratio is estimated to be infinity, while LRT is not affected) [9].

RHO	**ω background**	**ω foreground**	**LRT1**	**LRT2**
kiwi	0.044	0.149	6.128	16.705
chuck-will's-widow	0.043	0.143	6.631	22.917
barn owl	0.055	0.146	0.421	1.215
ostrich	0.052	0.125	1.444	7.610
tinamou	0.067	<0.001	10.336	62.053
OPN1LW	**ω background**	**ω foreground**	**LRT1**	**LRT2**
kiwi	0.156	0.597	1.503	0.078
chuck-will's-widow	0.156	999.000	1.762	0.230
barn owl	0.122	0.529	6.898	1.290
ostrich	0.180	0.105	0.503	15.074
tinamou	0.177	0.007	2.537	16.073
OPN1MW	**ω background**	**ω foreground**	**LRT1**	**LRT2**
kiwi	0.021	0.268	44.951	10.505
chuck-will's-widow	0.033	0.045	0.669	105.178
barn owl	0.035	<0.001	-0.003	0.001
ostrich	0.037	0.008	3.396	41.691
tinamou	0.038	0.019	1.878	93.631
OPN3	**ω background**	**ω foreground**	**LRT1**	**LRT2**
kiwi	0.110	0.542	3.211	0.469
chuck-will's-widow	0.110	0.232	1.652	8.469
barn owl	0.119	999.000	3.030	0.716
ostrich	0.123	0.122	-0.001	12.624
tinamou	0.106	0.237	2.063	8.724
OPN4-1	**ω background**	**ω foreground**	**LRT1**	**LRT2**
kiwi	0.142	0.231	2.733	1.548
chuck-will's-widow	0.141	0.376	5.364	5.107
barn owl	0.148	0.206	0.710	18.255
ostrich	0.142	999.000	3.603	0.044
tinamou	0.162	0.070	0.900	0.900

OPN4-2	**ω background**	**ω foreground**	**LRT1**	**LRT2**
kiwi	0.186	2.574	8.194	0.884
chuck-will’s-widow	0.221	0.094	2.507	24.981
barn owl	0.190	0.487	2.509	1.519
ostrich	0.191	0.282	0.885	11.799
tinamou	0.221	0.156	0.968	38.517

OPN5	**ω background**	**ω foreground**	**LRT1**	**LRT2**
kiwi	0.071	<0.001	1.733	12.097
chuck-will’s-widow	0.066	0.101	0.355	16.568
barn owl	0.069	0.069	-0.001	15.557
ostrich	0.068	0.083	0.063	13.531
tinamou	0.077	<0.001	5.550	39.148

opsin-VA-like	**ω background**	**ω foreground**	**LRT1**	**LRT2**
kiwi	0.317	0.262	0.035	2.252
chuck-will's-widow	0.324	<0.001	0.884	2.018
barn owl	0.254	1.266	1.560	0.036
ostrich	0.305	0.753	<0.001	<0.001
tinamou	0.305	0.331	<0.001	0.082

Supplementary Table S11. Loss of ancestral footprint clusters in *HOX* genes. Differential loss of conserved, ancestral, non-coding DNA elements was quantitatively assessed in kiwi versus chicken and duck. No obvious change on the kiwi branch was found.

Loss measures in number of footprints					
	**present in:**	**lost in:**			
	2 outgroups	All	*Gallus gallus*	*Anas platyrhynchos*	*Apteryx mantelli*
HOXA	694	211	81	85	60
HOXB	698	181	121	100	85
HOXC	442	38	126	30	56
HOXD	589	144	51	44	43
Loss measures in number of nucleotides in footprints					
	**present in:**	**lost in:**			
	2 outgroups	all	*Gallus gallus*	*Anas platyrhynchos*	*Apteryx mantelli*
HOXA	19,332	8,848	1,241	1,944	1,263
HOXB	18,875	7,151	2,978	2,322	2,266
HOXC	10,606	1,663	3,411	606	1,141
HOXD	15655	5,689	954	917	1,337

Supplementary Table S12. Genes involved in limb development [4, 20-27] that were manually surveyed in the *AptMant0* genome. ω (Ka/Ks ratio) was calculated using multiple alignments including kiwi and at least three other bird species from chicken, turkey, zebra finch, flycatcher, falcon, and rock dove with the program PAML [9]. ω0 is the average Ka/Ks ratio in all branches (model = 0), ω1 is the average ratio in non-kiwi background branches (model = 2), and ω2 is the ratio in kiwi (model = 2, kiwi appointed as foreground branch). LRT = Likelihood ratio test between kiwi as foreground and the null model (lines in bold denote a significant Chi square test, p-value < 0.05).

No obvious differences were noticed after comparative analysis with chicken, turkey, and zebra finch orthologous genes.

External Gene ID	*AptMant0* annotation ID	Involvement in limb development	ω0	ω1	ω2	LRT
fgf4	maker-scaffold87-augustus-gene-159.3-mRNA-1	Fgf signaling / can lead to ectopic limb formation	0.509	0.475	0.566	0.21
fgf7	augustus_masked-scaffold71-abinit-gene-124.0-mRNA-1	Fgf signaling / can lead to ectopic limb formation	0.193	0.167	0.239	0.60
fgf8	augustus_masked-scaffold755-abinit-gene-1.0-mRNA-1	Signals from intermediate mesoderm; Fgf signaling / can lead to ectopic limb formation	0.015	0.018	0.0001	1.76
fgf17	maker-scaffold605-augustus-gene-3.9-mRNA-1	Fgf signaling	0.043	0.039	0.076	0.74
fgf18	augustus_masked-scaffold266-abinit-gene-7.0-mRNA-1	Fgf signaling	0.09	0.097	0.0001	2.01
fgf20	maker-scaffold31-augustus-gene-15.2-mRNA-1	Fgf signaling	0.007	0.0001	0.029	2.66
fgfr1	**maker-scaffold1100-augustus-gene-1.9-mRNA-1**	**Fgf signaling / can lead to ectopic limb formation**	**0.029**	**0.047**	**0.004**	**17.21**
gli2	augustus_masked-scaffold1176-abinit-gene-0.0-mRNA-1	Limb positioning along anterior-posterior axis	0.079	0.078	0.083	0.09
gli3	maker-scaffold602-augustus-gene-40.4-mRNA-1	Limb positioning along anterior-posterior axis	0.205	0.182	0.278	2.95
sall4	augustus_masked-scaffold388-abinit-gene-25.0-mRNA-1	Patterning and morphogenesis of forelimb	0.124	0.123	0.123	< 0.001
shh	maker-scaffold42-augustus-gene-36.2-mRNA-1	Limb positioning along anterior-posterior axis	0.0001	0.0001	0.0001	0
tbx4	augustus_masked-scaffold1311-abinit-gene-9.0-mRNA-1	Signals from intermediate mesoderm (hindlimb)	0.010	0.011	0.006	0.78
tbx5	maker-scaffold1382-augustus-gene-2.3-mRNA-1	Signals from intermediate mesoderm (forelimb)	0.014	0.015	0.009	0.27

External Gene ID	*AptMant0* annotation ID	Involvement in limb development	ω0	ω1	ω2	LRT
wnt1	augustus_masked-scaffold801-abinit-gene-0.3-mRNA-1	Limb outgrowth and formation / axes formation	0.014	0.016	0.014	0.09
wnt11	**maker-scaffold661-augustus-gene-0.3-mRNA-1**	**Limb outgrowth and formation / skeletal differentiation**	**0.034**	**0.016**	**0.098**	**6.08**
wnt2b	augustus_masked-scaffold185-abinit-gene-11.1-mRNA-1	Signals from intermediate mesoderm (forelimb) / dorso-ventral patterning	0.032	0.036	0.017	1.38
wnt4	maker-scaffold898-augustus-gene-1.3-mRNA-1	Limb joint formation	0.001	0.0001	0.005	2.25
wnt5b	maker-scaffold27-augustus-gene-468.2-mRNA-1	Limb outgrowth and formation / Skeletal differentiation	0.0001	0.0001	0.0001	0
wnt6	augustus_masked-scaffold95-abinit-gene-79.1-mRNA-1	Negative regulator of limb chondrogenesis in chicken	0.004	0.003	0.007	1.07
wnt8b	**augustus_masked-scaffold842-abinit-gene-8.2-mRNA-1**	**Limb outgrowth and formation**	**0.043**	**0.059**	**0.007**	**4.50**
wnt9b	**augustus_masked-scaffold721-abinit-gene-1.0-mRNA-1**	**Limb joint formation**	**0.038**	**0.049**	**0.009**	**6.85**

Supplementary Table S13. Ultra-conserved non-coding elements (UCNEs) with more than the expected 5% sequence change in *Apteryx mantelli*. UCNEs were downloaded from UCNEbase (http://ccg.vital-it.ch/UCNEbase) [12], orthology in each genome was established using BLAST [28], the orthologous sequence from each genome was aligned [29] to the chicken sequence annotated in the database and number of nucleotide changes were counted.

A.m. = Apteryx mantelli, S.c. = Struthio camelus, T.gt. = Tinamus guttatus, T.a. = Tyto alba, A.cs. = Antrostomus carolinensis, F.a = Ficedula albicollis, T.g. = Taeniopygia guttata, A.p. = Anas platyrhynchos, M.g. = Meleagris gallopavo.

**UCNE ID**	***A.m.***	***S.c.***	***T.gt.***	***T.a.***	***A.cs.***	***F.a.***	***T.g.***	***A.p.***	***M.g.***	**Length UCNE**	**%Change in *A.m.***	**%Mean change in *S.c.* and *T.gt.***	**%Mean change in other birds**	**Position in *Gallus gallus* genome**	**Position in *Apteryx mantelli* genome**
BCL11A Andrew	12	9	19	11	11	11	10	11	3	229	5.24	6.11	4.15	chr3:769195-769423	scaffold570:596014-596242
HOXA Larisa	19	14	21	6	8	7	7	2	1	357	5.32	4.90	1.45	chr2:32403104-32403460	scaffold151:16384352-16384708
MECOM Joshua	29	7	10	6	5	8	9	9	1	395	7.34	2.15	1.60	chr9:3922810-3923204	scaffold221:2205502-2205110
NFIA Franz	20	15	25	6	8	7	7	3	2	218	9.17	9.17	2.52	chr8:27842134-27842351	scaffold329:42894-43103
NKX6-1 Leo	17	17	24	8	4	5	5	5	1	266	6.39	7.71	1.75	chr4:48216654-48216919	scaffold564:827247-827510
NR4A2 Aphrodite	23	11	0	5	4	8	13	2	0	358	6.42	1.54	1.49	chr7:37480363-37480720	scaffold22:1586820-1586462
PAX5 Elvira	15	8	8	2	2	8	8	2	3	219	6.85	3.65	1.90	chrZ:325085-325303	scaffold1246:159-374
SOX2 Mustafa	17	1	13	0	0	0	0	0	0	236	7.20	2.97	0.00	chr9:7512153-7512388	scaffold14:1546151-1545917
SP8 Scarlett	11	6	6	3	3	12	7	4	1	215	5.12	2.79	2.33	chr2:30536926-30537140	scaffold151:13909784-13909998

TBX2 Santiago	14	11	14	11	15	11	12	14	5	222	6.31	5.63	5.11	chr19:2493855-2494076	scaffold1311:713904-713686

**UCNE ID**	***A.m.***	***S.c.***	***T.gt.***	***T.a.***	***A.cs.***	***F.a.***	***T.g.***	***A.p.***	***M.g.***	**Length UCNE**	**%Change in *A.m.***	**%Mean change in *S.c.* and *T.gt.***	**%Mean change in other birds**	**Position in *Gallus gallus* genome**	**Position in *Apteryx mantelli* genome**
TFAP2A Julia	27	8	9	7	7	11	9	11	3	402	6.72	2.11	1.99	chr2:90071047-90071448	scaffold100:13570062-13569662
TLE4 Robert	15	11	10	9	14	8	10	5	4	242	6.20	4.34	3.44	chrZ:38726392-38726633	scaffold3784:7926-7685
TRPS1 Eros	14	8	7	8	4	6	7	0	0	221	6.33	3.39	1.89	chr2:140299043-140299263	scaffold7:7589779-7589988
ZC3H3 Figaro	33	4	8	4	2	5	6	1	1	217	15.21	2.76	1.46	chr2:154388655-154388871	scaffold3568:110159-110372
ZNF503 Amos	16	21	20	12	6	10	8	7	4	302	5.30	6.79	2.59	chr6:21267923-21268224	scaffold93:2528315-2528614
chr1 Eden	23	8	4	6	6	11	10	7	3	252	9.13	2.38	2.84	chr8:29352506-29352757	scaffold1709:99854-99616
chr4 Adam	17	13	12	12	13	18	17	6	5	319	5.33	3.92	3.71	chr4:81608502-81608820	scaffold92:10278731-10279049
chr9 Cassandra	18	15	34	14	12	14	22	7	6	268	6.72	9.14	4.66	chrZ:66204066-66204333	scaffold1406:397946-397679
chr9 Delphina	12	8	6	8	5	2	8	8	4	206	5.83	3.40	2.83	chrUn_random:31824020-31824225	scaffold958:84653-84854

Supplementary Table S14. Primers used to amplify the coding sequence of *fibin* in kiwi.

Primer name	Chicken coding sequence coordinates	Species sequence	Sequence (5' -> 3')
fibin forward 1	Fibin cDNA:71-89	Ostrich	TGCAGCCGGAGATGTCCAA
fibin forward 2	Fibin cDNA:95-113	Chicken	CCCTGCACCACTACTTCGT
fibin reverse 1	Fibin cDNA:275-294	Ostrich	CTAGCCGATGTCCTCCAGCA
fibin reverse 2	Fibin cDNA:291-311	Chicken	AGGTCGTAGGAGATGCTCTTG
fibin reverse 3	Fibin cDNA:372-391	Chicken	AGCGGTCCGAGCTGGAGTAG

Supplementary Table S15. Assembly metrics for available *Palaeognathae* genomes. *Tinamus guttatus* (release 2014-04-30) and *Struthio camelus* (release 2014-08-13) were downloaded from GigaDB [17].

	Tinamus guttatus	***Struthio camelus***	***Apteryx mantelli***
Number of scaffolds	175,907	32,012	326,827
Total size of scaffolds (bp)	1,059,687,971	1,257,873,544	1,595,278,775
Longest scaffold (bp)	1,986,275	50,413,810	63,182,071
Number of scaffolds > 1K nt	23,642	1,890	24,710
Number of scaffolds > 10K nt	7,466	725	6,641
Number of scaffolds > 100K nt	2,883	309	1,040
Number of scaffolds > 1M nt	39	110	221
Number of scaffolds > 10M nt	0	36	32
N50 scaffold length (bp)	242,236	17,714,005	3,956,354
Scaffold %A	28.65	27.84	25
Scaffold %C	20.3	19.41	18
Scaffold %G	20.28	19.39	18
Scaffold %T	28.62	27.8	25
Scaffold %N	2.16	5.56	13
Number of contigs	227,964	94,215	508,831
Total size of contigs	1,037,038,325	1,188,122,451	1,382,272,215
Longest contig	317,681	436,179	166,809
Number of contigs > 1K nt	64,872	52,847	146,153
Number of contigs > 10K nt	25,927	29,660	40,984
Number of contigs > 100K nt	785	1241	69
Number of contigs > 1M nt	0	0	0
N50 contig length	35,048	43,467	16,480

Supplementary Table S16. Scaffolds from the kiwi and ostrich assemblies, which span same two chromosomes in the chicken genome.

Target genome	Galgal4 position	Target genome position	Alignment score	Galgal4 position	Target genome position	Alignment score
ostrich	chr2:124,565,139-124,565,947	scaffold997:18,814-19,709	9,205	chrZ:67,076,675-67,076,824	scaffold997:16,127-16,276	4,910
kiwi	chr2:77,540,308-77,540,590	scaffold13813:10,432-10,712	12,795	chrZ:22,315,677-22,318,094	scaffold13813:10-2,461	8,916
kiwi	chr2:43,004,513-43,004,744	scaffold22667:1,050-1,277	8,531	chrZ:16,808,869-16,810,200	scaffold22667: 9,587-11,367	31,871
kiwi	chr2:70,348,445-70,348,692	scaffold2391:205-411	3,940	chrZ:63,580,918-63,581,663	scaffold2391:10,711-11,890	22,186
kiwi	chr2:79,500,563-79,500,792	scaffold44678: 2,679-2,886	9,124	chrZ:56,913,078 -56,914,146	scaffold44678:1,578-2,598	20,265
ostrich	chr3:104,577,316-104,577,823	scaffold939:23,377-23,931	17,397	chr5:449,083-449,295	scaffold939:506-721	7,077
kiwi	chr3:4,114,510-4,114,768	scaffold4691:26,756-27,006	5,702	chr5:492,662-492,914	scaffold4691:12,460-12,734	9,185

Supplementary Table S17. Mitochondrial genomes used in the phylogenetic analysis (**Error! Reference source not found.**).

GeneBank accession ID	Species scientific name	Species common name
13116556	*Dinornis giganteus*	North Island Giant Moa
13116573	*Emeus crassus*	Eastern Moa
14141877	*Dromaius novaehollandiae*	Emu
14141905	*Struthio camelus*	Ostrich
14141919	*Anomalopteryx didiformis*	Bush Moa
30387848	*Pterocnemia pennata*	Darwin's Rhea
46255316	*Apteryx haastii*	Great Spotted Kiwi
46326805	*Tinamus major*	Great Tinamou
46358645	*Eudromia elegans*	Tinamou
48864618	*Casuarius casuarius*	Southern Cassowary
49616788	*Smaug warreni*	Warren's Girdled Lizard
89274114	*Taeniopygia guttata*	Zebra Finch
189342971	*Anas platyrhynchos*	Duck
323690831	*Meleagris gallopavo*	Turkey
515021884	*Ficedula albicollis*	Flycatcher
584458524	*Gallus gallus*	Chicken
641804046	*Mullerornis agilis*	Elephant Bird

Supplementary Note

Sampling, DNA library preparation and sequencing

Since birds have female heterogamety, we sampled female kiwi to assure that the W chromosome is included in the assembly. The sequenced individuals originate from the far North (kiwi code 73) and central part – Lake Waikaremoana (kiwi code AT5 and kiwi code 16-12) of North Island (Supplementary Fig. S10). They were sampled in 1986 (kiwi code 73) and 1997 (kiwi code AT5 and 16-12) in 'operation nest egg' carried out by Rainbow and Fairy Springs, Rotorua. The genome was assembled with iwi approval.

Genomic DNA was extracted from *Apteryx mantelli* embryos using standard procedures. To assemble the kiwi genome, we generated both small-insert-size and long-insert-size libraries using the genomic DNA from two of the individuals. The DNA from the first individual (code 73) yielded small-insert-size libraries (240, 420, and 800 bp) and long-insert-size mate-paired-end libraries (2, 3, and 4 kb). The second individual (code 16-12) was used for building solely longer-insert mate-paired-end libraries (7, 9, 11, and 13 kb). In addition, we generated a small-insert-size library for a third individual (code AT5) that is used in genomic analyses regarding population diversity and validation of mutations, but it was not included in the assembly (Supplementary Table S1; Supplementary Fig. S10).

DNA small-insert-size libraries were constructed using the standard TruSeq Illumina kit, following the protocol provided by the manufacturer. Briefly, a total of 7 µg genomic DNA were sheared by sonication with Bioruptor® Standard using each time 1 µg DNA in 100 µl total volume and adjusting the number of cycles according to the desired fragment size. The fragment ends were enzymatically polished using a polymerase with 3' -> 5' activity to obtain blunt ends and ligate the Illumina adaptors to the products. Fragments were purified with AMPure SPRI bead (Agencourt, Beverly, MA) to yield the desired size. Additionally, a 2% agarose gel extraction was performed after the final

amplification step, requiring the insert size of a library to fall in a narrow range, with a variation of less than 10% of the size of the peak, to further simplify the assembly process. The insert sizes of all libraries were assessed using DNA 1000 chips on the 2100 Bioanalyzer (Agilent Technologies, Santa Clara, CA).

Long-insert mate-paired-end libraries (2, 3, and 4 kb) were prepared using the mate pair library Illumina kit. Briefly, 15-20 µg of DNA was fragmented by sonication, and biotin-labeled dNTPs were used for polishing the ends. A 1.5% low molecular weight agarose gel was run and bands at 2 kb, 3 kb, and 4 kb were selected allowing for 10% variation in size. Selected DNA fragments were circularized overnight. The remaining linear fragments were digested using Exonuclease I so that the circularized DNA could be sheared by sonication on Bioruptor® Standard. The merged ends were captured on streptavidin magnetic beads Dynabeads M-270 (Invitrogen), given the biotin-streptavidin interaction. The protocol was continued following the same steps as previously described for the short-insert-size libraries.

Longer-insert mate-paired-end libraries (7, 9, 11, and 13 kb) were generated with the Nextera Mate Pair Sample Preparation kit from Illumina using DNA extracted from a different female individual (code 16-12). The protocol required an input of 4 µg DNA that was fragmented with a Tagment enzyme, which attached a biotinylated junction adapter to both ends of the tagmented molecule. A 0.6% megabase agarose gel was run and bands at 7 kb, 9 kb, 11 kb, and 13 kb were excised allowing for 10% variation in size. Size selection was further confirmed by running a DNA 12000 chip on the 2100 Bioanalyzer (Agilent Technologies, Santa Clara, CA). Circularization was run overnight and the following steps were followed as described above. The average range of the final libraries was 600 bp, which prevented forward and reverse reads (with a sequence length of 96 bp each) from overlapping in sequence.

Sequencing was performed on the Illumina HiScanSQ System (Core Unit Sequencing, University of Leipzig) for short-insert-size and the long-insert mate-paired libraries.

Clusters were generated after quantification of the libraries using the Library Quantification Kit – Illumina/Universal (KAPABiosystems) according to the instructions of the manufacturer. Library DNA at a concentration of 10 pM was clustered using an Illumina cBot according to the PE_Amp_Lin_Block_Hybv8.0 protocol of the manufacturer. Paired-end sequencing with reads of 101 bp was performed with an Illumina HiScanSQ sequencer using version 3 chemistry and the version 3 flowcell according to the manufacturer's instructions. Median cluster density was usually about 600,000 clusters per mm^2 or 80–100 million raw clusters per lane.
Longer-insert mate-paired-end libraries were sequenced on the Illumina HiSeq Platform at the Max Planck Institute for Evolutionary Anthropology Leipzig. Briefly, the libraries were quantified using Qubit® 2.0 Fluorometer (Invitrogen) with a High Sensitivity Kit and 6 pM of the pooled libraries were used for cluster generation. Clusters were formed using Illumina cBot following PE_Amp_Lin_Block_TubeStripHyb, v8 instructions of the manufacturer. Paired-end 96 bp-sequencing reads were generated on an Illumina HiSeq 2500 platform using TruSeq SBS kit v3 – HS. Quality parameters and cluster density (800,000–1,000,000 clusters/mm^2) were checked via the Illumina Sequencing Analysis Viewer.

Filtering and read correction

Sequences were adapter-trimmed and filtered for adapter dimers and chimeras using scripts included in leeHom [30]. Reads with more than 5 bases with quality scores below 15 (PHRED-scale) were discarded. If the forward and reverse reads overlapped for more than 11 bp, the two reads were merged. For the overlapping part, a consensus sequence was determined by calling the base with the highest quality score. Additionally, resulting merged reads that were shorter than 60 bp were discarded.

Sequencing errors pose a challenge for the *de Bruijn* graph short-read assembly algorithm. However, methods exist that can correct some sequencing errors based on

the frequency of *k-mers* in reads. After concatenating the data from all short insert libraries, the frequencies of all 19-mers in the dataset were counted using Jellyfish [31]. This distribution (Supplementary Fig. S1) was provided to Quake [1] which uses the information to correct reads from each of the short-insert-size libraries separately (Supplementary Table S1). In sum, 36.5% of the total short-insert-size library sequences were removed which left 52.53 Gb of usable high-quality sequences. The corrected reads were used for *de novo* assembly.

Genome assembly

We assembled the kiwi genome with SOAPdenovo [32] v2.04 using a *k-mer* size of K = 23 and default parameters. Only the corrected short-insert-size libraries were used for contig building. As recommended by SOAPdenovo [32], long-insert-size libraries were not included in the initial contig generation as these may introduce more misassembly due to the library construction approach in which circularization can lead to chimeras of a small fraction of reads that come from two different molecules that were misjoined during circularization.

In the contiging step 40 billion *k-mers* were processed and 1.33 billion linear nodes were constructed. During the graph simplification step 14.36 million tips were removed because they contained *k-mers* that were not well connected. An unambiguous graph path was constructed from the retained *k-mers* and contigs were reported, considering a size of 100 bp as the shortest length. Contigs summed up to 1.227 Gb, with average lengths of 731 bp, N50 of 1,550 bp, and N90 of 281 bp.

Scaffolds were built by joining contigs using paired-end reads from libraries of all sizes, including small-inserts. The scaffolds were constructed starting with small-insert-size libraries, and continuing with long-distance mate-paired-end libraries, ending with the 13-kb library. At least 3 consistent read pairs from short-insert-size libraries or 5 read pairs for the mate-pair-end libraries were required to support the joining of two contigs.

An overview of the assembly statistics at different stages is given in Supplementary Table S2. Using only 240, 420, and 800 bp data for scaffolding resulted in N50 of 31,909 bp and an N90 of 2,394 bp. The addition of the 2-kb insert-size library resulted in N50 and N90 of 32,914 bp and 2,581 bp, respectively. Adding the 3-kb library data improved the N50 and N90 to 66,802 bp and 4,737 bp, respectively. Finally, adding the 4 to 13-kb libraries led to an N50 of 5,026,352 bp and N90 of 35,043 bp. This assembly spans 1.816 Gb with 33.15% unknown (missing) bases.

To close the gaps in the scaffolds we used GapCloser from the SOAP package [32]. Briefly, the paired-end information from the corrected short-insert-size libraries was used as long as one read mapped to the scaffolds, while its pair was located in a gap region. These reads were locally assembled by constructing a *de Bruijn* graph similar to the contiging process. This resulted in an assembly with 13.37% unresolved bases. The contig N50 improved from 1,550 bp to 16.48 kb and also the average length of breaks in scaffolds dropped from 1,841 to 1,170 bp after filling the gaps. The length of the assembly decreased slightly to 1.595 Gb with an N50 of 3.96 Mb and 221 scaffolds longer than 1 Mb that sum up to 69% of the total assembly (Supplementary Table S3). Subsequent analyses were done on the gap-closed assembly (*AptMant0*).

Whole genome alignments

To facilitate comparative analyses with other birds, the scaffolds from the kiwi genome assembly (*AptMant0*) were aligned to the chicken genome (*Gallus gallus Galgal4)* and zebra finch (*Taeniopygia guttata taeGut3.2.4*) [18] using Lastz [33] with default parameters. The UCSC Genome Browser whole genome alignment pipeline [34] was used to process the alignments. Thus, *axtChain* (with parameters: minScore = 1000, linearGap = loose, *chainAntiRepeat, chainMergeSort, chainPreNet, chainNet*) and *netSyntenic* were used. Scaffolds were chained and netted to the chicken chromosomes and liftover files were further generated using *netChainSubset* and *ChainStitchId.* Files

were converted between maf and axt formats using *MafToAxt, netSplit, netToAxt, axtSort* and *axtToMaf*. All programs were compiled from jksrc v 130 downloaded 2009.07.07. A total of 799,092,865 bp were chained to the chicken chromosomes, of which 193,978,601 bp (24.27%) differed from the chicken reference (Supplementary Table S5). The percentage difference to the zebra finch chromosomes was 24.64%.

To detect whether any chromosomal rearrangements happened in the ratites, we also aligned the ostrich genome on a per chromosome basis to the chicken genome (*Gallus gallus Galgal4)* using Lastz [33] with default parameters. Given the high fragmentation of the tinamou assembly (Supplementary Table S15), this genome could not be used in this analysis. The best alignment to each locus for each chromosome was retained for both kiwi and ostrich using *axtBest* from the UCSC Genome Browser [34]. A minimal alignment score of 1,000 was applied to reduce false positives. A list of scaffolds where one segment maps to one chromosome and the other maps to a different one, regardless of stand orientation, was produced. Since true rearrangements are hard to distinguish from misassemblies, we focused on scaffolds that spanned the same two different chicken chromosomes in both kiwi and ostrich.

A previous study has reported a rearrangement between the reptiles (Red-tailed Boa and Green Anole Lizard) chromosome 2 and ostrich pseudo chromosome Z [35]. Although a rearrangement in kiwi and ostrich is suggested between chromosomes 2 and Z from chicken, the coordinates of the hits do not overlap for the ostrich and kiwi genomes (Supplementary Table S16). To confidently perform such an analysis, further improvement of both assemblies is necessary.

Genome coverage and estimation of genome size

Sequencing errors can interfere with the coverage estimation from the *k-mer* distribution. To estimate the genome sequencing depth more accurately, all corrected short-insert-size libraries were realigned to the assembled genome (*AptMant0*). All

reads that mapped to scaffolds were considered. An average coverage of 31.74-fold was obtained. The distribution of the coverage density was plotted for a randomly chosen subset of 1% of the sites pertaining to the 1,000 longest scaffolds for a total of 101.7 Mb sites. The average coverage computed on that subset was 35.85-fold (Supplementary Fig. S14). GC content was computed across the 1,000 longest scaffolds and coverage was plotted against the GC content. A mean of 41% GC bases was calculated. The genome-wide coverage is observed in regions with GC content between 25% and 62% (Supplementary Fig. S13). Coverage is lower outside of this range of GC content.

Information about the genome size of kiwi is unavailable. To check how much of the genome is covered by the *AptMant0*, we estimated the genome size starting from the *k*-mer distribution (Supplementary Fig. S1). *K-mer* counts show a mixture of two distributions: the coverage of true *k-mers* and the coverage of erroneous *k-mers*. Erroneous *k-mers* are considered the ones that have low coverage, but high frequency and follow a Poisson distribution, while true *k-mers* have high coverage and follow a Gaussian distribution. The cutoff that separates true and erroneous *k-mers* was estimated at a coverage of 5-fold, after which the *k-mers* frequency distribution followed a bell-shaped curve. The peak of the true *k-mer*-distribution is at 31-fold. *K-mers* cannot span over the edge of reads, hence their abundance is underestimated. To circumvent this, the real sequencing depth (D) was calculated starting from the observed coverage ($C_k = 31$) of a *k-mer* (of size K = 19 nt) in reads of length L (L = 101 nt); $D = C_k * L/(L-K+1)$ [1] and is 37-fold for the true *k-mers* distribution. The percentage of *-mers* that have a coverage higher than 5, and are considered truly part of the genome is 68.53%. This is an underestimate, as part of the *k-mers* with lower coverage may still be true *k-mers*, originating for example from regions of the genome that are difficult to sequence. To estimate how many low coverage-*k-mers* are still true, we used a linear interpolation and calculated the area under the curve between the origin of the axes and the corresponding *k-mer* frequency at the cutoff of 5-fold coverage. This finally resulted

in 71% of *k-mers* predicted as true, most likely slightly overestimated. With this information and the total number of nucleotides that were used to calculate the distribution (85.42 Gb, Supplementary Table S1), the number of reads that make up the true *k-mer* distribution lies between 58.54 Gb (likely an underestimate due to the hard cutoff) and 60.69 Gb (which approximates the error-free *k-mer* distribution). To estimate the genome size we divided the number of nucleotides by the real sequencing depth (D = 37-fold) and obtained a range of 1.58–1.64 Gb.

The computed average coverage from corrected reads realigned to *AptMant0* (31.74-fold) and the total number of corrected reads (52.53 Gb, Supplementary Table S1) gave a similar genome size estimate of 1.65 Gb. Thus the kiwi falls at the higher end of the bird genome sizes distribution (Supplementary Table S4, http://www.genomesize.com) and *AptMant0* (1.595 Gb, Supplementary Table S3) covers about 96% of the expected genome length.

The AT5 individual was aligned to the genome reference using the same program and identical parameters. For this set of sequences, an average coverage of 21.5-fold was computed.

We further inquired whether the larger genome size is typical for kiwi, or rather a characteristic of the ratites. To this end we searched the Animal Genome Size Database (Supplementary Table S4), which approximates the genome size according to the species-specific C-value. According to this estimation, present ratites – ostrich and emu – are situated above the average for 896 birds. However, tinamou lies very close to the bird average. Since the ostrich and tinamou assemblies are available on GigaDB [17] we ran similar statistics as for the kiwi assembly (Supplementary Table S15). According to the assemblies ostrich and tinamou are situated in the avian range with genomes of 1.26 and 1.06 Gb lengths, respectively. However, no statistics are available for a *de novo* prediction of these birds' genome sizes. According to the Animal Genome Size Database,

ostrich has a diploid set of 80 chromosomes, with one additional chromosome as compared to the domestic chicken – 78 chromosomes.

Even by accounting the Ns in the kiwi assembly (13% Supplementary Table S3), the total size of the assembly would sum up to 1.38 Gb, still higher than the bird average. To roughly estimate whether the larger genome size in kiwi corresponds to coding or non-coding regions, we analyzed the scaffolds, which did not align to the chicken chromosomes. These represented 218,454 scaffolds with a total length of 167,802,248 bp. Of the 18,033 genes annotated in kiwi (Supplementary Note: *De novo* gene prediction and gene annotation), 22 genes (98,041 bp) were annotated on these scaffolds. Considering a uniform distribution of genes across the genome and scaffolds, the expected number of genes over 0.17 Gb is 1,916.

However, because gene annotation can be influenced by scaffold length, in a further analysis, we focused only on scaffolds with a length lower or equal to the maximal length of the scaffolds which were not aligned to the chicken genome (52,029 bp). We computed the average number of annotated genes according to length of scaffold in windows of 500 bp (i.e. average number of genes in for e.g. scaffolds with length of 10,000–10,500 bp). The same analysis was done on the scaffolds, which did not align and a Wilcoxon ranking test was used for statistical significance. P-value was highly significant (< 0.0001) for a lower number of annotated genes on the scaffolds that did not align to chicken genome. This suggests that the genome expansion is a characteristic of kiwi, rather than of the ratite clade, and in kiwi is likely a result of non-coding sequence expansion.

Measure for heterozygosity

Raw reads from each individual (kiwi code 73 and kiwi code AT5) were separately aligned to *AptMant0* using BWA (Version: 0.5.10) [36]. Each resulting data set was genotype called by Samtools mpileup version 0.1.18 [37] with “Base Alignment Quality”

computation turned off and otherwise default options. The likelihood function is obtained as the marginal likelihood of the data conditioned on heterozygosity, assuming independence of sites:

$$L(h) = \prod_{i} \sum_{x,y \in \{A,C,G,T\}} P(D_i|G_i = xy)P(G_i = xy|h)$$

$$= \prod_{i} h \sum_{x \neq y} P(D_i|G_i = xy) + (1-h) \sum_{x=y} P(D_i\,|G_i = xy)$$

where i ranges over all sites of the genome for which Samtools produced valid output, G_i is the (unknown) genotype at site i, D_i is the observed data at site i, and h is the fraction of heterozygous sites. The logarithms of the $P(D_i|G_i)$ values are, up to a factor not dependent on h, available from Samtools output in the PL field. We now define:

$$P_i = \frac{\sum_{x \neq y} P(D_i|G_i = xy)}{\sum_{x=y} P(D_i|G_i = xy)}$$

$$\pi = \log\frac{h}{1-h}$$

$$h = \frac{e^{\pi}}{1+e^{\pi}}$$

which replaces h with its log-odds-ratio, which is better behaved in the numerical optimization. By multiplying the previous term for the likelihood by $\frac{\sum_{x=y} P(D_i|G_i=xy)}{\sum_{x=y} P(D_i|G_i=xy)} = 1$ we obtain the expression that follows:

$$L(\pi) = \prod_i \sum_{x=y} P(D_i|G_i = xy) \prod_i \left(\frac{e^{\pi}}{1 + e^{\pi}} P_i + \frac{1}{1 + e^{\pi}}\right)$$

The first product does not depend on π, and can be dropped without changing the maximum of the likelihood function. We now take the logarithm, and arrive at the final log-likelihood function, which is maximized numerically using Newton iteration. An approximate 95% confidence interval is calculated from the second derivative:

$$\tilde{L}(\pi) \coloneqq \sum_i \log \frac{1 + P_i e^{\pi}}{1 + e^{\pi}}$$

$$\hat{\pi} \coloneqq \arg max_{\pi} \; \tilde{L}(\pi)$$

$$\pi_{\pm} \coloneqq \hat{\pi} \pm \frac{1}{\sqrt{\tilde{L}''(\hat{\pi})}}$$

For the first individual (code 73), we obtain a heterozygosity of 0.15% (CI from 0.1496% to 0.1501%), while for the second individual (code AT5), it is 0.21% (CI from 0.2120% to 0.2126%).

For the individual used to assemble the reference (code 73), the existence of sites, which are homozygous for an alternative allele, can be used as a rough measure of assembly error. The number of such sites was very low ($5.17x10^{-06}$).

Mitochondrial genome assembly

The similarity of genuine mitochondrial sequences and nuclear copies of mitochondrial (NUMT) DNA can cause bubbles in the *de Bruijn* graph, which results in conflicts for the assembly software. Consequently, we find several smaller contigs in our final assembly that align to the partial *Apteryx mantelli* mitochondrial genome reference (GeneBank AY016010.1) and cover parts of the sequence. Since only a partial *Apteryx mantelli* mitochondrial genome is available to date, to improve the mitochondrial assembly, the

mitochondrial genome was additionally assembled starting from the corrected reads of the short-insert-size paired-end libraries and using ‘AMOScmp-shortReads-new’ – a comparative assembler [38] being part of the Amos package (v.3.1.0). In contrast to *de novo* assembly, in this approach the assembly process is guided by a related mitochondrial genome.

Read filtering

The partial *Apteryx mantelli* mitochondrial genome reference (GeneBank AY016010.1), the complete *Apteryx haasti* (GenBank NC_002782.2) and the *Apteryx owenii* (GenBank NC_013806.1) mitochondrial genomes were used as proxy references to filter reads. To account for the differences between the sample kiwi code 73 and the references, as well as for sequencing errors, the filter must be relatively permissive. This was achieved by applying a spaced-seed filtering scheme:

Let R_1, ..., R_N be the references. Let further be sp=$[01]^l$ be a spaced seed pattern of length l and let the weight k be defined as the number of ‘1’s in the seed. Applying sp to a specific position p at R_i generates a seed $t_{Ri,p}$ of length k by aligning sp to R_i at p and concatenating the bases of r_i which show a ‘1’ in sp. Note that sp cannot be applied to a specific position, if at least one ‘1’ matches a ‘N’ or non ‘ACGT’ symbol. Applying sp to all possible positions (taking into account circularity of MT) of R_1, .., R_N as well as to its reverse complements generates the seed data base SDB[1].

Let r be a read. The pattern sp matches r at a position p, if the corresponding seed $t_{r,p}$ generated by applying sp at p is present in the seed data base SDB. The filter accepts r if sp matches r in at least 10% of the positions the pattern can be applied to or if the same holds for the reverse complement of r. Paired-end reads r1 and r2 are both accepted if at least r1 or r2 is accepted.

[1] example: seed pattern sp = 11011. sp has length l = 5 and weight k = 4. Let reference R = ”ACGTACGT”. Applying sp to R at position p = 3 generates the seed $t_{R,p}$ = GTCG

The spaced seed read filter was trained using the spaced seed pattern '1111011101111' (weight 11, length 13) on the mentioned references. After filtering

- 13.6 million (3.21%) reads from the 240-bp library
- 3.46 million (1.07%) reads from the 420-bp library
- 3.88 million (5.39%) reads from the 800-bp library

were retrieved and further used in the assembly.

First iteration:

Filtered reads of the 240-bp, 420-bp, and 800-bp corrected paired-end libraries were used. 'AMOScmp-shortReads-new' (http://sourceforge.net/projects/amos/files/) was run with default parameters using *Apteryx haasti* (GenBank NC_002782.2) mitochondrial genome as the reference. The output consisted of 6 scaffolds (14,872 bp, 1,302 bp, 344 bp, 323 bp, 266 bp, and 101 bp) with a total length of 17,208 bp, slightly bigger than the reference (16,980 bp).

Second iteration:

To further improve the assembly, the process was repeated. Reads were filtered by using the assembly retrieved by iteration one and a stricter seed pattern '1110111110111' of length 15 and weight 13 was applied. After filtering:

- 2.55 million (0.60%) reads from the 240-bp library
- 1.27 million (0.39%) reads from the 420-bp library
- 0.66 million (0.92%) reads from the 800-bp library

were used as input for the second iteration. This assembly consisted of 10 scaffolds with a total length of 18,377 bp and biggest scaffold of 14,896 bp. A second iteration did not further improve the assembly, hence the biggest scaffolds from this step were used for further designing primers to complete the mitochondrial genome.

PCR amplification of the missing fragment

To obtain the complete mitochondrial genome of *Apteryx mantelli* a forward primer was designed on the biggest scaffold from second iteration, with the sequence:

- CCAAAGACTGAAGAATACACCCC

and the reverse primer on the second biggest scaffold from second iteration, with the sequence:

- GGGAGCTGGAGGTAAAGGTT

A fragment of approximately 120 bp was amplified and sequenced using Sanger technology. The sequence filling the gap between the contigs was highly repetitive and high in GC content, suggesting that the original assembly failed due to low coverage and a complicated graph-structure representing the region.

RNA sequencing

RNA was extracted from tissue collected from the same female embryo (code 16-12) that was used for generating the longer-insert mate-paired-end libraries using standard procedures. Extraction yielded a high concentration of 111 ng/μL and good quality RNA (RNA integrity number = 5.5), which could be used for library preparation. Libraries with 250 bp-insert size were prepared from poly-A RNA using the Illumina TruSeq RNA Sample Preparation Kit v2. Sequencing was performed on the Illumina HiSeq Platform as described above and 47.5 Gb were generated.

De novo gene prediction and gene annotation

The MAKER pipeline [39] was used for gene annotation, based on several sources of evidence: *de novo* gene prediction, RNA-Seq data, and protein evidence from three species (*G. gallus, T. guttata, M. gallopavo)* downloaded from BioMart (Ensembl version 72 [18]).

RepeatMasker version 4.0.1 (http://www.repeatmasker.org) was used to identify repeats that matched to entries in standard databases for known repetitive sequences (i.e. Repbase 18.08 [40]), using data available for all the model organisms. Following repeat masking, preliminary gene models were produced using Augustus version 2.7 [41] with the training dataset for chicken, resulting in 27,876 *de novo* predicted genes. To increase the likelihood that a sequence region is associated with a gene, further evidence was sought from either homology to known proteins or the active transcription of that region. Briefly, kiwi RNA-Seq data was aligned to *AptMant0* using NCBI BLASTN version 2.2.27+ [5]. Given the fact that EST data usually contain just parts of transcribed RNA with only a few full length transcripts, BLASTX was used to align protein data against the raw genomic sequence in the attempt to identify regions of homology.

Using both the *ab initio* and evidence informed gene prediction, Maker updated features such as 5' and 3' UTRs based on RNA-Seq evidence and created a consensus gene set. 18,033 RNA-seq-supported kiwi genes were annotated according to this pipeline.

Orthologs and Ka/Ks calculation

Starting from chicken annotations, orthologs between chicken, zebra finch, and turkey were downloaded from Ensembl 73 [16]. Kiwi genes for which the Maker annotation ortholog assignment was in accordance to the triplet downloaded from Ensembl were considered in the further analysis. This resulted in 7,294 orthologs out of which 6,486 were single-copy.

For ostrich, tinamou, chuck-will's-widow, and barn owl orthologs were established by aligning coding sequences against the database consisting of chicken coding sequences from Ensembl 73using tblastx [28]. Orthologs transitivity is a major challenge in accurately assigning ortholog groups among multiple species. We thus filtered only the

reciprocal best hits to the chicken gene with an e-value ≤ 10^{-10} [42] and assigned it to the kiwi, chicken, zebra finch, and turkey ortholog group.

Coding sequences for the eight avian species were aligned using Muscle v3.8.31 [29]. Two different sets of alignments were compiled for further analysis:

1. The first set consists of alignments of all eight species that do not contain a single frameshift indel, yielding a set of 2,660 genes.
2. The second set consists of the longest uninterrupted run of at least 200 aligned bases in each multiple sequence alignment, for which we first assured that gaps in the alignment were not introduced by unresolved bases in our assembly. Adding these genes to the first set yielded a set of 4,152 genes.

The CODEML program from the package PAML [43] was run using first 3,754 orthologs assigned between kiwi, chicken, zebra finch and turkey, since the last three avian genomes are high quality, intensively-curated genomes and represent a quality control for the kiwi sequences. Six avian pairwise combinations were run to obtain estimates of non-synonymous (Ka) and synonymous (Ks) changes in the four avian lineages (*G. gallus, T. guttata, M. gallopavo, A. mantelli*). Ka and Ks distributions were compared pairwise between all four avian species (Supplementary Fig. S11). We found that the Ka values are much lower than Ks, confirming that non-synonymous mutations occur with a lower frequency, as a result of purifying selection acting to remove deleterious mutations. On the other hand, Ks values in kiwi were similar to the ones in the other bird lineages, most closely to the zebra finch distribution.

To examine selective constraints on the kiwi branch we scanned for differently evolving genes with the CODEML program under a branch model [9] using the 4,152 orthologs in the eight bird species. Unrooted trees used for phylogenetic analyses in CODEML were built on PHYLIP (http://evolution.genetics.washington.edu/phylip.html). Likelihood ratio tests (LRTs) were performed to compare a series of evolutionary models. First, an average ω was estimated across the tree using model = 0. The one-ratio model

(model = 0, NSsites = 0) was used to estimate the same ω ratio for all branches in the phylogeny. In a second step, we used the two-ratio model (model = 2, NSsites = 0), with a background ω ratio and a different ω on the kiwi branch. The same test was performed sequentially for each of the two nocturnal birds, ostrich, and tinamou as the foreground branch. These two models were compared via a likelihood ratio test (1 degree of freedom) [44]. In the last step the kiwi branch was fixed to ω = 1 and all other branches were considered different (model = 2, fix_omega = 1, omega = 1). The parameters used were default: CodonFreq = 2, kappa = 2, initial omega = 0.4. A chi-square test was used to test whether the kiwi branch is significantly different and the estimated ω for kiwi is higher than for other species, which would imply that these genes might be evolving faster on the kiwi branch. An identical test was performed then for chuck-will's-widow, barn owl, ostrich, and tinamou.

A similar test with kiwi as foreground branch was performed on the reduced dataset including only kiwi and the three Ensembl bird genomes. LRTs ($p < 0.05$) between the model in which kiwi represented the foreground branch (model = 2) and the null model (model = 0), in which all branches were considered equal (kiwi, chicken, zebra finch, turkey), predicted that 12.18% of the genes in this dataset show evidence for possible positive selection. This result is similar to previously reported values for chicken 10.17% and zebra finch 11.3% [45]. Misalignments and poor sequence quality can result in an inflated estimate of branch test positive selection [45, 46]. The comparison across species showed that the proportion of positively selected genes in kiwi is only slightly increased compared to high quality genomes, such as chicken and zebra finch.

Gene Ontology and rapidly evolving genes

Branch-specific ω values were used to identify GO categories that are evolving significantly different in each of the previously mentioned nocturnal birds and ratites, including kiwi. The ω values were estimated as described above, using all 8 avian

species. To explore the evolutionary functions that may have experienced faster evolution on each branch the GO annotation was downloaded from Ensembl 73 [16] and enrichment was tested using the FUNC package [19].

A hypergeometric test was run for each of the five bird species: kiwi, ostrich, tinamou, barn owl, and chuck-will's-widow by including in the set of interest genes with a significantly higher ω than the background. The same was done for genes evolving significantly slower. None of the species presented significantly enriched categories after family-wise error rate multiple testing correction. As an indicator for biological relevant processes, categories were considered for further analysis if there were 3 or more significantly changed genes in the node, and the number of significant genes was higher or equal to 5% of the total genes annotated in the node. By excluding the categories that appeared enriched in any of the other species we could identify the kiwi-specific GO categories and the ones shared with any other species. Supplementary Table S8 shows overrepresented categories enriched in genes faster evolving. We performed the same analysis for genes evolving slower in each of the considered bird species (Supplementary Table S8; Supplementary Table S9). Genes that clustered in biological meaningful GO categories, which could potentially underlie kiwi-specific physiology were extracted from the respective node to allow for manual inspection (Supplementary Table S8C; Supplementary Table S9C).

Gene families assignment using TreeFam

TreeFam defines a gene family as a group of similar genes, which descended from a single gene in the last common ancestor of the analyzed species [18]. The gene families were built across sixteen genomes: *Gallus gallus*, *Anas platyrhynchos*, *Ficedula albicollis*, *Meleagris gallopavo*, *Taeniopygia guttata*, *Pelodiscus sinensis*, *Anolis carolinensis*, *Homo sapiens*, *Mus musculus*, *Gasterosteus aculeatus*, *Ornithorhynchus anatinus*, *Tinamus guttatus*, *Struthio camelus*, *Antrostomus carolinensis*, *Tyto alba*, and *Apteryx mantelli*.

Except for *Apteryx mantelli* protein sequences were downloaded from Ensembl 73 [16] or GigaDB [17] (last four genomes – *Palaeognathae* and nocturnal birds) and the longest protein sequence was chosen for further analysis. Protein sequences from each species were searched against the TreeFam 9 HMM library.

TreeFam generates gene orthology and paralogy predictions using maximum likelihood phylogenetic gene trees. The HMM libraries are built using orthologs from 109 species. The gene trees reconciled with their species tree have their internal nodes annotated to distinguish duplication or speciation events [47]. Genes related to a speciation event are defined as orthologs, whereas those which arose through duplication events are considered paralogs.

HMMER [48] employed by TreeFam's software was used to assign homologs for the 12 considered species to existing TreeFam families.

In total, genes were assigned to 10,096 families, of which 623 contained one gene of each species. The single-copy orthologous families were used to build a phylogenetic tree for the sixteen species (Figure 1).

Gene families evolution using CAFE

Lineage-specific gene family expansion and contraction may cause differences in phenotype and physiology between species. To estimate the changes in gene repertoire in the kiwi, the most likely gene family size was inferred at all internal nodes of the TreeFam annotation for the sixteen species mentioned above. The expansion and contraction analysis of the orthologous protein families was performed by CAFE (computational analysis of gene family evolution) version 3.0 [2].

The method employs a stochastic birth-death process in gene family evolution, taking into account the phylogenetic tree topology and branch lengths. A UPGMA-rooted phylogenetic tree [49] was constructed for each of the 623 single-copy orthologs across the 16 species, as defined by TreeFam. Next, a majority-rule consensus tree was built

and the topology distance was calculated for each of the tree to the consensus using the ape package methodology in R [50]. Increasing the number of independent loci when building the consensus tree can lead to a partially unresolved species-tree topology [51]. R* consensus trees have been shown to be consistent estimators of species-tree topologies for any number of taxa with an increasing probability as the number of gene trees increases [51]. Since the R* consensus algorithm does not estimate branch lengths, a tree with minimum topologic distance to the consensus was considered for further analysis. Another assumption in the model [2] is that all families have an equal probability of changing over time from the initial state X_0 = s, to size c over time t; the probability for X_t = c is:

$$P(X_t = c \,|X_0 = s) = \sum_{j=0}^{\min(s,c)} \binom{s+c-j-1}{j-1} \alpha^{s+c-2j}(1-2\alpha)^j$$

where $\alpha = \lambda t \div (1 + \lambda t)$ and λ represents the birth death parameter. An initial family size $X_0 = 0$ results in a probability of 0 for birth and death. Therefore the initial lambda estimation was restricted to families in which $X_0 > 0$, excluding at first lineage-specific families. A global parameter λ was calculated using maximum likelihood, to describe gene birth and death across all branches in the tree for all gene families. The estimated λ was 0.0007 when all 16 species were included, lower than estimations from previously published studies, which included only Neoaves and estimated λ to 0.0011 [52, 53]. Exclusion of the night birds and the two *Palaeognathae* – ostrich and tinamou lead to a λ of 0.00104844, similar to the studies, which had included only Neoaves, suggesting that the λ calculation is consistent with previous reports.

Gene gains and losses were considered for families with a conditional p-value < 0.01 and with a significantly different kiwi branch. If the size of the kiwi branch gene family was larger than the ancestral family, the event was considered an expansion and the

opposite was a contraction. The average expansion on the kiwi branch was 0.272, while ostrich and tinamou had a net overall contraction -0.160 and -0.052, respectively. Of the birds, the only ones showing overall expansions were chicken (0.027), duck (0.007), and zebra finch (0.196).

In total on the kiwi branch 2,046 gene families were expanded, 822 were contracted. Over both categories 130 were significantly different from other lineages. For the families, which were significantly expanded in kiwi (Viterbi p-value < 0.05) we manually verified the genes clustering in the family. If two genes (A and B) clustered in the same TF family and were annotated on the same scaffold with coordinates in close proximity, we inspected the scaffold between the coordinates separating gene A and gene B. If stretches of Ns were detected, the predicted gene A and B were considered fragments of the same gene. After manual curation, 85 of the initial 130 TF families were still significant. Supplementary Table S6 shows the gene families significantly changed on the kiwi branch with a Viterbi p-value lower than 0.05.

To check whether significant contraction/expansion events cluster in different GO categories we tested enrichment using ClueGO with a hypergeometric test [3]. Briefly, Pfam IDs corresponding to the TreeFam families were assigned to GO categories. We separately considered the significantly contracted families and the expanded families as the test dataset and measured against the background set of the chicken GO annotations. Categories belonging to biological process with a p-value lower than 0.0001 were grouped in functional networks and the non-redundant GO categories are shown in Supplementary Fig. S2.

Detection and classification of the membrane proteome

For comparison with kiwi, complete protein sequence sets for the following bird and reptile species were downloaded from Ensembl 74 [16]: *Taeniopygia guttata, Meleagris gallopavo, Ficedula albicollis, Anas platyrhynchos, Pelodiscus sinensis, Gallus gallus,* and

Anolis carolinensis. Homo sapiens from the same Ensembl version was used as outgroup. To detect more reliably kiwi-specific changes the membrane proteome was annotated additionally in two nocturnal birds – barn owl and chuck-will's-widow and two ratites – ostrich and tinamou sequenced to date [54]. The protein data set was filtered by only including the longest protein sequence for each gene. Membrane proteins and signal peptides were predicted for kiwi and the additional eleven birds and reptiles with Phobius [55].

The predicted membrane proteins were classified based on a manually curated human membrane proteome dataset, which describes family relationship and molecular function. The predicted membrane proteins were aligned to the human membrane proteome dataset with the BLASTP program of the BLAST package using default settings (v. 2.2.27+). Each predicted membrane protein was classified according to its best human hit with an e-value < 10^{-6}. Predicted membrane proteins with no hit were deemed unclassified, along with those proteins that hit an unclassified human protein.

There were no significant differences in the total number of predicted transmembrane proteins or in the ratio of classified to unclassified proteins in the kiwi genome in comparison to the other high quality bird and reptile genomes in Ensembl 74 [16] (Supplementary Table S7). While the ratio of classified to unclassified proteins was higher in the four genomes from the study of Zhang et al. [54], the total number of predicted proteins was consistently lower than in the other annotated genomes. However, since most of the annotated proteins could be classified, the present assemblies generally provide a reasonable substrate for further analysis.

CAFE [2] was employed as previously described to check for expansion and contraction events in the analyzed membrane proteomes. The calculated birth and death on the 13 species constructed phylogeny was 0.0009. On average in kiwi there was an expansion (average expansion parameter was 0.505), the same in tinamou (0.115) and the ratite node (0.051), while ostrich showed on average contraction (-0.614). Expansions

happened in song birds (0.268) and *Galloanserae* (0.108), while nocturnal birds showed contraction (-0.468).

We validated that expanded families from the CAFE analysis are not caused by overestimation of gene counts due to fragmentation. This was done by alignment of human and kiwi proteins in each family with MAFFT (v7) using the option E-INS-I with default settings (an iterative refinement method that uses the weighted sum-of-pairs score and consistency score from local alignments with a generalized affine gap cost). The resulting alignments were used to calculate a preliminary maximum-likelihood phylogenetic tree for each family with FastTree (v2,1,8) [56] using the WAG substitution model and default settings. In each tree, instances where two or more kiwi proteins clustered with one human protein were analyzed in the alignments to determine whether the kiwi proteins aligned to different parts of the human protein which would indicate fragmentation rather than true duplications of the gene. After validation the gene count for each family was corrected accordingly. We validated 18 families that were significantly expanded in kiwi but not in *Palaeognathae* and found that 11 were affected by fragmentation. The fragmentation of genes was caused both by erroneous gene predictions and unresolved bases in the assembly resulting in gene-split onto different scaffolds.

After the number of genes was manually adjusted, CAFE analysis was rerun and significant changes on the kiwi branch are shown in Supplementary Table S7.

Phylogenetic analysis

Nuclear loci phylogeny

The 623 single-copy orthologs (862,710 bp) identified using TreeFam in the 16 species mentioned above were used to build a consensus phylogeny from a nuclear perspective. Genes were aligned against each other using Muscle [29] version 3.8.31. The resulting alignments were loaded in PAUP* [10] version 4.0d105 and trees were inferred using a

maximum likelihood criterion, with default parameters and the Hasegawa-Kishino-Yano [57] nucleotide substitution model. To measure the confidence for the tree, a series of 100 bootstrap replicates were performed (Figure 1). All nodes had 100% bootstrap support.

To further check the position of tinamou in the *Palaeognathae* clade, we additionally used an extended dataset of coding sequences from 3,939 orthologs (14,104,428 bp) in 8 bird species: kiwi, ostrich, tinamou, turkey, chicken, zebra finch, chuck-will's-widow, and barnowl. Multiple alignments and trees were built using the same approach as described above. All nodes had 100% bootstrap support (**Error! Reference source not found.**A). We furthermore inquired the bird phylogeny from birdtree (http://birdtree.org/) on the same species: *Apteryx mantelli*, *Struthio camelus*, *Tinamus guttatus*, *Gallus gallus*, *Meleagris gallopavo*, *Taeniopygia guttata*, *Tyto alba*, and *Caprimulgus carolinensis* using first the Ericson (**Error! Reference source not found.**B) and then Hackett backbone (**Error! Reference source not found.**C) [11] with 100 generated trees. Both phylogenies agreed with the topology of our tree, while in the Hackett et al. study [58], ostrich is presented as basal.

Ultra-conserved non-coding elements phylogeny in birds

Ultra-conserved non-coding elements were downloaded from UCNEbase [12] and orthologous regions in *Apteryx mantelli, Struthio camelus, Tinamus guttatus, Tyto alba, Antrostomus carolinensis*, *Ficedula albicollis, Taeniopygia guttata, Anas platyrhynchos*, and *Meleagris gallopavo* were established as described in Supplementary Note: Ultra-conserved non-coding elements analysis.

3,076 UCNEs had a length of at least 95% of the chicken reference UCNE in all investigated genomes. We used this set (1,011,462 bp) to build a bird phylogeny [10]. The alignments were loaded in PAUP* [10] version 4.0d105 and trees were inferred using a maximum likelihood criterion, with default parameters and the Hasegawa-

Kishino-Yano [57] nucleotide substitution model. To measure the confidence for the tree, a series of 100 bootstrap replicates were performed. All nodes had 100% bootstrap support.

Our results show different positions in the tree of the tinamou, according to the conservation level of the DNA sequence used (**Error! Reference source not found.**; **Error! Reference source not found.**) and suggests that the 19 loci used across the 169 species in the Hackett et al. study [58] could have been highly conserved to facilitate their amplification across multiple species.

Mitochondrial phylogeny

To compute the mitochondrial phylogeny, mitochondrial genome sequences from various species (Supplementary Table S17) were pooled alongside the previously described kiwi mitochondrial genome assembly. Sequences were aligned using prank [59] v 0.111130. Again, trees were inferred from aligned sequences loaded in PAUP* [10] under a maximum likelihood criterion, using the same parameters described in the section above and a 100 bootstrap replicates were performed (Supplementary Fig. S8A). Mitochondrial alignments were further analyzed using BEAST v1.6.2. An uncorrelated relaxed clock model with log-normal distribution and mean of $2.4x10^{-9}$ was used and the GTR model was employed for the site model. A prior on the TMRCA for the Emu and Cassowary of $2.55x10^{7}$ years and a standard deviation of $5.5x10^{6}$ was set according to [60]. A total of 10 million MCMC chains were performed, with sampling at every 1,000 chain to produce a tree. The effective sample size (ESS) was calculated for the input parameters. The TMRCAs had an ESS above 100, a minimum threshold recommended by the software manual (http://www.beast2.org/wiki/index.php/Increasing_ESSs accessed 10/20/2014). The first 1,000 out of 10,000 trees were considered burn-ins and discarded from the analysis. Divergence times are presented in Supplementary Fig. S8B.

The mitochondrial phylogeny shows a split between the North Island and South Island kiwi species about 10 million years ago. The resulting tree shows that despite their common geographical location, New Zealand ratites (kiwi and moa) are not monophyletic [61-64] (Supplementary Fig. S8). Our phylogeny provides good support for kiwi as sister group to the extinct Madagascan elephant bird (bootstrap value of 100%) and the extant Australian ratites (emu and cassowary) (bootstrap value of 93%), although the topology of other branches is still unstable, as shown by the lower bootstrap values. Making inferences about phylogeny, speciation, divergence times, and conservation from mitochondrial DNA data alone has been previously reported to be susceptible to errors [65]. Ostrich still has an unstable position in the phylogeny (Supplementary Fig. S8A) [62]. In the case of tinamou, the tree was thought to be solved by introducing nuclear data [61], while our phylogenies still show conflicting topologies (Supplementary Fig. S6; Supplementary Fig. S7). Hence, to securely determine ratite phylogeny mitochondrial DNA alone is insufficient and nuclear, ideally whole genome data would be required from more ratite species.

Vision analysis

Rhodopsin genes identification and annotation

The Pfam database was locally installed (version 26) and all bird and reptile proteomes (Ensembl release 74 [16]), as well as two ratites (*Tinamus guttatus*, *Struthio camelus*) and two nocturnal birds (*Antrostomus carolinensis*, *Tyto alba*) (GigaDB [17]) were searched against it using the Pfam_scan.pl script obtained from the Pfam ftp-site (ftp://ftp.sanger.ac.uk/pub/databases/Pfam/Tools/) with default settings. In Pfam, the *Rhodopsin* family of GPCRs is represented by a specific hidden Markov model [Pfam: PF00001 or 7tm_1]. All sequences that were assigned to the 7TM_1 (PF00001) HMM profile in the Pfam-search were considered to be homologues of the *Rhodopsin* family and were included for further subfamily classification and annotation. The script

Pfam_scan.pl uses a homology criterion set by the Pfam database, which is based on a manually curated gathering threshold for each model. The gathering threshold of a Pfam model makes sure that any sequence must attain a score greater than or equal to the threshold to be deemed significant. The gathering threshold of the 7TM_1 (PF00001) HMM model is a score of 23.8 which equates to an e-value of approximately 0.01.

To assign subfamily level classification for the identified *Rhodopsin* class receptors, a standalone BLASTP search against the human GPCRs was performed. BLASTP searches had standard default settings, with a word size of 3 and BLOSUM62 scoring matrices. The *Rhodopsin* class GPCRs from the human GPCR repertoire were downloaded and tagged with the subfamily categorization. The *Rhodopsin* class receptors from bird and reptile genomes were then searched against the database consisting of the tagged human *Rhodopsin* GPCRs. If at least four of the five best hits were in the same subfamily after the BLASTP search, sequences were assigned to it.

Additional results for the vision analysis

OPN1MW

To validate the missense mutation in codon 134 (transition of G to A in the kiwi branch), which leads to the exchange of Glu^{134} with Lys in transmembrane helix 3, DNA was extracted from additional *Apteryx* species *rowi* and *haasti.* To check whether the mutation is also present in other ratites the *OPN1MW* fragment was sequenced in the following species: Cassowary, Emu, Rhea, Tinamou, Eastern moa, and Mappin's moa.

Moa bone samples CM Av8365 (Eastern moa) and W336 (Mappin's moa) were kindly provided by Canterbury Museum and Whanganui Regional Museum, respectively. North Island Brown, Okarito Brown, and Great Spotted kiwi feathers were kindly provided by Rainbow and Fairy Springs, Rotorua; Chris Rickard, Franz Joseph Department of Conservation; and Melanie Nelson, Arthur's Pass Community Roroa Recovery Project, respectively. Emu and rhea DNAs were provided by Joy Halverson, Zoogen, California.

DNA was extracted from moa bone and kiwi feathers bases following the procedures outlined in McCallum et al. [47, 66] and Hartnup et al. [4, 67], respectively.

2 µL of DNA extract (~2 – 10 ng) was amplified in a 10 µL volume containing 50 mM Tris-Cl, pH 8.8, 20 mM $(NH_4)_2SO_4$, 2.5 mM $MgCl_2$, 1 mg/ml BSA, 200 µM of each dNTP, ~0.3 U of Platinum Taq, 0.5 µM of each primer (rhFF, 5'agtcgacgcttctagcttCCTGTGGTCCCTGGT; rhR, 5'GATGCCCATCATGGCGT). A generic sequencing primer was added to the forward primer (lower case) to allow single pass sequencing. The mix was subjected to 94°C 2 min (x1) then (94°C for 20 sec, 58°C for 1 min) for 43 cycles using an ABI GeneAmp System 9700. Amplified fragments were detected by agarose gel electrophoresis in 0.5 × Tris-borate-EDTA buffer (TBE), stained with 50 ng/mL ethidium bromide in TBE, and then visualized over UV light. Amplified products were purified by centrifugation through ~100 µL of dry Sephacryl S200HR, then sequenced at the Griffith University DNA Sequencing Facility (Australia) using Applied Biosystems (ABI) BigDye Terminator v3.1 chemistry and an ABI3730 Genetic Analyzer. Sequences were visualized and edited in Sequencer.

OPN1MW was also annotated in *Tinamus guttatus*, *Struthio camelus*, *Antrostomus carolinensis*, and *Tyto alba* genomes. The deleterious mutation was present in all sequenced kiwi samples and absent in other ratites, which all presented an ERY motif (Figure 2).

OPN1SW

To validate the missense mutation, which leads to the substitution of $Glu^{6.30}$ with Gly in the kiwi *OPN1SW*, aligned reads from both individuals AT5 and kiwi code 73 to *AptMant0* were inspected. This confirmed the presence of the mutation in both individuals with support from all aligned reads. *OPN1SW* is absent in the *Struthio camelus*, *Antrostomus carolinensis*, and *Tyto alba* assemblies as suggested by the missing *OPN1SW* annotation in those genomes, and by the lack of either reliable reciprocal blast

hits to the zebra finch SWS1 transcript ENSTGUT00000016824 or the Pfam domain-based annotation. Only a partial sequence could be identified in the *Tinamus guttatus* genome (Tma_R005000, which had been wrongly assigned to Source = ENSTGUT00000010973; Function = "RHO-2" in the original annotation). While partial coding sequences from *Caprimulgus europaeus* (AY227187.2) and *Struthio camelus* (AY227189.3) could be retrieved from GenBank, these did not correspond to the TM6 sequence present in kiwi and could not be used to either verify the mutation, or for following selection analysis.

Ka/Ks analysis

To check whether the opsin evolutionary rate is kiwi-specific, or shared with either nocturnal birds, or other ratites, selection analysis was performed by sequentially appointing kiwi, barn owl, chuck-will's-widow, ostrich, and tinamou as foreground branch (Supplementary Table S10). The branch model was run using CODEML package [9] with parameters model = 2, fix_omega = 1, for the null model, and fix_omega = 0, for the alternative model. LRTs were calculated between the two models and also using the null model in which all branches were considered equal (model = 0). The first comparison (model = 2 vs. model = 2 with fixed omega to 1) tests whether Ka/Ks in the foreground branch is significantly higher than 1. The second comparison (model = 2 vs. model = 0) tests whether a model which assumes a different omega in kiwi than in the set of all other branches in the tree is has a higher likelihood than the one where one omega is estimated for all sites and branches in the tree.

We observed a faster evolution in *RHO* gene in both kiwi and chuck-will's-widow, while tinamou showed a slower evolutionary rate. However, this gene does not evolve neutrally in either of the tested species. For *OPN1MW* and *OPN4-2* the faster evolution was kiwi-specific. Unlike *OPN1MW*, *OPN4-2* seems to evolve neutrally in kiwi.

To test whether opsin inactivation in kiwi is either a result of positive selection or loss of constraint, a branch-site analysis was performed using estimates from CODEML [9] with parameters model = 2, NSsites = 2, fix_omega = 1, for the null model, and fix_omega = 0, for the alternative model, and otherwise default parameters. For the *OPN1MW*, the alternative model was not a better fit. The same model was run for the *OPN1SW* fragment, TM6 and TM7 and this resulted in no positively selected sites, although the branch model predicted a significantly faster evolution on the kiwi branch ($\omega_{kiwi} = 0.192$, $\omega_{other\ birds} = 0.013$, p-value < 0.005). A branch-site test was run for *RHO* and *OPN4-2* and neither showed any positively selected sites.

To estimate the period of time for which the loss of constraint has been operating we made the following assumptions:

- Before the loss of constraint happened on the kiwi branch $\omega_{1kiwi} = \omega_{background\ branches}$
- The loss of constraint happened in a short period of time, after which no selective force acted on the gene rendering $\omega_{2kiwi} = 1$
- The observed $\omega_{3kiwi} = \frac{a*\omega_{1kiwi}+b*\omega_{2kiwi}}{2}$, where a is the period of time before the loss of constraint, b is the period of time after the gene evolved neutrally and a+b = 1. Hence $b = \frac{2\omega_{3kiwi}-\omega_{1kiwi}}{1-\omega_{1kiwi}}$.

Using the above model, and calculating ω on *OPN1MW* and *OPN1SW* with model = 2 with kiwi appointed as foreground branch [9] (Table 2), and the split time between kiwi and the background branches (ostrich – 53 million years – for *OPN1MW* (Supplementary Fig. S8B); chuck-will's-widow – 105.9 million years – for *OPN1SW* (http://www.timetree.org)) the estimated time when the loss of constraint happened on the vision opsins is 38 million years for *OPN1SW* and 30 million years for *OPN1MW*.

GO significant categories

Using the previously described ranking of faster evolving genes on the kiwi branch (section "Gene Ontology and rapidly evolving genes"), we searched for vision related GO categories which showed enrichment and extracted genes which clustered in the nodes with a significantly different evolution in kiwi (Supplementary Fig. S8C).

Of the presented genes, the one mostly related to bird physiology is *cOpn5L2*, a mammalian type of neuropsin described as an ultraviolet-sensitive pigment of the retina and other photosensitive organs in birds [68]. The faster evolution of this gene could well be related to the faster evolving *OPN4-2* (**Error! Reference source not found.**; Supplementary Table S10) and a potential function in the photoperiodic gonadal regulation in kiwi [69].

Olfaction analysis

Olfactory receptor genes identification and annotation

To compile a comprehensive set of olfactory receptors (ORs) in kiwi, both the Augustus *de novo* gene prediction and the Maker guided gene prediction output datasets were searched. Scaffold positions of the prediction/annotation were then checked and redundant sequences were removed. Four steps were performed to annotate ORs:

1. 211 functional ORs from the chicken genome [70-72] were downloaded and aligned against the kiwi transcriptome using TblastN with default parameters (word size 3 and Blossom 62 scoring matrix). After collecting overall hits for each query, identical (same) hits from each run were removed to obtain a non-redundant dataset.
2. A Pfam search against the kiwi proteome, with a default e-value cutoff of 1.0 was used to identify sequences that contained 7tm_4 domain (olfactory domain).

3. The 7tm_4 domain was searched against the kiwi proteome by a CDD search (conserved domain database search). A CDD search with default search parameters may contain few non-specific hits and lead to false positives. The false positive-matches to proteins that were not ORs were later excluded, in accordance to a phylogenetic analysis where non-ORs clustered outside of the OR variation.
4. Separate HMM profiles were built from conserved 7tm regions of functional ORs of chicken (211 sequences) and zebra finch (137 sequences) obtained from previous studies [70-72]. Also, a separate HMM profile using turkey ORs (80 sequences) was built. Using the three HMM profiles, HMM searches were performed against the kiwi proteome and non-redundant hits were retrieved from combined results of all three searches.

Sequences obtained from all of the above steps still included several redundant hits, pseudogenes, as well as non-ORs. A CD-HIT (Cluster Database at High Identity with Tolerance) was performed to remove identical sequences with a cut-off of 100%. On the final dataset of 402 sequences preliminary phylogenetic analysis was performed using the MEGA (version 6) software package [73, 74] and a maximum likelihood (ML) approach. Non-ORs were removed if they clustered separately from ORs with a high confidence value. Furthermore, non-ORs were cross-verified using BlastP, Pfam, and CDD search.

For the kiwi ORs, we excluded pseudogene candidates if at least one premature stop codon and/or frameshifts could be identified in the kiwi sequence. After the removal of redundant hits, pseudogenes, and non-ORs, we were able to annotate 88 unique OR sequences in kiwi. See flowchart Supplementary Fig. S12.

ORs are highly duplicated in vertebrate genomes. This property can be exploited to gain a second estimate of the OR repertoire in kiwi based on genomic coverage. Our method follows these steps:

1. The kiwi hits were extracted according to their genomic position;
2. All sequences were manually curated and fragment sequences were combined into one predicted gene if they belonged to the same query protein;
3. Coverage in the genomic area was calculated using samtools mpileup version 0.1.18 [37] on the BWA alignment of the short-insert-size libraries to the assembled genome;
4. Coverage was normalized to the average genome size coverage according to the GC content of the OR receptor, obtaining an estimate of fold increase in comparison to the genome average.

After coverage correction a total of 141 OR genes were estimated in the kiwi genome, of which 86 were full length ORs and 55 represented pseudogenes because of frameshifts, premature stop codons or truncations.

Comparative phylogenetic analysis on ORs from kiwi and other bird and reptile genomes

Refinements of the genome sequences quality and annotations can impact the estimates of gene families' sizes [75]. Thus, we downloaded the most recent proteomes to date of all bird and reptile genomes from Ensembl 74.

Using the procedure mentioned above, the OR gene repertoire was estimated in all bird and reptile genomes for comparative phylogenetic analysis with the kiwi OR dataset. All obtained OR genes were then aligned using MAFFT (v7) (http://mafft.cbrc.jp/alignment/server/) [76, 77], with BLOSUM62 as the scoring matrix and using default settings of option E-INS-I. Only the transmembrane regions were considered for phylogenetic trees and sequences with long gaps due to the lack of one or more transmembrane regions were removed. Phylogenetic analysis was run using both ML and neighbor joining (NJ) approaches as implemented in the MEGA (v5.2) software package. For all the ML and NJ trees constructed for the OR analysis, we utilized Jones, Taylor, and Thornton (JTT) as the amino acid substitution model. All sites

were considered for estimating phylogenetic inference. For all trees, a non-parametric bootstrap analysis was run for 500 replicates and a consensus tree was obtained using the replicates. The phylogenetic trees obtained from both approaches were drawn in FigTree 1.3.1 (http://tree.bio.ed.ac.uk/software/figtree/) (Figure 3).

Additional results for the olfaction analysis

ORs protein structure

ORs are 300-350 amino acids long and contain structural features common to GPCRs such as seven hydrophobic transmembrane domains (TM), a potential disulfide bond between the highly conserved cysteines in extracellular loops 1 and 2 and some conserved short sequences [78]. To classify a 7TM sequence as OR, several specific features should be tested, such as the unusually long second extracellular loop2 (EC2) and conserved amino acid motifs like LHTPMY in intracellular loop1 (IC1), MAYDRY at the end of TM3 and beginning of IC2, SY at the end of TM5, FSTC at the beginning of TM6 [79]. An amino acid sequence logo was generated from the Muscle-alignment of the OR paralogs in all birds and reptiles, using the WebLogo program [14]. The annotation of the kiwi OR repertoire and the interspecies variation level were checked (Supplementary Fig. S9). The logos illustrate the sequence conservation of ORs and notably avian ORs show higher conservation in comparison to turtle and green anole (indicated by fewer and larger letters at individual positions in the logo). Interestingly, the MAYDRY motif at the end of TM3, previously reported to be a signature motif for mammalian ORs [80], is highly conserved in kiwi, nocturnal birds, ostrich, chicken, and duck, while song birds and tinamou show higher variation. TM3, TM4, and TM5 contain hypervariable regions involved in odorant molecules recognition [79]. We identified four of the OR-specific conserved regions. The variable regions and also the higher conservation level in the avian clade compared to reptiles are depicted in Supplementary Fig. 9. The higher conservation level in *Aves* compared to reptiles can be

a result of the γ-c expansion in birds, as these receptors have highly similar sequences [71, 81].

γ-c clade ORs within-species protein sequence entropy

To check for within-species sequence similarity in the γ clade of the Shannon entropy (H) was calculated using the function implemented in the BioEdit program [47] with the following equation:

$$H = -\sum_{i=1}^{M} P_i \log_2 P_i$$

where P_i is the fraction of residues of amino acid type *i*, and M is the number of amino acid types (20) [70]. H ranges from 0 (only one residue is present at that position) to 4.322 (all 20 residues are equally represented in that position). $H \geq 2.0$ is considered as a variable position, whereas $H \leq 2$ as conserved. Highly conserved positions are those with $H \leq 1.0$ [82]. To ensure that H is calculated over homologous amino acid positions, the γ clade ORs were aligned with the Muscle program [29] pairwise between bird species. Gaps were excluded and H was averaged across all positions for each of the two species separately. Average entropies were 1.231 ± 0.158 – kiwi, 1.105 ± 0.127 – chicken (Ensembl 74 orthologs and 0.732 ± 0.07 for ORs from the study by Steiger et al. [71]), 1.022 ± 0.149 – duck, 1.086 ± 0.045 – flycatcher, 1.067 ± 0.081 – turkey, 0.340 ± 0.056 – zebra finch, 0.893 ± 0.119 – tinamou, 0.694 ± 0.081 – ostrich, 1.067 ± 0.130 barn owl, and 1.082 ± 0.147 – chuck-will's-widow. The difference in entropy calculations in the chicken OR repertoire between the Ensembl 74 annotations and the Steiger et al. study [71] supports the curation of the dataset in the newer Ensembl release, by removing redundant sequencing artifacts. A Wilcoxon rank test was performed using the average H estimates in kiwi against all other tested birds. This confirmed the significant difference with kiwi showing a higher variation in γ clade OR (p-value = 0.003).

Limb development analysis

Wing development genes orthologs were assigned among four bird species: kiwi, chicken, zebra finch, and turkey to study the conservation of these genes on each lineage (Supplementary Fig. S3; Supplementary Table S12). Corresponding orthologs were aligned [29] and multiple alignments were translated and manually inspected. No obvious alterations could be identified in the inspected genes.

Selection analysis on limb development genes

Sequences were aligned in kiwi and at least three of the following bird species: chicken, zebra finch, turkey, flycatcher, duck, falcon, and rock dove. Evidence for selective pressures was evaluated under the branch models implemented in CODEML [9]. We tested branch models, the most simple (model = 0, one ratio) of which admits a single ω ratio for the entire tree and the more general (model = 2, two-ratio), which allows a different ω ratio for the tested kiwi branch. These two models were compared via a likelihood ratio test (LRT). The level of significance for these LRTs was calculated using a chi-square approximation given that twice the difference of log likelihood between the models ($2\Delta lnL$) will asymptotically have a χ^2 distribution, with a number of degrees of freedom corresponding to the difference of parameters between the nested models (in this case, 1 degree of freedom [44]).

Hox cluster annotation

The vestigial wings of *Apteryx* suggest that limb development differs in this bird and changes in the HOX gene clusters may constitute the genetic cause. Therefore, we analyzed the HOX gene clusters in more detail. The workflow of the analysis is summarized in Supplementary Fig. S4A. We first identified the scaffolds and isolated contigs harboring (putative) *HOX* genes by blast [5] and then mapped all 673 sauropsid

HOX protein sequences from GenBank [6] for which cluster membership and paralog group were annotated to these scaffolds/contigs. The translation of the *Apteryx* sequences was compared to the database protein sequences by means of clustalw alignments and subsequent manual inspection. We found all 39 *HOX* genes expected for the Sauropsid ancestor [8] (Supplementary Fig. S4C). Furthermore, we observed that the *Apteryx* HOX protein sequences are virtually identical to those of other birds, in particular *Galliformes*. Thus, there are no indications that changes in the protein sequences might explain wing morphology in *Apteryx mantelli.*

We therefore proceeded to investigating the regulatory sequences within the HOX clusters by phylogenetic footprinting, i.e., we asked whether ancient, conserved DNA elements were preferentially lost in *Apteryx mantelli* compared to *Galliformes*. To this end, we used tracker2 [7], a software tool that was previously used to investigate the evolution of non-coding DNA elements in HOX clusters. In brief, the tool first computes pairwise local sequence alignments of non-coding sequences with high sensitivity from the syntenic regions anchored by the delimiting *HOX* genes, combines these to overlapping local clusters, and then uses the co-linear arrangement of local footprint clusters as a filter to increase the specificity. This last step is sensitive to local assembly errors. We therefore compared the *Apteryx mantelli* HOX cluster sequences with the sequences of the homologous HOX clusters from *Gallus gallus* and *Anas platyrhynchos* (Ensembl 74). To minimize false positives, i.e., an overestimation of footprint loss, we removed duplicate sequences from the *Apteryx* HOX clusters, arranged HOX clusters distributed on multiple scaffolds/contigs into a single sequence, and changed the order of a few small contigs to match the order observed in the other birds. Although we cannot rule out that the apparent rearrangements in kiwi are real, we interpret them as assembly problems (i) because they are flanked by accumulations of undetermined sequence positions ("N") and (ii) all rearrangements are order preserving (i.e., there are only transpositions but no reversals). The edited sequences together with the *HOX* gene

positions are available at http://www.bioinf.uni-leipzig.de/~studla/KIWI-HOX/ and https://bioinf.eva.mpg.de/KIWI-HOX/.

Although the distances between the *HOX* genes in the *Apteryx* sequences in terms of coordinates appear to be expanded by a factor of about 1.5 relative to other birds, this effect is entirely explained by the size of inserted blocks of Ns. Uninterrupted intervals of *Apteryx* sequence very closely match the length of the homologous sequence intervals in the duck and chicken HOX clusters. Within the accuracy allowed by the genome assembly *AptMant0*, the size of the kiwi HOX clusters closely matches their counterparts in duck and chicken, with no observed major insertions or deletions. Counts of conserved phylogenetic footprints are therefore directly comparable between chicken, duck, and kiwi.

Ancestrally present footprints are determined from five outgroup species: a shark (either hornshark *Heterdontus francisci* or Elephant shark *Callorhinchus milli*), the basal actinopterygian *Polypterus senegalus* (HOXA only), the coelacanth *Latimeria menadoensis,* the frog *Xenopus tropicalis* (HOX B, C, D), and human. Two ingroups, chicken and duck, were used to compensate at least partially for the less than perfect state of the genome assemblies of both chicken (Galgal4) and duck (BGI duck 1.0). For chicken HOXA, HOXB, and HOXC we used HOX cluster sequences which were improved by manual curation and additional sequencing [83]. Given the incompleteness of some of the genome assemblies, in particular the HOXC clusters of birds, and some differential losses of *HOX* genes in early vertebrate evolution that also affected the adjacent non-coding sequences, only the HOXA clusters could be assayed in full length, while the analysis of the other three clusters, HOX B, C, and D, had to be restricted to the core regions HOXB9-HOXB1, HOXC13-HOXC8, HOXD12-HOXD3 indicated by gray background in Supplementary Fig. S4C. Footprint losses are summarized in Supplementary Table S11. Since footprints (i.e., functional elements) tend to be retained or lost as entities, nucleotide counts cannot be treated as Poisson variables (see e.g.

[84]). Statistical significance of footprint count differences thus is evaluated directly in terms of the footprint counts, or by dividing the total length of lost footprints by the average length of conserved elements.

Our analysis clearly does not support an accelerated loss of footprints in *Apteryx mantelli*. The alternative hypothesis of a reduced loss (and hence a more ancestral state) of the HOX clusters in *Apteryx* compared to *Galliformes* and songbirds is a tempting hypothesis, but also not significantly supported by the available data. We thus can only conclude that the reduced morphology of the kiwi's wings is not reflected in a dramatic restructuring of HOX gene clusters. A deeper analysis of possible involvement of HOX gene regulation will require improved sequencing of HOX cluster sequences not only in *Apteryx* and preferably additional ratites, but also in song birds and *Galliformes*.

Fibin **identification and selection analysis**

Fibin, one of the genes involved in forelimb development [85] (Supplementary Fig. S3), could not be identified among the annotated genes. *Fibin* sequences for the following species *Bos taurus, Cavia porcellus, Chinchilla lanigera, Ficedula albicollis, Gallus gallus, Heterocephalus glaber, Melopsittacus undulatus, Octodon degus, Ovis aries*, *Pseudopodoces humilis,* and *Taeniopygia guttata* were downloaded from GenBank [6]. Additionally, we used the annotated *fibin* in *Struthio camelus* provided by Leon Huynen (unpublished data). TblastX from the BLAST package [5, 28] was used to align the sequences to *AptMant0.* This resulted in 4 scaffolds containing blast hits with e-values lower than 10^{-20}. After close inspection of all hit regions, 3 of the 4 scaffolds were discarded, as aligned regions were short (20-30 bp) and had low complexity. Scaffold87: 19,476,180-19,476,716 presented reliable hits, with the 537 bp mapping on the forward strand to the 3’ UTR of the *Gallus gallus fibin*. Given the observed results the assembly was inspected further. A gap of 2,350 bp is present on scaffold87: 19,473,482-19,475,763, upstream of the fibin 3’ UTR match. A 416-bp sequence lies directly between this gap

and the 3' UTR match (Supplementary Fig. S15). Since *fibin* is a single exon gene in most well-annotated genomes, this sequence was expected to match *fibin* and was aligned against the nr/nt, refseq-rna, refseq-genomic, HTGS, and wsg nucleotide collections using Tblastx (http://blast.ncbi.nlm.nih.gov/Blast.cgi). We retrieved no significant similarities (all blast hits had an e-value higher than 1).

We further designed primers based on the chicken and ostrich (partial) *fibin* coding sequences and Sanger-sequenced 276 bp of the kiwi AT5 *fibin* genomic sequence and kiwi 1612 *fibin* cDNA. After the partial sequence retrieval we aligned the transcriptomic reads from individual 16-12 using BWA (Version: 0.5.10) [36]. We used BLASTN to align with higher sensitivity the entire raw transcriptomic reads. Reads that aligned at the edges of the sequence with 100% identity and a hit length of at least 20 bp were collected and assembled manually using the chicken *fibin* coding sequence as reference. After four similar iterations we reconstructed the entire coding sequence of the *fibin* in kiwi. Transcriptomic reads were realigned using BWA to the final complete sequence and the alignment was visualized using IGV (version 2.3.25) [86] to proof the correctness of the assembly.

We proceeded further by testing the selective pressures acting on this gene. We first tested selection as described above using sequences from: kiwi, chicken, zebra finch, and turkey. The ω ratio for all branches (model = 0, one ratio) in the phylogeny was 1.35. We then fixed the ω to the neutral value of 1 on the one ratio branch model (model = 0, NSsites = 0, fix_omega = 1, omega = 1). LRT between the fix_omega-model and the one where ω was estimated was 1.53 (1 degree of freedom) failed to reach significance. Thus, the calculated ω is not significantly > 1 on all tested branches.

Given the signs of positive selection in the preliminary analysis, extended selection analysis was performed using 15 species: human, mouse, bat, whale, dolphin, turtle, lizard, python, flycatcher, chicken, zebra finch, frog, zebrafish, and pufferfish. We further employed the branch model and branch-site models (CODEML). First, the one-ratio

model (model = 0) was used to preliminarily estimate the average ω value on all tested species. Then, the two-ratio model (model = 2) was used to detect selective pressure acting on particular branches. These two models were compared between each other, and, also, they were separately compared to their respective null models (ω = 1 for all lineages or for the foreground branch, respectively). We also used the free ratio model, which allows ω variation among branches, to estimate the ω value on each branch and detect different selective pressures. This revealed human and zebra finch having an estimated ω = 999 (NA), which is indicative for lack of estimate given too few sites with synonymous exchanges [9]. Thus, further analyses were performed after removing these two species from the phylogeny and alignment.

Appointing each of the species in the phylogeny as the foreground branch failed to reach significance. We thus appointed multiple species (with estimated ω > 1 in the free ratio model) as the foreground branch. This revealed a faster evolution signal in python, mouse, bat, kiwi, zebrafish and pufferfish (LRT = 4.186, p-value = 0.04, $\omega_{background}$ = 1.07, $\omega_{foreground}$ = 2.13). The LRT to the model where ω is fixed to the neutral value of 1 was significant (LRT = 6.89, p-value = 0.009) (.).

Lastly, since positive selection can often act on a few sites and in a short period of evolutionary time, we performed branch-site analysis with CODEML, comparing a model where some sites are allowed to have ω > 1 and ω can vary between sets of branches (model = 2, NSsites = 2) to the null model (model = 2, NSsites = 2, fix_omega = 1, omega = 1).

These models were used to test for positive selection on a small number of sites along the different branches. Sites with selection signatures were found in bat, frog, kiwi, lizard, turtle, and zebrafish (.).

Ultra-conserved non-coding elements analysis

The set of 4,351 UCNEs were downloaded from UCNEbase [12]. UCNEs are defined as non-coding human DNA regions with ≥ 95% sequence identity between human and chicken and > 200bp length. The sequence identity threshold corresponds to a base substitution rate of approximately 1% per 100 million years [87]. The length of the UCNEs ranges from 200–1419 bp with a mean = 325 bp and a median = 283 bp. The total length is 1.4 Mb [12].

We used Blast 2.2.25 [28] with "blastn" and default parameters to retrieve the orthologous regions in *Apteryx mantelli,* and *Struthio camelus, Tinamus guttatus, Tyto alba, Antrostomus carolinensis* genomes, downloaded from GigaDB [17], and birds from Ensembl 74 [16] *Ficedula albicollis, Taeniopygia guttata, Anas platyrhynchos*, and *Meleagris gallopavo*. For each genome only the best blast hit according to the e-value was retained. We used *Gallus gallus* genome Ensembl 74 was as control in the orthology assignment and checked the number of mismatches to the reference UCNE. Orthologous regions from each of the species were aligned [29] to the reference UCNE and the number of mismatches between the UCNE and the target genomes were determined. The multiple fasta files and entire sets of results are deposited at https://bioinf.eva.mpg.de/KIWI-UCNEs/. UCNEs with higher number of changes than the expected 5% are presented in Supplementary Table S13.

Supplementary References

1. Kelley DR, Schatz MC, Salzberg SL: **Quake: quality-aware detection and correction of sequencing errors.** *Genome Biol* 2010, **11:**R116.
2. De Bie T, Cristianini N, Demuth JP, Hahn MW: **CAFE: a computational tool for the study of gene family evolution.** *Bioinformatics* 2006, **22:**1269-71.
3. Bindea G, Mlecnik B, Hackl H, Charoentong P, Tosolini M, Kirilovsky A, Fridman WH, Pages F, Trajanoski Z, Galon J: **ClueGO: a Cytoscape plug-in to decipher functionally grouped gene ontology and pathway annotation networks.** *Bioinformatics* 2009, **25:**1091-3.
4. Tanaka M: **Molecular and evolutionary basis of limb field specification and limb initiation.** *Dev Growth Differ* 2013, **55:**149-63.
5. Gertz EM, Yu YK, Agarwala R, Schaffer AA, Altschul SF: **Composition-based statistics and translated nucleotide searches: improving the TBLASTN module of BLAST.** *BMC Biol* 2006, **4:**41.
6. Benson DA, Cavanaugh M, Clark K, Karsch-Mizrachi I, Lipman DJ, Ostell J, Sayers EW: **GenBank.** *Nucleic Acids Res* 2013, **41:**D36-42.
7. Prohaska SJ, Fried C, Flamm C, Wagner GP, Stadler PF: **Surveying phylogenetic footprints in large gene clusters: applications to Hox cluster duplications.** *Mol Phylogenet Evol* 2004, **31:**581-604.
8. Pascual-Anaya J, D'Aniello S, Kuratani S, Garcia-Fernandez J: **Evolution of Hox gene clusters in deuterostomes.** *BMC Dev Biol* 2013, **13:**26.
9. Yang Z: **PAML 4: phylogenetic analysis by maximum likelihood.** *Mol Biol Evol* 2007, **24:**1586-91.
10. Wilgenbusch JC, Swofford D: **Inferring evolutionary trees with PAUP*.** *Curr Protoc Bioinformatics* 2003, **Chapter 6:**Unit 6 4.
11. Jetz W, Thomas GH, Joy JB, Hartmann K, Mooers AO: **The global diversity of birds in space and time.** *Nature* 2012, **491:**444-8.
12. Dimitrieva S, Bucher P: **UCNEbase--a database of ultraconserved non-coding elements and genomic regulatory blocks.** *Nucleic Acids Res* 2013, **41:**D101-9.

13. Drummond AJ, Rambaut A: **BEAST: Bayesian evolutionary analysis by sampling trees.** *BMC Evol Biol* 2007, **7:**214.

14. Crooks GE, Hon G, Chandonia JM, Brenner SE: **WebLogo: a sequence logo generator.** *Genome Res* 2004, **14:**1188-90.

15. Holzapfel S, Robertson HA, McLennan JA, Sporle W, Hackwell K, Impey M: **Kiwi (Apteryx spp.) recovery plan.** *Threatened Species Recovery Plan* 2008, **60**.

16. Flicek P, Ahmed I, Amode MR, Barrell D, Beal K, Brent S, Carvalho-Silva D, Clapham P, Coates G, Fairley S, et al: **Ensembl 2013.** *Nucleic Acids Res* 2013, **41:**D48-55.

17. Sneddon TP, Zhe XS, Edmunds SC, Li P, Goodman L, Hunter CI: **GigaDB: promoting data dissemination and reproducibility.** *Database (Oxford)* 2014, **2014:**bau018.

18. Li H, Coghlan A, Ruan J, Coin LJ, Heriche JK, Osmotherly L, Li R, Liu T, Zhang Z, Bolund L, et al: **TreeFam: a curated database of phylogenetic trees of animal gene families.** *Nucleic Acids Res* 2006, **34:**D572-80.

19. Prüfer K, Muetzel B, Do HH, Weiss G, Khaitovich P, Rahm E, Pääbo S, Lachmann M, Enard W: **FUNC: a package for detecting significant associations between gene sets and ontological annotations.** *BMC Bioinform* 2007, **8:**41.

20. Bowers M, Eng L, Lao Z, Turnbull RK, Bao X, Riedel E, Mackem S, Joyner AL: **Limb anterior-posterior polarity integrates activator and repressor functions of GLI2 as well as GLI3.** *Dev Biol* 2012, **370:**110-24.

21. Church VL, Francis-West P: **Wnt signalling during limb development.** *Int J Dev Biol* 2002, **46:**927-36.

22. Gao Y, Lan Y, Liu H, Jiang R: **The zinc finger transcription factors Osr1 and Osr2 control synovial joint formation.** *Dev Biol* 2011, **352:**83-91.

23. Geetha-Loganathan P, Nimmagadda S, Christ B, Huang R, Scaal M: **Ectodermal Wnt6 is an early negative regulator of limb chondrogenesis in the chicken embryo.** *BMC Dev Biol* 2010, **10:**32.

24. Koshiba-Takeuchi K, Takeuchi JK, Arruda EP, Kathiriya IS, Mo R, Hui CC, Srivastava D, Bruneau BG: **Cooperative and antagonistic interactions**

between Sall4 and Tbx5 pattern the mouse limb and heart. *Nat Genet* 2006, **38:**175-83.

25. Rallis C, Bruneau BG, Del Buono J, Seidman CE, Seidman JG, Nissim S, Tabin CJ, Logan MP: **Tbx5 is required for forelimb bud formation and continued outgrowth.** *Development* 2003, **130:**2741-51.
26. Rallis C, Del Buono J, Logan MP: **Tbx3 can alter limb position along the rostrocaudal axis of the developing embryo.** *Development* 2005, **132:**1961-70.
27. Tanaka M, Cohn MJ, Ashby P, Davey M, Martin P, Tickle C: **Distribution of polarizing activity and potential for limb formation in mouse and chick embryos and possible relationships to polydactyly.** *Development* 2000, **127:**4011-21.
28. Altschul SF, Gish W, Miller W, Myers EW, Lipman DJ: **Basic local alignment search tool.** *J Mol Biol* 1990, **215:**403-10.
29. Edgar RC: **MUSCLE: multiple sequence alignment with high accuracy and high throughput.** *Nucleic Acids Res* 2004, **32:**1792-7.
30. Renaud G, Stenzel U, Kelso J: **leeHom: adaptor trimming and merging for Illumina sequencing reads.** *Nucleic Acids Res* 2014, **42:**e141.
31. Marcais G, Kingsford C: **A fast, lock-free approach for efficient parallel counting of occurrences of k-mers.** *Bioinformatics* 2011, **27:**764-70.
32. Luo R, Liu B, Xie Y, Li Z, Huang W, Yuan J, He G, Chen Y, Pan Q, Liu Y, et al: **SOAPdenovo2: an empirically improved memory-efficient short-read de novo assembler.** *GigaScience* 2012, **1:**18.
33. Harris RS: **Improved pairwise alignment of genomic DNA. .** The Pennsylvania State University, 2007.
34. Kuhn RM, Karolchik D, Zweig AS, Wang T, Smith KE, Rosenbloom KR, Rhead B, Raney BJ, Pohl A, Pheasant M, et al: **The UCSC Genome Browser Database: update 2009.** *Nucleic Acids Res* 2009, **37:**D755-61.
35. Zhou Q, Zhang J, Bachtrog D, An N, Huang Q, Jarvis ED, Gilbert MT, Zhang G: **Complex evolutionary trajectories of sex chromosomes across bird taxa.** *Science* 2014, **346:**1246338.
36. Li H, Durbin R: **Fast and accurate short read alignment with Burrows-Wheeler transform.** *Bioinformatics* 2009, **25:**1754-60.

37. Miskelly CM, Dowding JE, Elliott GP, Hitchmough RA, Powlesland RG, Robertson HA, Sagar PM, Scofield RP, Taylor GA: **Conservation status of New Zealand birds, 2008.** *Notornis* 2008, **55:**117-35.
38. Pop M, Phillippy A, Delcher AL, Salzberg SL: **Comparative genome assembly.** *Brief Bioinform* 2004, **5:**237-48.
39. Cantarel BL, Korf I, Robb SM, Parra G, Ross E, Moore B, Holt C, Sanchez Alvarado A, Yandell M: **MAKER: an easy-to-use annotation pipeline designed for emerging model organism genomes.** *Genome Res* 2008, **18:**188-96.
40. Jurka J, Kapitonov VV, Pavlicek A, Klonowski P, Kohany O, Walichiewicz J: **Repbase Update, a database of eukaryotic repetitive elements.** *Cytogenet Genome Res* 2005, **110:**462-7.
41. Stanke M, Keller O, Gunduz I, Hayes A, Waack S, Morgenstern B: **AUGUSTUS: ab initio prediction of alternative transcripts.** *Nucleic Acids Res* 2006, **34:**W435-9.
42. Moreno-Hagelsieb G, Latimer K: **Choosing BLAST options for better detection of orthologs as reciprocal best hits.** *Bioinformatics* 2008, **24:**319-24.
43. Yang Z: **PAML: a program package for phylogenetic analysis by maximum likelihood.** *Comput Appl Biosci* 1997, **13:**555-6.
44. Yang Z: *Computational Molecular Evolution.* Oxford: Oxford University Press; 2006.
45. Nam K, Mugal C, Nabholz B, Schielzeth H, Wolf JB, Backstrom N, Kunstner A, Balakrishnan CN, Heger A, Ponting CP, et al: **Molecular evolution of genes in avian genomes.** *Genome Biol* 2010, **11:**R68.
46. Schneider A, Souvorov A, Sabath N, Landan G, Gonnet GH, Graur D: **Estimates of positive Darwinian selection are inflated by errors in sequencing, annotation, and alignment.** *Genome Biol Evol* 2009, **1:**114-8.
47. Hall TA: **BioEdit: a user-friendly biological sequence alignment editor and analysis program for Windows 95/98/NT.** *Nucleic Acids Symp Ser* 1999, **41:**95-8.

48. Finn RD, Clements J, Eddy SR: **HMMER web server: interactive sequence similarity searching.** *Nucleic Acids Res* 2011, **39:**W29-37.
49. Leschner J, Wennerberg G, Feierler J, Bermudez M, Welte B, Kalatskaya I, Wolber G, Faussner A: **Interruption of the ionic lock in the bradykinin B2 receptor results in constitutive internalization and turns several antagonists into strong agonists.** *J Pharmacol Exp Ther* 2013, **344:**85-95.
50. Zelenitsky DK, Therrien F, Ridgely RC, McGee AR, Witmer LM: **Evolution of olfaction in non-avian theropod dinosaurs and birds.** *Proc Biol Sci* 2011, **278:**3625-34.
51. Degnan JH, DeGiorgio M, Bryant D, Rosenberg NA: **Properties of consensus methods for inferring species trees from gene trees.** *Syst Biol* 2009, **58:**35-54.
52. Cho YS, Hu L, Hou H, Lee H, Xu J, Kwon S, Oh S, Kim HM, Jho S, Kim S, et al: **The tiger genome and comparative analysis with lion and snow leopard genomes.** *Nat Commun* 2013, **4:**2433.
53. Huang Y, Li Y, Burt DW, Chen H, Zhang Y, Qian W, Kim H, Gan S, Zhao Y, Li J, et al: **The duck genome and transcriptome provide insight into an avian influenza virus reservoir species.** *Nat Genet* 2013, **45:**776-83.
54. Zhang G, Li C, Li Q, Li B, Larkin DM, Lee C, Storz JF, Antunes A, Greenwold MJ, Meredith RW, et al: **Comparative genomics reveals insights into avian genome evolution and adaptation.** *Science* 2014, **346:**1311-20.
55. Kall L, Krogh A, Sonnhammer EL: **An HMM posterior decoder for sequence feature prediction that includes homology information.** *Bioinformatics* 2005, **21 Suppl 1:**i251-7.
56. Price MN, Dehal PS, Arkin AP: **FastTree 2--approximately maximum-likelihood trees for large alignments.** *PLoS One* 2010, **5:**e9490.
57. Hasegawa M, Kishino H, Yano T: **Dating of the human-ape splitting by a molecular clock of mitochondrial DNA.** *J Mol Evol* 1985, **22:**160-74.
58. Hackett SJ, Kimball RT, Reddy S, Bowie RC, Braun EL, Braun MJ, Chojnowski JL, Cox WA, Han KL, Harshman J, et al: **A phylogenomic study of birds reveals their evolutionary history.** *Science* 2008, **320:**1763-8.

59. Loytynoja A, Goldman N: **Phylogeny-aware gap placement prevents errors in sequence alignment and evolutionary analysis.** *Science* 2008, **320:**1632-5.
60. Bunce M, Worthy TH, Phillips MJ, Holdaway RN, Willerslev E, Haile J, Shapiro B, Scofield RP, Drummond A, Kamp PJ, Cooper A: **The evolutionary history of the extinct ratite moa and New Zealand Neogene paleogeography.** *Proc Natl Acad Sci U S A* 2009, **106:**20646-51.
61. Baker AJ, Haddrath O, McPherson JD, Cloutier A: **Genomic support for a Moa-Tinamou clade and adaptive morphological convergence in flightless ratites.** *Mol Biol Evol* 2014, **31:**1-38.
62. Cooper A, Lalueza-Fox C, Anderson S, Rambaut A, Austin J, Ward R: **Complete mitochondrial genome sequences of two extinct moas clarify ratite evolution.** *Nature* 2001, **409:**704-7.
63. Cooper A, Mourer-Chauviré C, Chambers GK, von Haeseler A, Wilson AC, Pääbo S: **Independent origins of New Zealand moas and kiwis.** *Proc Natl Acad Sci U S A* 1992, **89:**8741-4.
64. Mitchell KJ, Llamas B, Soubrier J, Rawlence NJ, Worthy TH, Wood J, Lee MS, Cooper A: **Ancient DNA reveals elephant birds and kiwi are sister taxa and clarifies ratite bird evolution.** *Science* 2014, **344:**898-900.
65. Wiens JJ, Kuczynski CA, Stephens PR: **Discordant mitochondrial and nuclear gene phylogenies in emydid turtles: implications for speciation and conservation.** *Biological Journal of the Linnean Society* 2010, **99:**445-61.
66. McCallum J, Hall S, Lissone I, Anderson J, Huynen L, Lambert DM: **Highly informative ancient DNA 'snippets' for New Zealand moa.** *PLoS One* 2013, **8:**e50732.
67. Hartnup K, Huynen L, Te Kanawa R, Shepherd LD, Millar CD, Lambert DM: **Ancient DNA recovers the origins of Maori feather cloaks.** *Mol Biol Evol* 2011, **28:**2741-50.
68. Ohuchi H, Yamashita T, Tomonari S, Fujita-Yanagibayashi S, Sakai K, Noji S, Shichida Y: **A non-mammalian type opsin 5 functions dually in the photoreceptive and non-photoreceptive organs of birds.** *PLoS One* 2012, **7:**e31534.

69. Kang SW, Kuenzel WJ: **Deep-brain photoreceptors (DBPs) involved in the photoperiodic gonadal response in an avian species, Gallus gallus.** *Gen Comp Endocrinol* 2015, **211:**106-13.
70. Shannon CE: **The mathematical theory of communication.** *The Bell system technical Journal* 1948, **27:**379-243 & 623-56.
71. Steiger SS, Kuryshev VY, Stensmyr MC, Kempenaers B, Mueller JC: **A comparison of reptilian and avian olfactory receptor gene repertoires: species-specific expansion of group gamma genes in birds.** *BMC Genomics* 2009, **10:**446.
72. Ballesteros JA, Jensen AD, Liapakis G, Rasmussen SG, Shi L, Gether U, Javitch JA: **Activation of the beta 2-adrenergic receptor involves disruption of an ionic lock between the cytoplasmic ends of transmembrane segments 3 and 6.** *J Biol Chem* 2001, **276:**29171-7.
73. Tamura K, Peterson D, Peterson N, Stecher G, Nei M, Kumar S: **MEGA5: molecular evolutionary genetics analysis using maximum likelihood, evolutionary distance, and maximum parsimony methods.** *Mol Biol Evol* 2011, **28:**2731-9.
74. Shannon C, Weaver W: *The Mathematical Theory of Communication.* University of Illinois Press; 2002.
75. Demuth JP, De Bie T, Stajich JE, Cristianini N, Hahn MW: **The evolution of mammalian gene families.** *PLoS One* 2006, **1:**e85.
76. Katoh K, Standley DM: **MAFFT multiple sequence alignment software version 7: improvements in performance and usability.** *Mol Biol Evol* 2013, **30:**772-80.
77. Vogel R, Mahalingam M, Ludeke S, Huber T, Siebert F, Sakmar TP: **Functional role of the "ionic lock"--an interhelical hydrogen-bond network in family A heptahelical receptors.** *J Mol Biol* 2008, **380:**648-55.
78. Gilad Y, Przeworski M, Lancet D: **Loss of olfactory receptor genes coincides with the acquisition of full trichromatic vision in primates.** *PLoS Biol* 2004, **2:**E5.

79. Kondo R, Kaneko S, Sun H, Sakaizumi M, Chigusa SI: **Diversification of olfactory receptor genes in the Japanese medaka fish, Oryzias latipes.** *Gene* 2002, **282:**113-20.
80. Singer MS, Weisinger-Lewin Y, Lancet D, Shepherd GM: **Positive selection moments identify potential functional residues in human olfactory receptors.** *Recept Channels* 1996, **4:**141-7.
81. Anisimova M, Liberles DA: **The quest for natural selection in the age of comparative genomics.** *Heredity (Edinb)* 2007, **99:**567-79.
82. Litwin S, Jores R: **Shannon information as a measure of amino acid diversity.** In *Theoretical and experimental insights into immunology. Volume* 66. Edited by Perelson AS, Weisbuch G. Berlin: Springer Berlin Heidelberg; 1992: 279-87: *NATO ASI Series*].
83. Richardson MK, Crooijmans RP, Groenen MA: **Sequencing and genomic annotation of the chicken (Gallus gallus) Hox clusters, and mapping of evolutionarily conserved regions.** *Cytogenet Genome Res* 2007, **117:**110-9.
84. Wagner GP, Fried C, Prohaska SJ, Stadler PF: **Divergence of conserved non-coding sequences: rate estimates and relative rate tests.** *Mol Biol Evol* 2004, **21:**2116-21.
85. Wakahara T, Kusu N, Yamauchi H, Kimura I, Konishi M, Miyake A, Itoh N: **Fibin, a novel secreted lateral plate mesoderm signal, is essential for pectoral fin bud initiation in zebrafish.** *Dev Biol* 2007, **303:**527-35.
86. Thorvaldsdottir H, Robinson JT, Mesirov JP: **Integrative Genomics Viewer (IGV): high-performance genomics data visualization and exploration.** *Brief Bioinform* 2013, **14:**178-92.
87. Retelska D, Beaudoing E, Notredame C, Jongeneel CV, Bucher P: **Vertebrate conserved non coding DNA regions have a high persistence length and a short persistence time.** *BMC Genomics* 2007, **8:**398.

URLs

Bird tree, <http://birdtree.org/>;

Genome size browser, <http://www.genomesize.com>;

Avian Phylogenomics Project, <http://avian.genomics.cn/en/)>;

AMOScmp-shortReads, <http://sourceforge.net/projects/amos/files/>;

RepeatMasker Open-3.0., <http://www.repeatmasker.org>;

PHYLIP, <http://evolution.genetics.washington.edu/phylip.html>;

Pfam, <ftp://ftp.sanger.ac.uk/pub/databases/Pfam/Tools/>;

MAFFT v7, <http://mafft.cbrc.jp/alignment/server/>;

FigTree 1.3.1, <http://tree.bio.ed.ac.uk/software/figtree/>;

Ultra-conserved non-coding elements and genomic regulatory blocks, <http://ccg.vital-it.ch/UCNEbase>.
